# G protein-coupled receptors (GPCRs): advances in structures, mechanisms and drug discovery

**DOI:** 10.1038/s41392-024-01803-6

**Published:** 2024-04-10

**Authors:** Mingyang Zhang, Ting Chen, Xun Lu, Xiaobing Lan, Ziqiang Chen, Shaoyong Lu

**Affiliations:** 1 Key Laboratory of Protection, Development and Utilization of Medicinal Resources in Liupanshan Area, Ministry of Education, Peptide & Protein Drug Research Center, School of Pharmacy, Ningxia Medical University, Yinchuan, 750004 China; 2 Medicinal Chemistry and Bioinformatics Center, Shanghai Jiao Tong University School of Medicine, Shanghai, 200025 China; 3 Department of Cardiology, Changzheng Hospital, Affiliated to Naval Medical University, Shanghai, 200003 China; 4 Department of Orthopedics, Changhai Hospital, Affiliated to Naval Medical University, Shanghai, 200433 China

**Keywords:** Target identification, Target identification

## Abstract

G protein-coupled receptors (GPCRs), the largest family of human membrane proteins and an important class of drug targets, play a role in maintaining numerous physiological processes. Agonist or antagonist, orthosteric effects or allosteric effects, and biased signaling or balanced signaling, characterize the complexity of GPCR dynamic features. In this study, we first review the structural advancements, activation mechanisms, and functional diversity of GPCRs. We then focus on GPCR drug discovery by revealing the detailed drug-target interactions and the underlying mechanisms of orthosteric drugs approved by the US Food and Drug Administration in the past five years. Particularly, an up-to-date analysis is performed on available GPCR structures complexed with synthetic small-molecule allosteric modulators to elucidate key receptor-ligand interactions and allosteric mechanisms. Finally, we highlight how the widespread GPCR-druggable allosteric sites can guide structure- or mechanism-based drug design and propose prospects of designing bitopic ligands for the future therapeutic potential of targeting this receptor family.

## Introduction

G protein-coupled receptors (GPCRs) are the largest superfamily of cell surface membrane receptors and are encoded by approximately 1000 genes, sharing conserved seven-transmembrane (7TM) helices connected by three intra- and three extra-cellular loops.^1–3^ GPCRs are conformationally dynamic proteins that mediate vital biological functions of signal transduction triggered by various extracellular signals such as photons, ions, lipids, neurotransmitters, hormones, peptides, and odorants.^4–8^ Due to the distinct topography between the binding sites of extracellular stimuli and the subsequent signaling events at the intracellular site (approximately 40 Å), GPCR signal transduction is allosteric.^9–13^ Advances in protein engineering, X-ray crystallography, and cryo-electron microscopy (cryo-EM), coupled with innovative technologies such as X-ray free electron lasers (XFELs) and nuclear magnetic resonance (NMR) spectroscopy, have revolutionized our understanding of GPCR structures and dynamics. These studies provide insights into ligand-receptor interactions, conformational changes, and signaling complexes, offering unprecedented opportunities for in-depth investigations into receptor activation, orthosteric/allosteric modulation, biased signaling, and dimerization.

Once activated by exogenous stimuli, GPCRs primarily employ heterotrimeric G-proteins and arrestins as transducers to produce second messengers and further initiate the downstream signaling, resulting in promiscuous signaling profiles within cells.^11^ Such spectrum of signaling is the prerequisite for function diversity of GPCRs and is fundamental in regulating physiological processes, including sensory perception, neurotransmission, and endocrine processes.^14,15^ The mutations and truncation of GPCRs; however, can dysregulate GPCR functionality by altering constitutive activity, influencing membrane expression and affecting post-translational behaviors.^16^ Unraveling the mechanisms of stimuli-GPCR-effector coupling, as well as the concise regulation of GPCR dysfunction will bring about valuable therapeutic potentials and inspire the design of modulators with high potency, selectivity, or biased signaling.

**Fig. 1:**
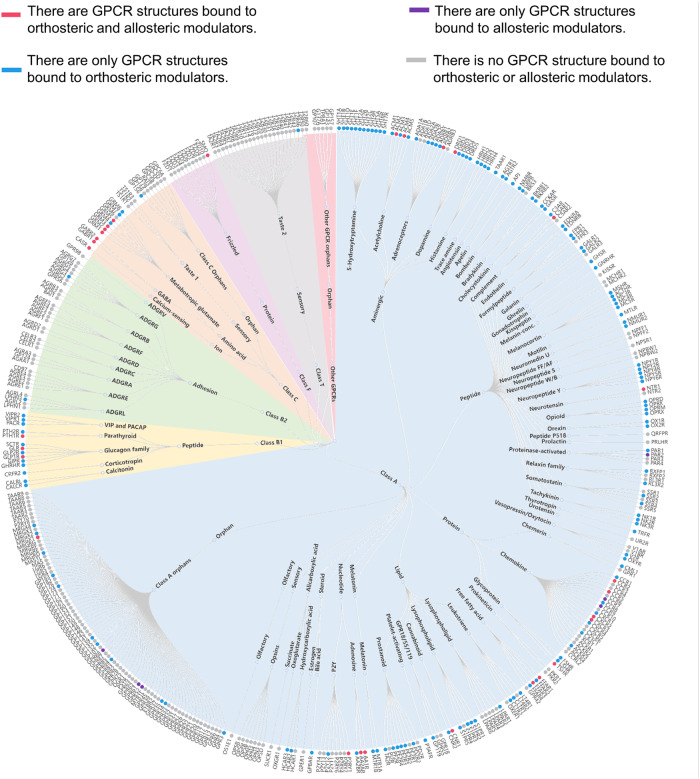
Phylogenetic tree of GPCRs indicating GPCR structures that have been solved in complex with modulators. Nodes represent GPCRs named according to their UniProt gene name and are organized according to the GPCR database. GPCR structures bound to modulators are highlighted by color

Till date, approximately 34% of the US Food and Drug Administration (FDA)-approved drugs are targeted to GPCRs, with modulators in clinical trials or preclinical stages experiencing exponential growth.^17,18^ Among them, orthosteric ligands impose an effective alteration on GPCR activity and signaling process by competitively preventing the binding of endogenous ligands.^19^ However, due to the sequence conservation of orthosteric sites, in most cases, subtype selectivity remains an intractable issue, which implies the inevitable side effects of orthosteric drugs.^20^ As an alternative or complementary option, targeting allosteric sites alone or targeting both orthosteric and allosteric sites can overcome these major hurdles.^21–25^ Allosteric modulators are highlighted for their high subtype selectivity and low side effects. A progressive structural understanding of the detailed receptor-ligand interactions is paving the way for fragment-to-lead optimization in structure-based drug design (SBDD) (Fig. 1). Moreover, the knowledge of allosteric sites is useful for the design of bitopic ligands by creating a molecule attached to both an allosteric and orthosteric site. Bitopic ligands have several advantages of improved affinity and enhanced selectivity over a single allosteric or orthosteric ligand. In addition, elucidating allosteric mechanisms of GPCRs provides a viable strategy to develop biased ligands such as G protein- or β-arrestin-based allosteric modulators.^26^ Bitopic modulators have higher selectivity to reduce side effects since they exert pathway-specific effects on GPCR signaling.^26,27^

In this review, we first summarize the structural progression, activation mechanisms, and functional diversity of GPCRs. To delve into the advancement of GPCR drug discovery, we investigate the detailed drug-target interactions at the orthosteric sites, focusing on GPCR structures in complex with recent FDA-approved orthosteric drugs. Subsequently, allosteric modulators are extensively discussed, with a focus on recent breakthroughs in GPCR structures that bind to synthetic small molecules. Notably, peptides and antibodies are excluded from our analysis. Such investigation systematically clusters the location of allosteric sites in the extracellular vestibule, transmembrane domain, and intracellular surface, highlighting the key binding modes with their target receptors and allosteric mechanisms. This review aims to provide a deeper understanding of GPCR structures, mechanisms, and drug discovery, which has important implications for structure- or mechanism-based drug design and the design of bitopic ligands for the future therapeutic potential of targeting this receptor family.

## Structure advances in GPCRS

The low expression of membrane protein GPCRs, combined with their conformational flexibility, initially posed great challenges for high-resolution diffraction.^28^ The initial crystal structures of rhodopsin and the ligand-activated β2 adrenergic receptor (β2AR) were resolved in 2000 and 2007, respectively.^29,30^ Over the past two decades, considerable progress has been made in the engineering of proteins and the technique of X-ray crystallography.^31^ Notably, the use of GPCR engineering with fusion proteins,^32,33^ antibody fragment crystallization^34,35^, and thermostabilizing mutations^36^, has produced numerous antagonist- or agonist-bound GPCR structures. However, only agonist-bound GPCRs frequently exist in an intermediate conformation because the fully active conformation requires stabilizing chaperones, including G proteins, G protein mimetics, conformationally specific nanobodies, and mini-G proteins.^37^

**Fig. 2:**
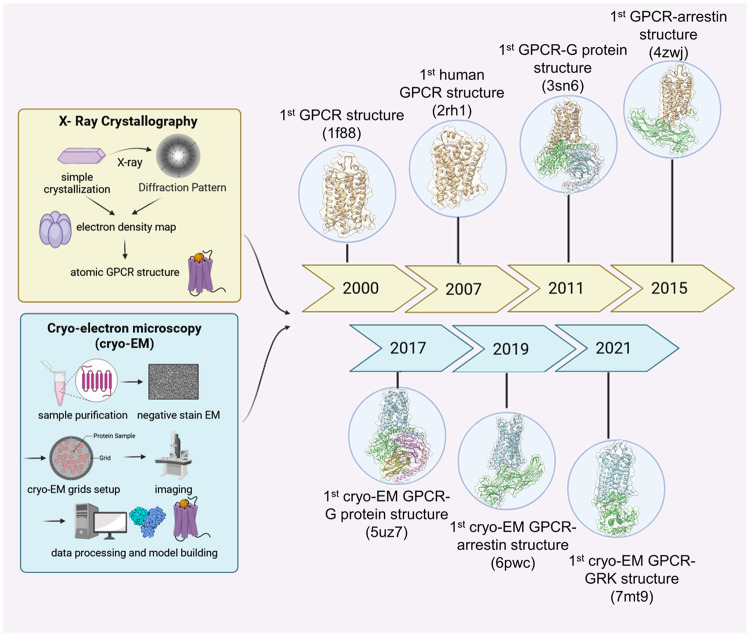
Timeline of major advancements in GPCR structure study using X-ray crystallography and cryo-EM

The first GPCR-G protein complex was determined in 2011 using X-ray diffraction;^38^ however, the demanding nature of X-ray crystallography has rendered GPCR-G protein complex crystallization a difficult undertaking. Cryo-EM has developed to be an alternative technique, driving a novel trend in GPCR structural biology. Unlike X-ray crystallography, cryo-EM does not rely on crystals and has considerably superior potential to directly visualize detergent- or nanodisc-solubilized GPCRs. This capability enables the determination of previously intractable fully active states and larger protein complexes, including GPCR-G protein complexes.^39^ Since then, the number of cryo-EM structures depicting GPCRs in complex with intracellular partners has experienced exponential growth (Fig. 2). As of November 2023, the Protein Data Bank has accumulated 554 complex structures, of which 523 are resolved using cryo-EM.^40^ However, both crystallography and cryo-EM are limited to capturing the most stable and lowest energy conformations under crystallization conditions.^4^ Moreover, the comprehensive characterization of intermediate states and transition kinetics remains elusive. Crystallographic, spectroscopic, and simulation techniques have offered complementary information on the conformational dynamics of GPCRs.

The advanced XFELs possess the potential to solve the missing information. The exceptional properties of XFELs, characterized by extreme brilliance and femtosecond short pulses, allow them to overcome radiation damage, facilitating the determination of GPCR structures with atomic-level information at femtosecond timescales.^41^ NMR spectroscopy offers a valuable technique to detect dynamic features of GPCRs in liquid environments.^42,43^ The number, position, and shape of signals in the NMR spectra are sensitive to changes in the micro-environment of stable-isotope “probes” incorporated into receptors. Double electron-electron resonance (DEER) spectroscopy enables the assessment of a distance distribution between two different probes. Fluorescence resonance energy transfer (FRET), a technique based on fluorescence, functions as an “atomic ruler” to detect the proximity between two labels, providing valuable data about the number of states and their relative populations.^44,45^ Among these, DEER and FRET provide only localized details regarding the chemical probes that have been inserted. In addition, molecular dynamics (MD) simulations offer a comprehensive, time-resolved view of complete protein structures, capturing intermediate states along the transition pathway.^46–48^ Advances in the structural biology of GPCRs have revealed key information on ligand-receptor interactions, conformational changes, and signaling complexes, opening the opportunity for exploration of receptor activation, orthosteric/allosteric modulation, biased signaling, and dimerization.

## Mechanism of GPCR activation and signaling

Although the nature of GPCRs and activating stimuli may vary significantly, GPCRs primarily coordinate distinct downstream signaling responses through two types of transducers: heterotrimeric G proteins and arrestins. Human G proteins comprise four major families (G_s_, G_i/o_, G_q/11,_ and G_12/13_) and more than half of GPCRs activate two or more G proteins, each of which exhibits distinct efficacies and kinetics.^49,50^ The promiscuous coupling leads to fingerprint-like signaling profiles inside the cell, which contributes to the complexity of GPCR signaling.

When bound to GDP, the Gαβγ heterotrimer is inactive. Agonist binding leads to the formation of an active conformation of GPCRs, which initiates signaling cascades involving the recruitment and activation of G-proteins. The activated GPCR catalyzes the GDP/GTP exchange on the Gα subunit, causing the dissociation of Gα from the Gβγ dimer. Due to high cellular concentrations of GTP, Gα rapidly binds a molecule of GTP at the nucleotide-binding site. Both Gα-GTP and Gβγ can modulate subsequent effector proteins. Gα-GTP can activate or inhibit enzymes such as adenylyl cyclase (AC), phospholipase C (PLC), or ion channels, depending on the specific G protein type. Gβγ can also modulate various signaling pathways and interact with target proteins. Activation of effector proteins by Gα-GTP or Gβγ generates second messengers, such as cyclic AMP (cAMP). The cellular response concludes with the Gα subunit hydrolyzing GTP to GDP, leading to its reassociation with Gβγ and G protein inactivation. Subsequently, the Gα subunit completes the G-protein activation cycle by reassociating with Gβγ.

**Fig. 3:**
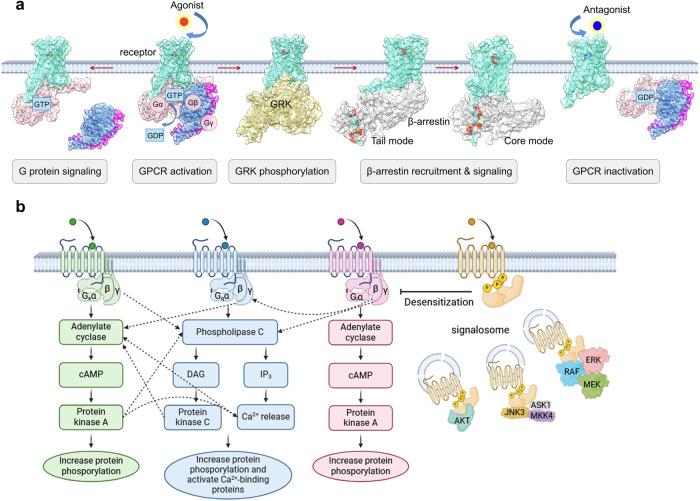
**a** Schematic representation of GPCR activation process. Upon agonist (red circle) binding, the receptor proceeds into a pre-activation state coupling with the G protein heterotrimer, where the exchange of GDP and GTP in G protein α subunit leads to G protein dissociation and mediate G protein signaling pathway. The phosphorylation of the receptor C-terminal tail by GRK binding promotes arrestin recruitment and signaling. When the antagonists (blue circle) bind, the receptor stabilizes in an inactive state. **b** Crosstalk of downstream pathway of Gs, Gq, Gi and arrestin

To prevent sustained signaling, activated GPCRs may also undergo C-terminal phosphorylation facilitated by G-protein-coupled receptor kinases (GRKs). This multi-site GPCR phosphorylation determines β-arrestin binding affinity and induces receptor desensitization via steric hindrance, followed by clathrin-mediated endocytosis and ubiquitination of the receptor (Fig. 3a).^11,51,52^ The receptor-arrestin complex also serves as a scaffold for over 20 different kinases, including mitogen-activated protein (MAP) kinases, ERK1/2, p38 kinases, and c-Jun N-terminal kinases, activating G-protein independent signaling pathway. Four isoforms of arrestin (arrestins 1-4) and multiple GRK isoforms were discovered, with arrestins 1 and 4 being only found in the visual system. β-arrestins 1 and 2, also referred to as arrestins 2 and 3, interact with and regulate numerous non-visual GPCRs.^34^

Originally classified as monomers, GPCRs were subsequently recognized to engage in homo- or hetero-dimerization, displaying distinct properties in receptor activation, pharmacological cascades, and biological functions.^53,54^ Recent research indicates that GPCRs can bind to various single transmembrane accessory proteins to regulate their biological functions such as ligand binding, transducer coupling, and intracellular signaling.^55,56^ Prominent examples include the family of receptor activity-modifying proteins (RAMPs) that majorly regulate the glucagon receptor (GCGR) and the melanocortin receptor accessory proteins (MRAPs) that regulate the melanocortin receptors (MC1R-MC5R).^57,58^ Currently, the interactions between the negative allosteric modulator RAMP2 and GCGR as well as the positive allosteric modulator MRAP1 and MC2R have been elucidated by cryo-EM.^59,60^

Structural changes within GPCRs facilitate their function as molecular conduits that transmit extracellular signals across membranes to elicit cellular responses. A distinctive feature of GPCR activation involves notable outward movement of the cytoplasmic end of TM6, creating an intracellular pocket to accommodate the downstream transducers. GPCRs contain several conserved structural motifs relevant to their activation, including the CWxP motif of TM6, the NPxxY motif of TM7, and the ionic lock that involves TM3-TM6, as well as TM3-TM7.^61–63^ Additionally, Na^+^ acts as an endogenous negative allosteric modulator (NAM) of class A GPCR activation, stabilizing the inactive state through direct interactions.^64,65^ High resolution structures reveal that Na^+^ interacts mainly with residues from TM1, TM2, TM3, and TM7 and these interactions vary across GPCRs.^66,67^

Ligands can regulate receptor activity by stabilizing distinct conformations. Since the diverse signaling pathways elicit distinct physiological effects, ligands that selectively induce beneficial pathways hold promising therapeutic value. These drugs are commonly referred to as “biased ligands.” For instance, G protein-biased μ-opioid receptor (μOR) agonists are of remarkable clinical relevance as they enhance analgesia effects and reduce adverse reactions associated with the activation of β-arrestin pathways, in contrast to morphine. Several novel biased ligands are currently in clinical use or under investigation, such as TRV130, PZM21, and SR-17018.^68–70^ Hence, unraveling the coupling mechanisms governing G proteins, GRKs, and arrestins will establish a robust foundation for designing biased ligands tailored to selectively activate or inhibit specific pathways.

In the absence of agonists, GPCRs may display different levels of constitutive activities. The efficacy of diverse ligands acting on a single GPCR in terms of activation or inactivation also varies widely. Considering both receptor constitutive activity and drug efficacy, GPCR ligands are categorized as (full) agonists, partial agonists, antagonists, and inverse agonists. These variations in efficacy significantly influence their therapeutic properties.

## Functional diversity of **GPCRS**

### Overview of GPCR subfamilies and their physiological functions

GPCRs can be categorized into class A, class B, class C, class F, and class T according to their structural and functional characteristics. Class A GPCRs, namely the rhodopsin-like family, is the superfamily with the largest proportion and the most extensive research.^71^ Class A GPCRs can further be divided by function into aminergic, peptide, protein, lipid, melatonin, nucleotide, steroid, dicarboxylic acid, sensory, and orphan subgroups,^72^ with their corresponding indications ranging from hypertension, cardiovascular diseases, and pulmonary diseases, to depression and psychiatric disorders.^17^ Class B GPCRs are divided into secretin (B1) and adhesion (B2) subfamilies, with the former characteristic of large extracellular domains (ECD) and the latter possessing a unique long N-terminal motif and autoproteolysis-inducing domain.^73^ While glucagon-like peptide-1 receptor (GLP-1R) and glucagon receptor (GCGR) are emerging as the famous B1 GPCR targets in regulating blood glucose homeostasis and lipid metabolism;^74,75^ the B2 subfamily is critical in modulating sensory, endocrine, and gastrointestinal systems.^76^ Class C GPCRs, the glutamate receptors, are unique in their large ECDs, conserved venus fly traps (VFTs), cysteine-rich domains (CRDs) on the ligand binding sites, and constitutive dimers for receptor activation.^77^ With mGluRs (metabotropic glutamate receptors) taking the lead in clinical transformation, the physiological functions of class C GPCRs are implicated in cancer, migraine, schizophrenia, and movement disorders.^77^ Class F GPCRs, comprising 10 frizzled receptors (FZDs) and one smoothened receptor (SMO), are distinctive in their conserved CRD regions and involvement in Hedgehog and Wnt signaling pathways. Therefore, they are mainly associated with cancer, fibrosis, and embryonic development.^78^ The current drug discovery is only focused on SMO,^79^ leaving broad exploration space for the therapeutic potential of FZDs. Particularly, although taste 2 receptors (TAS2Rs), the receptors modulating taste perception of humans, show structure similarity with class A GPCRs, their low sequence homology (<20%) with the existing types of GPCRs isolates them to a novel category of class T GPCRs,^76^ deepening our understanding of the entire GPCR family.

### Involvement of GPCRs in sensory perception, neurotransmission, and endocrine regulation

#### Rhodopsin, TAARs, and TASRs in sensory perception

One of the most significant physiological functions GPCRs exercise is mediating sensory information such as light perception, taste, olfaction, and pheromone sensation. Rhodopsin, which contributes to the first stage of visual activation in vertebrates, exhibits the typical and representative features of class A GPCRs. Upon absorbing photons, the orthosteric ligand of rhodopsin, retinal, experiences conformational flipping within picoseconds, thus rapidly triggering signal propagation from the receptor to G proteins, cGMP phosphodiesterase, or cGMP-gated ion channel.^80^ The covalent linkage of retinal with the receptor, and the instantaneous overturning and signaling serve as a paradigm for elucidating the efficiency of GPCRs in sensory perception.

Olfactory sensory receptors, which can be categorized into odorant receptors (ORs) and trace amine-associated receptors (TAARs), are a valuable medium for researchers to understand olfactory information encoding. Guo et al.^81^ has recently revealed the universal mechanism of TAARs recognition of amine odor molecules and the structural basis of “combinatorial coding” of the olfactory receptor in ligand recognition. Notably, the selective coupling of mTAAR9 with Gs and Golf is also delineated, which serves as a pioneer in the field of mammalian olfactory recognition. Apart from selective G-proteins, the downstream transduction mechanism of olfactory receptors is also associated with adenylyl cyclase and cAMP-gated ion channel,^82^ leaving favorable exploration opportunities.

To regulate the sensory function of taste, which is one of the most important sensations in human life, taste receptors (TASRs) are extensively studied from physiological and pharmacological perspectives. Among them, type I taste GPCRs function by forming heterodimeric complexes to stimulate sweet (TAS1R2/TAS1R3) and umami (TAS1R1/TAS1R3) sensation, whereas Type II are monomeric TAS2Rs that regulate bitter flavor.^83^ Tastant binding to the receptor activates downstream secondary messengers, resulting in depolarization and sensitizing the transient receptor potential (TRP) channel, which in turn innervates the gustatory cortex in the brain.^84^ Given the inapplicability of the previous GPCR expression techniques in TAS2Rs,^85^ overcoming difficulties in the structural determination of taste receptors will further facilitate their physiological research.

#### μOR and CBR in neurotransmission

Currently, neurological therapeutic demands mainly revolve around neuropathic pain alleviation, treatment of depression, psychiatric disorders, and Parkinson’s diseases. μ-Opioid receptors (μORs), possessing a research history of over 50 years, have been extensively researched about their mechanism of analgesic action in the peripheral nervous system (PNS) and the central nervous system (CNS). For instance, μORs reduce the release of nociceptive substances and decrease Ca^2+^ production following nerve injury by interacting with TRPV1, H1R, and NK1R in nociceptive receptors,^86^ whereas in spinal dorsal horn neurons, μORs modulate 5-HT receptors, glycine receptors, and norepinephrine receptors to activate pain inhibitory pathway.^87^ Orthosteric biased modulators, allosteric modulators, and bitopic modulators have been successively developed to exert analgesic effects while alleviating side effects like respiratory depression and addiction.^88^ Cannabinoid receptors (CBRs) are also representative targets involved in neurotransmission and neuropathic pain pathophysiology. The subtype CB1R is primarily found in presynaptic terminals of neurons in CNS, the activation of which inhibits neurotransmitter release and algesthesia transmission,^89^ while CB2R is highly expressed in immune cells, the activation of which can inhibit inflammatory factors that promote pain sensitization.^90^ No-selective orthosteric CB1R and CB2R activators can produce an antinociceptive effect and improve sleep in several animal models, while selective positive allosteric modulators (PAMs) like ZCZ011 (**40**) are rising as more promising ligands without inducing cannabis-like side effects.^91^

#### GLP-1R and GPR120 in endocrine regulation

Endocrine syndrome has been rising as one of the most critical health issues in the 21^st^ century. Numerous metabolism-related GPCRs, which are usually activated by energy metabolites or substrates, are pivotal sensors of endocrine dysregulation. GLP-1R and GPR120 (also known as free fatty acid receptor 4), for example, are both promising therapeutic targets for the treatment of type 2 diabetes and obesity.^74,92^ Mechanistically, the endogenous ligand of GLP-1R, GLP-1, can reduce the secretion of glucagon in pancreatic α cells and promote insulin secretion in pancreatic β cells. For GPR120, however, the binding of omega-3 polyunsaturated fatty acids (ω3-FAs) and receptor activation can reduce inflammation of adipose tissue and protect against insulin resistance.^93^ The receptor’s coupling with G_q/11_ subsequently stimulates the PI3K/Akt pathway, resulting in the uptake of glucose in adipocytes.^94^ As GLP-1R agonist liraglutide takes the lead in FDA-approved drugs treating type 2 diabetes and obesity,^95^ drug development of more endocrine-related targets such as GPR35, GPR40, GPR41, GPR43, GPR81, and GPR119 are supposed to come into our view.

### Receptor promiscuity and cross-talk between different signaling pathways

GPCR receptors convert the extracellular stimuli to intracellular signals to control cellular function and phenotype. GPCR receptors convert the extracellular stimuli to intracellular signals to control cellular phenotype and function. These intracellular signaling pathways intersect with each other to enhance or downgrade relevant responses in a phenomenon known as “cross-talk.” The promiscuity of the GPCR signaling network is consequently outlined, resulting in more extensive regulation, low selectivity, and possible adverse effects.

Promiscuity and cross-talk can occur at three levels, including the GPCR receptors, G-proteins/β-arrestins, and the downstream effectors. The receptor promiscuity lies in the formation of heterodimers, which can either be constituted of subtypes of the same receptor family or those of different families. A compelling case is the heterodimerization of GABA_b(1)_ and GABA_b(2)_ which leads to the functionality of modulating GIRK (G-protein gate inward rectifying channel) potassium channels, whilst neither of them is functional when expressed as a monomer.^96^ Another well-established example is the plentiful interrelationship of adenosine receptors and dopamine receptors, where the activation of A_1A_ and A_2A_ adenosine receptors decreases dopamine binding to D1 and D2 dopamine receptors.^97^ The bivalent ligands that bind adenosine receptors and dopamine receptors at each end further demonstrate the occurrence and functionality of heterodimerization.^98^ The participants of heterodimerization are assumed to share a common G-protein pool, thus contributing to the redistribution of their interaction of G-proteins and reshaping the signaling landscape.^99^ Given this, by direct cross-talk between two GPCR receptors, ligands can be designed towards one receptor to modulate the affinity and efficacy of the other target, although certain pharmacological profiles remains unclear.

At the second stage of the hierarchical signaling of GPCRs, namely the recruitment of G_s_, G_i_, G_q_, G_12_, β-arrestin 1, and β-arrestin 2, a spectrum of coupling strengths ranging from highly selective coupling to promiscuous coupling is exhibited. MD simulations performed by Sandhu et al. revealed that engineered mutant GPCRs can alter the coupling of non-cognate G-proteins by reshaping the intracellular interface,^99^ demonstrating that “dynamic structural plasticity” of the GPCR cytosolic pockets is the foundation of G-protein promiscuity. Mutants, orthosteric and allosteric modulators that exert long-range and delicate effects towards the cytosolic binding interface are therefore principal strategies to achieve selectivity of G-protein signaling.

The promiscuity of distinct downstream effectors, known as the third stage of signaling, is highly correlated with the cross-talk of G-proteins. Normally, stimulation of G_s_, G_i,_ and G_q_ results in the activation of AC, the inhibition of AC, and the stimulation of PLC, respectively.^100^ However, once distinct G-proteins are recruited near the membrane at a similar time, βγ subunits released from respective G-protein activation are “exchangeable” between diverse signaling pathways and can potentiate responses mediated by other G-proteins.^101^ The second messengers then phosphorylate, activate, or deactivate each other to construct a fine-tuning network (Fig. 3b). Albeit conducting a great deal of research, the precise control of GPCR promiscuity remains obscure.

### Impact of GPCR mutations on human diseases and therapeutic implications

**Fig. 4:**
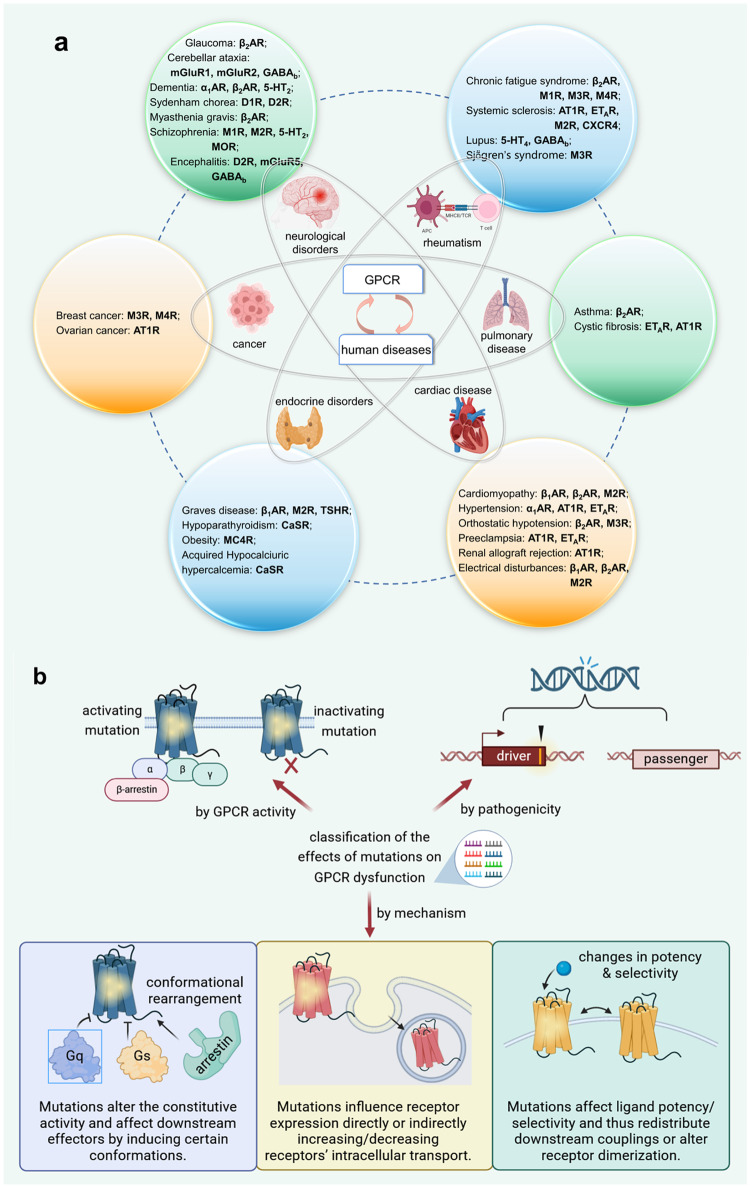
**a** Categories of Representative human diseases caused by GPCR dysfunctions. **b** Classification of the effects of mutations on GPCR dysfunctions

Besides being involved in numerous physiological processes, mutations in GPCRs can be linked to manifold human diseases, underlying the necessity of GPCR genomics, and imposing therapeutic implications. Till date, over 2350 mutations in GPCR genes have been identified as the major causes of more than 60 inherited monogenic diseases in humans (Fig. 4a), with missense mutations harboring the maximum proportion (>60%) and small inserts/deletions ranking the second (>15%).^16^

#### Classification of the effects of mutations on GPCR dysfunctions

The effects of mutations in GPCRs can be categorized into gain-of-function (GoF) and loss-of-function (LoF), corresponding to physiological hyperfunction and hypofunction, respectively. Recent studies have provided a more detailed explanation of the diverse underlying mechanisms of GoF and LoF mutations. Compared with the wild-type (WT) GPCR activation, the common pharmacological mechanisms of activating and inactivating mutations lie in three aspects: (1) Mutations transform micro-switch cascades within the receptors and induce active/inactive conformations, thus altering the constitutive activity of GPCRs and affecting the recruitment of downstream effectors. (2) Mutations influence receptor expression directly or indirectly increasing/decreasing receptors’ intracellular transport, degradation, and recycling. (3) Some mutations affect ligand potency, specificity, or promiscuous recognition, thereby exerting regulatory functions by shifting the conformational population, redistributing the downstream couplings, or altering receptor dimerization. Furthermore, all variants are not pathogenic. This provides robust evidence for another classification of “driver” and “passenger” mutations (Fig. 4b).^102^ Computational approaches are recently emerging to predict the driver ability of mutations in GPCR-related diseases, based on abundant clinical data of mutations and relevant GPCR dysfunctions.

#### Correlation of GPCR mutations and human diseases

The genomic alterations induced by GPCR mutations serve as the major driver of various monogenic diseases. Some well-established examples include missense mutations in SMO receptor causing basal cell carcinoma,^103^ missense and nonsense mutations in MC4R causing obesity^104^, and missense mutations in FSHR inducing ovarian hyperstimulation syndrome. The majority of mutations are highly conserved and thus in an advantageous position during evolution.^105^ Therefore, the pathological relevance between GPCR mutations and human diseases may be more effectively predicted taking the evolutionary conservation of a certain residue into consideration.

#### Therapeutic implications and approaches of GPCR pathologies

Terapeutic approaches of GPCR pathologies mainly include symptomatic and etiological treatment. As many GPCR dysfunctions ultimately result in endocrine diseases with end-organ resistance or cancer, the administration of hormones or chemotherapeutics may be considered to reduce pathologic phenotypes.^106,107^ More state-of-the-art therapeutic implications, however, are oriented towards etiological treatment. Missense mutations in GPCRs can mislead protein folding and post-translational modifications to cause trafficking alterations, in which pharmacological chaperones are applicative therapeutic regimens.^108^ For receptor truncation resulting from nonsense mutations or frame-shifting mutations, RNA interference, gene replacement approaches, and the genome editing approach CRISPR/Cas9 may rescue the receptor integrality, provided that at least the first three transmembrane helices remain in the mutant receptor.^109^ Designing peptides or small molecule modulators is the most straightforward means for the restoration of receptor pharmacology, though high expenditure in multiple mutations remain an intractable issue.

## Advances in GPCR drug discovery

### Overview of traditional and emerging approaches for GPCR drug discovery

Since enkephalin was first recognized as the endogenous ligand of opioid receptors,^110^ the discovery of modulators with diverse regulatory effects is constantly endowing meaning in the research of GPCRs. Several decades have witnessed the transformation from serendipity to rational design in the field of GPCR drug discovery, and the ligands have been expanded from natural products to synthesized compounds and engineered antibodies. Currently, apart from the traditional molecular docking and SBDD, more screening methodologies of wet experiments have been established to facilitate the selection of high-quality hits, including FRET/ BRET (Bioluminescence Resonance Energy Transfer) assay, NanoBiT (NanoLuc Binary Interaction Technology) assay, Tango assay, and ^19^F NMR.^111^ Once the hits were obtained, structure-activity relationship (SAR) optimization in synergistic application of computational methodologies such as fragment-growing, property prediction, and MD simulations, was conducted to initiate the hit-to-lead and lead-to-drug campaign.^99^

Herein, we specially emphasize on the interaction and signaling mechanism of synthetic small-molecule modulators bound to GPCRs, with the aim of enlightening the discovery of more ingenious molecules with high potency, selectivity, and potential biased effects.

### Structure-based drug design targeting the orthosteric sites of GPCRs

Orthosteric small molecule modulators are the most universal non-peptide regulators of GPCRs. By competing with endogenous ligands, they interact with the orthosteric binding pocket (OBP) and exert a full agonistic^112,113^/partial agonistic^114^/antagonistic function^115,116^ by triggering the conformational displacement of GPCR internal structures.^117,118^ Despite their relatively mature development, side effects derived from low subtype selectivity and promiscuous signaling remain the major hurdle.^49,119^

Over the past 30 years, the widespread use of X-ray and Cryo-EM has facilitated the characterization of GPCR-orthosteric ligand complexes, with 657 class A, 16 class B1, 6 class B2, 19 class C, 18 class F, and 1 class T structures solved (supplementary Table 1–5).^120^ Here, we meticulously selected five representative complexes in which ligands have been launched recently to elucidate the mechanisms of ligand recognition, specificity, and elaborate signaling transduction. Furthermore, we exemplified two cases to demonstrate the beneficial engagement of structural information in exploiting not only SAR but also the structure-functional selectivity relationship (SFSR). Considering these seven cases as a paradigm, we aimed to condense valuable hints based on a detailed analysis of approved drugs or selective compounds and provide a constructive outlook for the high-quality discovery of GPCR orthosteric modulators that may overcome the current dilemma.

#### μOR in complex with oliceridine

**Fig. 5:**
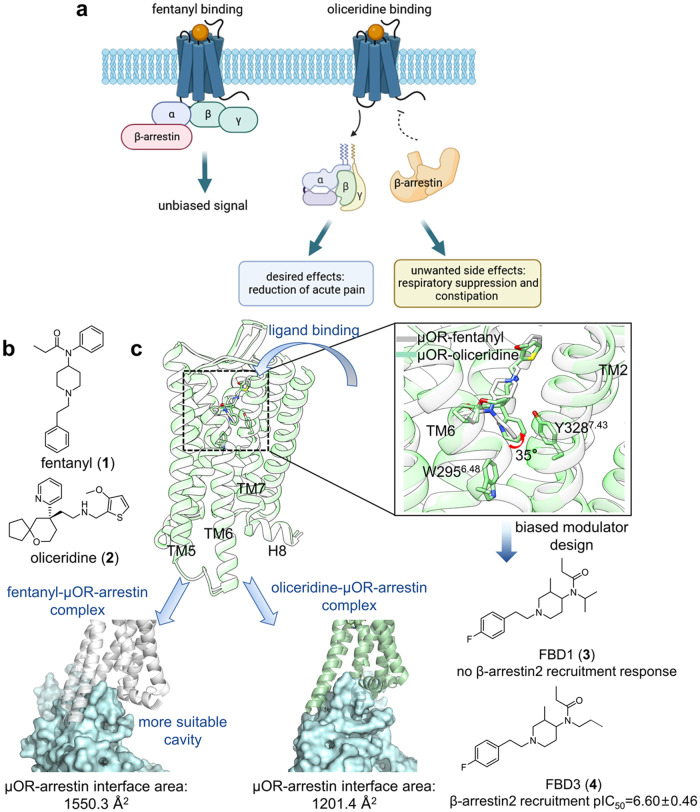
**a** A bridged general view of fentanyl and oliceridine inducing distinct pharmacological profiles. **b** 2D structure of fentanyl and oliceridine shown for clarity. **c** Superimposed views of μOR–fentanyl (gray cartoon, gray sticks; PDB: 8EF5) and μOR–oliceridine (light green cartoon, light green sticks; PDB: 8EFB) complex structure, together with the comparison of ligand binding modes and arrestin coupling interfaces, are presented. 2D structures of two designed biased modulators are also presented

With morphine and fentanyl (**1**) the most effective drugs treating acute or chronic pain,^121,122^ their common receptor μOR was revealed to be responsible for both analgesic and adverse effects.^123–125^ To attenuate side effects and broaden the therapeutic window, modulators that can abolish β-arrestin activity while maintaining relatively intact G-protein signaling are of intense pharmaceutical interest.^126–128^ Oliceridine (**2**), a partial agonist binding at the orthosteric site of μOR, was approved by the FDA in 2020 for its ability to biased signaling via the G protein pathway and thus alleviating side effects (Fig. 5a, b).^129^ Therefore, casting light on the oliceridine-μOR complex structure and the underlying mechanism of biased signaling will provide insight in developing a novel generation of analgesic drugs.

By aligning the complex structures of μOR–oliceridine and μOR–fentanyl, a well superimposed binding mode in OBP above Trp295^6.48^ was found. The only exception was that the pyridine ring of oliceridine tilts 35° toward TM2 relative to the n-aniline group of fentanyl, resulting in weaker hydrophobic interactions with TM6/7 than that with fentanyl. Based on the results of MD performed by Zhang et al.,^130^ extended interactions with TM6/7 can be inferred to have elicited inward movement of TM6 and TM7-H8 toward the TM core, shaping adaptive intracellular pocket conformation for both G-protein and β-arrestin coupling and thus leading to neutral signaling, whereas reduced interactions may have kept the intracellular end of TM6/7 relatively away from the TM core and therefore stabilize an intracellular pocket preferential for G protein binding and signaling. Two fentanyl-derived μOR agonists (**3**-**4**), which substituted the aniline group on fentanyl with n-propyl or isopropyl to reduce hydrophobicity with TM6/7, were thereupon designed as “proof-of-concept” to successfully achieve biased signaling via the G protein pathway (Fig. 5c). Different from the “trial-and-error” mode when developing biased ligand oliceridine,^131^ the comprehensive study by Zhang et al. serves as a paradigm for dissecting co-crystallized complexes to understand the molecular basis of preferential signaling mechanisms initiated from the orthosteric pocket and broadens the avenue for designing biased modulators of ORs through SBDD strategies.

#### S1PR in complex with siponimod

Sphingosine-1-phosphate receptor (S1PR), a family of class A GPCR consisting of five subtypes, S1PR1-S1PR5, modulates diverse physiological functions, including lymphocyte trafficking, vascular development, endothelial integrity, and heart rate.^132–136^ Although Fingolimod received regulatory approval from the FDA in 2010 as a first-in-class S1PR agonist,^137^ its low subtype selectivity has led to several “off-target” effects, including bradycardia and atrioventricular blockade.^138^ Therefore, a second-generation, highly subtype-selective S1PR modulator is crucially needed. Siponimod (**5**) was globally approved in 2019 for the treatment of adults with relapsing MS by selectively targeting S1PR1 and S1PR5.^139^ Insights into the mechanisms of drug recognition and receptor activation will provide a framework for understanding ligand selectivity and signal transduction in GPCRs.^140,141^

**Fig. 6:**
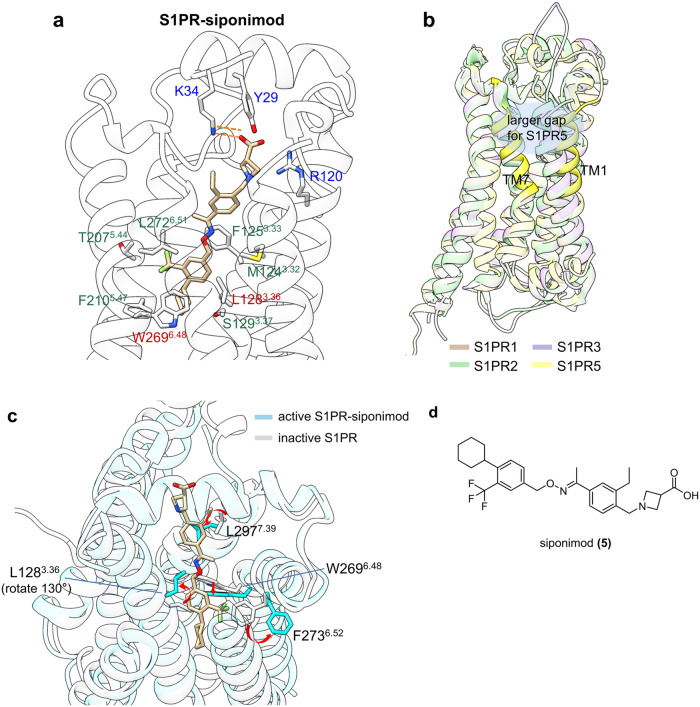
**a** Detailed binding modes of S1PR5 in complex with siponimod. Labels of the residues engaged in polar contacts with siponimod are colored in blue, with hydrogen bonds presented by orange dashes. The residues of the hydrophobic pocket that stabilizes ligand binding are marked with green labels, while residues that are critical for signal transduction are labeled in red. **b** Superimposed views of S1PR1 (orange cartoon, PDB: 7T6B), S1PR2 (light green cartoon, PDB: 7C4S), S1PR3 (light purple cartoon, PDB: 7YXA), and S1PR5 (yellow cartoon, PDB: 7TD4) GPCR structures, where TM1 and TM7 of S1PR5 are highlighted for clarity. **c** Superimposed views of active S1PR1-siponimod complex (cyan cartoon, cyan stick, PDB: 7TD4) and inactive S1PR1 structure (gray cartoon, gray stick, PDB: 3V2Y) to illustrate the “toggle switch” activation mechanism. **d** 2D structure of siponimod is shown for clarity

Yuan et al. presented the cryo-EM structures of siponimod–S1PR1–G_i_ and siponimod-S1PR5 complexes, in which the ligands exhibited an identical linear conformation across a polar module and the deep hydrophobic cavity of the orthosteric pocket (Fig. 6a).^142^ Given that members of the S1PR family display different extracellular vestibules, distinct extracellular leaflets have been reported to have contributed to diverse access channels for ligand entry and thus relate to specificity among subtypes (Fig. 6b).^143^ Moreover, further careful comparison of the siponimod-S1PR1-G_i_ complex with antagonist ML056-bound S1PR1 structure underlines the “twin toggle mechanism” during receptor activation.^144^ Upon ligand binding, Leu128^3.36^ rotates 130° away from TM5 to form a direct interaction with the hydrophobic portion of siponimod, disrupting its previous interaction with Trp269^6.48^ and triggering a synergistic downward movement of Trp269^6.48^. The dramatic displacement of the two residues can therefore loosen the interaction between TM3 and TM6, inducing a consequent outward movement of TM6 that can accommodate G protein binding (Fig. 6c, d). Similar activation mechanism involving corresponding mechanical switches can also be found in CB1 and MC4R,^145,146^ which provides valuable hints that designing ligands forming elaborate hydrophobic interaction with residue 3.36 or directly inducing reconfiguration of 3.36-6.48 may contribute to enhanced activation efficacy.

#### OX2R in complex with lemborexant

Orexin receptors are expressed throughout the central nervous system and demonstrate therapeutic potential for insomnia by regulating the sleep-wake cycle.^147–149^ The two subtypes, OX1R and OX2R, dominate the respective regulatory behaviors, with OX1R involved in gating rapid eye movement (REM) sleep and OX2R involved in gating non-REM and REM sleep.^150^ Lemborexant (**6**), an orthosteric competitive antagonist approved by the FDA in 2019, exhibits outstanding inhibitory activity against OXRs.^151,152^ However, the most important features of lemborexants lie in two aspects: (1) Why lemborexants show moderate selectivity toward OX2R over OX1R,^152^ which will facilitate the design of OX1R/OX2R-selective modulators that can be applied to REM and non-REM functionality studies? 2) What is the basis of the dynamic parameters of lemborexant that may explain the relationship between drug-induced improvement of sleep onset and a decrease in wake time after sleep?

**Fig. 7:**
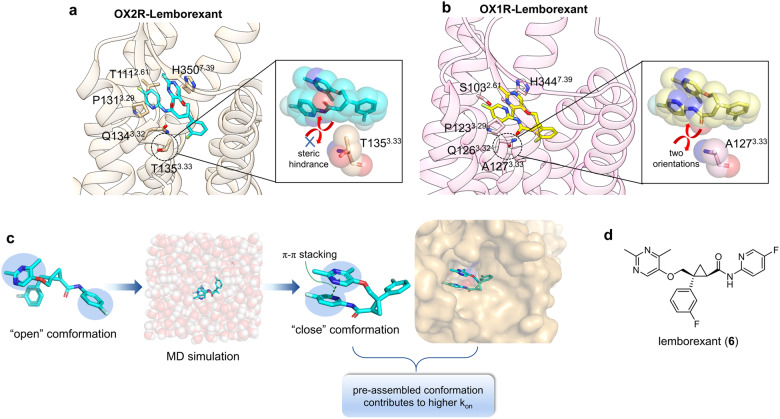
**a** Detailed binding mode of lemborexant in complex with OX2R (receptor: light orange, ligand: cyan, PDB: 7XRR), where steric hindrance of T135^3.33^ only allows one orientation of the ligand. **b** Detailed binding mode of lemborexant in complex with OX1R (receptor: light pink, ligand: yellow, PDB: 6TOT), where small side chain of A127^3.33^ accounts for two orientations of the ligand. **c** Abridged general view of employing MD simulation to predict the conformation of the ligand before receptor binding, to improve K_on_ values. **d** 2D structure of lemborexant is shown for clarity

To elucidate the mechanism of lemborexant subtype selectivity and provide guidance for anti-insomnia drug development, Asada et al. presented the crystal structure of the OX2R–lemborexant complex and compared its ligand-binding mode with that of the previously solved OX1R–lemborexant complex structure.^153^ Despite the ligand’s shared hydrogen bonds with Gln126^3.32^ of OX1R and Gln134^3.32^ of OX2R, lemborexant binds OX1R as a mixture of two orientations owing to the small side chain of Ala127^3.33^, whereas lemborexant binds OX2R in only one configuration because of the steric hindrance of Thr135^3.33^, which is inferred to be the primary cause of the difference in its affinity for OX1R and OX2R (Fig. 7a, b). In contrast, by simulating lemborexant in solution, the intramolecular stacking of two aromatic rings was observed to play a vital role in shaping the conformation of lemborexant close to the bound state before receptor binding, which explains the high k_on_ value of the ligand. In addition, the higher binding free energy of lemborexant compared to other OXR modulators may contribute to a higher k_off_ value. Collectively, these observations highlight the possibility of obtaining a high k_on_ by optimizing the conformation of free molecules via intramolecular interactions (Fig. 7c, d). By extension, separately modulating the enthalpy of molecular binding to the receptor and entropy derived from the intramolecular structure may be important strategies for designing drugs with enhanced kinetics and dynamics.

#### 5-HT_1F_ in complex with lasmiditan

The 5-HT1 receptor subtypes, including 5-HT_1A_, 5-HT_1B_, 5-HT_1D_, 5-HT_1E_, and 5-HT_1F_, are well-known class A GPCRs that respond to the endogenous neurotransmitter serotonin and have been proven to be promising targets for the treatment of migraine, depression, and schizophrenia.^154–156^ Although traditional targeted agonists have been clinically used as anti-migraine drugs for decades, side effects such as therapeutic vasoconstrictive actions owing to the non-selective activation of 5-HT_1B_ and 5-HT_1D_ remain a major hindrance.^157^ Lasmiditan (**7**), a potent and highly selective drug toward 5-HT_1F_ was approved by the FDA in 2019 because of its vasoconstrictive side effects and high-penetration properties.^158^ Elucidation of the scaffold features of lasmiditan and the mechanism of 5-HT_1F_-selective activation will provide a template for the rational design of safer anti-migraine drugs.

**Fig. 8:**
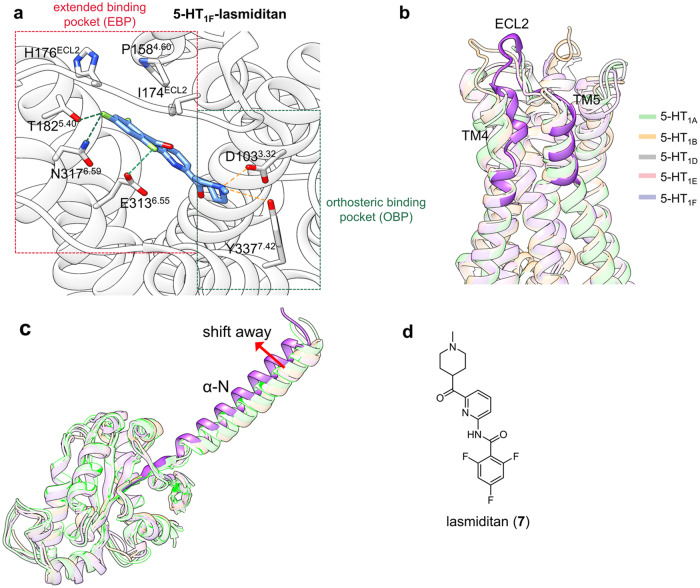
**a** Detailed binding mode of 5-HT_1F_ in complex with lasmiditan. Hydrogen bonds are presented by orange dashes, while halogen bonds are presented by green dashes. **b** Superimposed views of 5-HT_1A_ (light green cartoon, PDB: 7E2X), 5-HT_1B_ (light orange cartoon, PDB: 5V54), 5-HT_1D_ (light gray cartoon, PDB: 7E32), 5-HT_1E_ (light pink cartoon, PDB: 7E33), and 5-HT_1F_ (light purple cartoon, PDB: 7EXD). The TM4-ECL2-TM5 region of the 5-HT_1F_ receptor is highlighted for clarity. **c** The structure alignment comparison of αN helices of G protein coupling with their corresponding 5-HT receptors. αN helix of G_i_ protein coupled with 5-HT_1F_ is highlighted for clarity. **d** 2D structure of lasmiditan

Through the 5-HT_1F_-lasmiditan-G_i1_ complex solved by Huang et al., an overview of the lasmiditan-binding mode was presented.^159^ In the orthosteric binding pocket, the primary amine on the methylpiperidine group largely contributes to the stability of lasmiditan by forming a canonical charge interaction with Asp103^3.32^ of the receptor while simultaneously forming a hydrogen bond with Tyr337^7.42^. Notably, in the extended binding pocket (EBP), the trifluorobenzene group of lasmiditan forms additional hydrophobic interactions with Ile174^ECL2^ and Pro158^4.60^ and forms hydrogen bonds with residue Glu313^6.55^, Asn317^6.59^, Thr182^5.40^, and His176^ECL2^. Structural alignment of 5-HT_1F_ with other 5-HT1 receptor subtypes revealed that the TM4-TM5-ECL2 region, which is highly conserved in the other four subtypes, underwent a notable conformational change, thereby disrupting the interaction between lasmiditan and 5-HT_1A_, 5-HT_1B_, 5-HT_1D_, and 5-HT_1E_. Thus, designing ligands that accommodate EBP and form specific interactions with the TM4-TM5-ECL2 region may enable high 5-HT_1F_ selectivity (Fig. 8a, b). Activation mechanical analysis by Huang et al. revealed that lasmiditan triggers the downward movement of the toggle switch residue Trp^6.48^ and then induces conformational rearrangement of the PIF, DRY, and NPxxY motifs. Particularly, structural comparison of 5-HT_1F_-G_i_ complex and other 5-HT_1_-G_i/o_ showed that the αN of 5-HT_1F_-bound G_i_ shifts away from other 5-HT_1_ receptor-bound G_i/o_, suggesting unique G_i_ coupling and corresponding specific downstream effects (Fig. 8c). Therefore, designing modulators that interact with the toggle switch residue and optimize their blood-brain-barrier (BBB) penetration properties may yield effective and safer 5-HT_1F_ agonists.

#### GnRH1 in complex with elagolix

The representative class A GPCR, gonadotropin-releasing hormone 1 receptor (GnRH1R), once activated by its endogenous peptide activator, gonadotropin-releasing hormone (GnRH), can initiate the reproductive hormone cascade and release gonadotropins through the activation of the G_q_ protein pathway.^160–162^ With the first availability of GnRH1R non-peptidic antagonist elagolix (**8**) on the market in 2018,^163^ structural insights into the GnRH1R-elagolix complex have gained pharmaceutical interest.^164^ Additionally, unlike other class A GPCRs, GnRH1R lacks a C-terminal helix (helix 8) in the cytoplasmic region and harbors Asn^2.50^ instead of the highly conserved Asp^2.50^ present in other receptors,^165^ leaving a wide space for different microswitches along the signaling cascade within 7TMD.

**Fig. 9:**
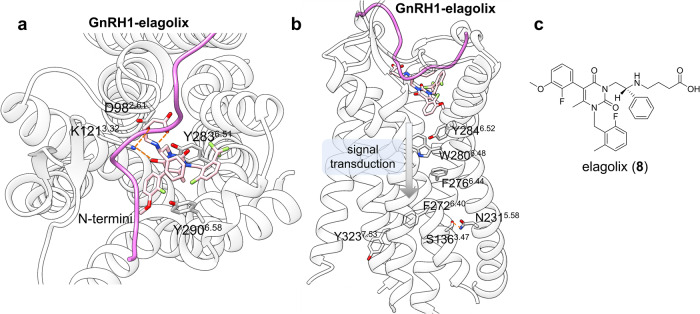
**a** Detailed binding mode of GnRH1 in complex with elagolix (receptor: light gray, ligand: light pink, PDB: 7BR3), where N-termini of GnRH1R is highlighted in a light purple to present its co-occupation with elagolix in the orthosteric pocket. **b** Overview of the special signal transduction mechanism in GnRH1R. **c** 2D structure of elagolix for clarity

The crystal structure of the GnRH1R-elagolix complex studied by Yan et al. revealed that polar network residues composed of Lys121^3.32^ and Asp98^2.61^ play critical roles in forming polar interactions with the ligand, whereas Tyr283^6.51^ and Tyr290^6.58^ are engaged in ligand recognition by contributing to hydrophobic interactions (Fig. 9a). Notably, Elagolix is located closer to TM7, resulting in an enlarged orthosteric pocket that allows N-terminal entry and co-occupation of the site. Structural alignment and IP accumulation assays showed that, unlike some GPCRs in which ligands can contact residue Trp^6.48^ directly and trigger the toggle switch, the special motif Tyr283^6.51^-Tyr284^6.52^-Trp280^6.48^-Phe276^6.44^ in TM6 was suggested to be a critical structural motif involved in mediating the propagation of signal transmission (Fig. 9b). Moreover, only 4% of class A GPCRs, including GnRH1R, have asparagine at the 5.58 position, which is implicated in a polar interaction with Ser136^3.47^ GnRH1R, thus leading to TM6 packing tightly with TM3 and TM5 in GnRH1R and exercising an antagonistic function. Collectively, these analyses highlight the distinctive features of GnRH1R in the binding of a representative antagonist and provide insights for structural biologists.

#### SFSR study utilizing structural information

While the development of crystallography over the last decade has revealed an attractive possibility of SBDD, the mainstream strategy of GPCR drug discovery remains extensive SAR study and fragment-based drug design (FBDD).^166–168^ This is partially due to the “activity-cliff” phenomenon which to some extent, undermines the profits from structural information. Nevertheless, the promising prospect still deserves expecting. Two examples are analyzed here to arouse future interest in SFSR studies utilizing structural information.

**Fig. 10:**
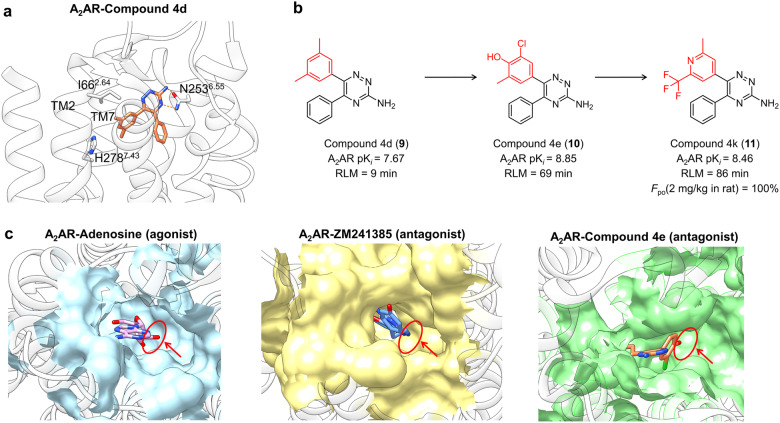
**a** Detailed binding mode of A_2_AR in complex with compound 4d (receptor: light gray, ligand: orange, PDB: 3UZA). **b** SAR study of A_2_AR antagonist. **c** Comparison of the orthosteric binding site of A_2_AR–Adenosine complex (light blue, PDB: 2YDO), A_2_AR–ZM241385 complex (light yellow, PDB: 4EIY), A_2_AR–Compound 4e complex (light green, PDB: 3UZC), the difference in cavity occupation is highlighted by red circles and arrows

The first example is the efficient discovery and optimization of A_2_AR selective antagonist 1,2,4-triazine derivative **4d** (**9**) via SBDD strategy. With Biophysical Mapping (BPM) approach and crystal structure analysis, compound **4d** was revealed to be primarily stabilized by two hydrogen bonds between the triazine core and N253^6.55^, with ring A oriented towards TM2 and TM7 (Fig. 10a). Hence, the presence of a hydrogen bond acceptor at the para position of ring A to interact with His278^7.43^, as well as the introduction of one or more flanking lipophilic substituents on the same ring to interact with Ile66^2.64^ was suggested as the focus of the SAR program. Introducing either a phenolic hydroxyl or 4-pyridyl nitrogen at the para position of ring A, and fine-tuning affinity by various combinations of small lipophilic substituents efficiently yielded compound **4k**, which proves the best balance of potency and efficacy (Fig. 10b).^169^ Further research compared the binding pockets of A_2_AR in complex with adenosine (agonist), ZM241385 (antagonist), and compound **4e** (antagonist). The hydrophobic sub-pocket in the lower chamber was observed to be occupied by the ribose ring system of adenosine analogs in agonist complexes, though was typically unoccupied when antagonists bound. The same region also allowed optimization of selectivity for A_2_AR over A_1_AR (Fig. 10c). Therefore, expanding chemotypes into this region may harvest a more efficient chemical series when designing selective and diverse functional modulators.^170^

**Fig. 11:**
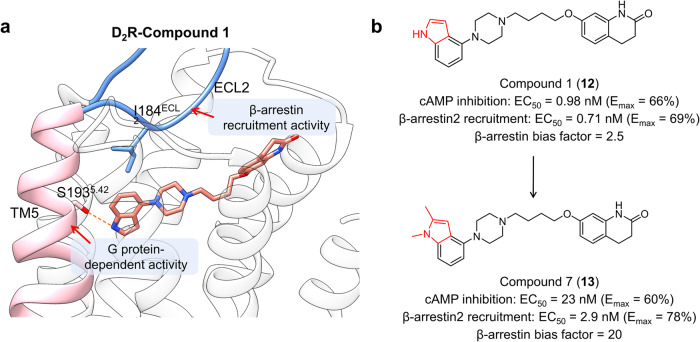
**a** Detailed binding mode of D_2_R in complex with compound 1 (**12**) (receptor: light gray, ligand: salmon, the receptor is modeled from PDB: 3PBL). TM5 of the receptor is colored in pink and ECL2 is colored in blue for clarity. **b** SAR study of β-arrestin biased agonists of D_2_R

The second paradigm entails the structure-based drug design of novel β-arrestin-biased D2R agonists commencing with aripiprazole, so as to alleviate the movement disorders associated with the adverse effects of antipsychotics.^171^ The dichlorophenylpiperazine portion of aripiprazole was first replaced with an indolepiperazine, leading to **12** that displayed comparable activity in both G_i/o_-mediated cAMP inhibition and β-arrestin2 recruitment assays. Molecular docking with a D_2_R homology model revealed that the indole NH of **12** formed a hydrogen bond with Ser5.42, which has been shown to mediate G-protein-dependent signaling in highly homologous β2 adrenergic receptors. A methyl group was thus attached to the NH of indole to fine-tune the binding conformation of **12** and thereby preclude TM5 engagement (Fig. 11a). Inspired by structural information from homologous 5-HT_2B_ receptor, where ligand interactions with hydrophobic residues on ECL2 appear to promote β-arrestin recruitment (Fig. 11a), a second methyl was introduced to position 2 of the indole ring, yielding **13** with a β-arrestin bias factor of 20 and potentially reduced side effects (Fig. 11b).^172^ To our knowledge, this is the first successful attempt at using structural information for the rational design of GPCR-biased ligands, underlining the necessity of interactive structural comparison in SFSR study.

### Delineation of GPCR structures complexed with small-molecule allosteric modulators and allosteric signaling

**Fig. 12:**
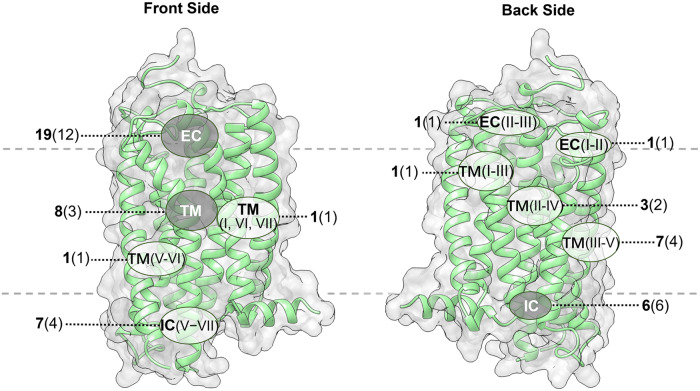
11 allosteric binding sites reported across GPCRs mapped onto representative class A GPCR CB1R. Gray pockets represent binding pockets within 7TMD, and white pockets represent binding pockets outside 7TMD. For each pocket, the number of unique ligands is indicated using boldface type, and the number of GPCRs containing the pocket is provided in parentheses. The boundary of the lipid bilayer is indicated by gray dashes

Over the past 10 years, allosteric drug discovery targeting GPCRs has witnessed significant progress in structural understanding, with the advance in knowledge of GPCR allostery.^173,174^ Till February 2024, the crystal structures of 59 allosteric small-molecule modulators bound to GPCRs have been solved, including 33 class A, 7 class B, 18 class C, and 1 class F modulators. These structures reveal that despite the intrinsic dynamic nature of GPCRs and the structural diversity among different GPCRs, only limited locations function as allosteric pockets, and the same pockets are present in GPCRs with different homologies.^175^ Even within a single receptor, more than one allosteric site has been identified. In addition, druggable allosteric hotspots spread throughout the receptor and can be divided into the following sections: extracellular vestibule, transmembrane domain, intracellular surface, outside 7TMD, and inside 7TMD domains.^174,175^ As will be discussed, allosteric binding sites in all GPCRs are currently known to be located at 11 distinct locations with some consensus,^176^ depicted in Fig. 12. In this figure, the locations of all pockets identified in different GPCRs are mapped onto the structure of an example GPCR to facilitate the comparison of these sites.

Based on the compounds’ ability to affect the stimulatory activity of orthosteric ligands, allosteric ligands can be classified into several categories, including positive allosteric modulators (PAMs), negative allosteric modulators (NAMs), allosteric modulators, and allosteric inverse agonists.^22,177^ A PAM, such as cinacalcet, targets the calcium-sensing receptor (CaSR) and potentiates the response of the receptor to its orthosteric agonist. Conversely, NAM attenuates the response of the receptor to its orthosteric agonist, mavoglurant, which targets the metabotropic glutamate receptor 5 (mGluR5).^178^ Ago allosteric modulators can activate or inhibit a receptor without an orthosteric agonist such as compound 2, which targets the glucagon-like peptide-1 receptor (GLP-1R).

#### Targeting of GPCR extracellular vestibule (outside and inside 7TMD)

**Fig. 13:**
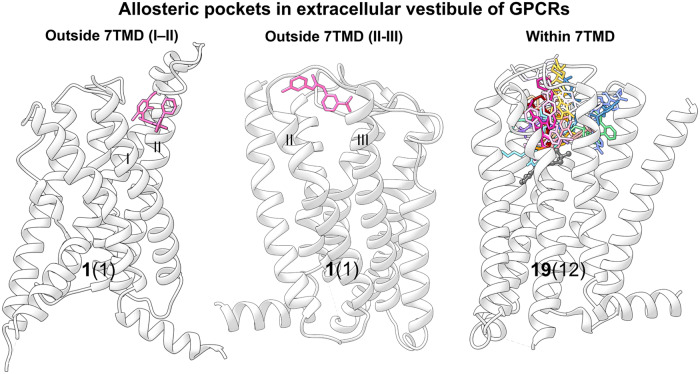
Three extracellular allosteric binding sites in GPCRs and the corresponding small-molecule allosteric modulators. Stick models of small-molecule ligands are mapped to representative members of outside 7TMD (I and II) (GLP-1R, PDB: 6VCB), outside 7TMD (II and III) (GPR101, PDB: 8W8S), and within 7TMD (M4R, PDB: 7V68) GPCRs. The position of an orthosteric ligand of M4R (shown in gray and sphere-and-stick representation) is mapped onto the overview of allosteric modulators for comparison. For each pocket, the number of unique modulators is indicated in boldface type, and the number of GPCRs containing the pocket is indicated in parentheses

After the first FDA approval of cinacalcet (a PAM of CaSR) in 2004 as a treatment for hyperparathyroidism,^179^ small-molecule allosteric modulators bound to the extracellular vestibule have developed rapidly. Till date, four of these modulators have been approved by the FDA, and one has entered clinical trials, as summarized in Table 1. Resolved crystal structures have revealed three extracellular binding sites: the pocket outside helices I and II, the pocket outside helices II and III, and the pocket inside 7TMD (Fig. 13).^180,181^ Due to their proximity to the traditional active sites of class A and B GPCRs, such allosteric modulators may exert their effects by directly altering the binding of orthosteric ligands to the receptor. As GPCRs evolved from a common ancestor, this allosteric site, found on receptors, may represent the ancestral orthosteric site.^176,182^

**Table 1:** Solved GPCR structures complexed with synthetic allosteric modulators bound to the extracellular vestibule

Structure Type	GPCR Type	GPCR	Modulator	Highest Phase	Modulator type	Number	PDB code	Allosteric site	Refs
Cryo-EM	Class B	GLP-1R	LSN3160440	Pre-clinical	PAM	(14)	6VCB	outside 7TMD (I-II)	^180^
Cryo-EM	class A	GPR101	AA-14	Pre-clinical	Allosteric agonist	(15)	8W8S	outside 7TMD (II-III)	^201^
X-ray diffraction	Class A	CCR5	maraviroc	Approved	Allosteric inverse agonist	(16)	4MBS	inside 7TMD	^181^
X-ray diffraction	Class A	PAR2	AZ8838	Pre-clinical	Allosteric antagonist	(17)	5NDD	inside 7TMD	^451^
X-ray diffraction	Class A	GPR52	c17	Pre-clinical	Allosteric agonist	(18)	6LI0	inside 7TMD	^452^
Cryo-EM	Class A	LHCGR	Org43553	Pre-clinical	Allosteric agonist	(19)	7FIH	inside 7TMD	^453^
X-ray diffraction	Class A	M2R	LY2119620	Pre-clinical	PAM	(20)	4MQT	inside 7TMD	^216^
Cryo-EM	Class A	M4R	LY2119620	Pre-clinical	PAM	(20)	7V68	inside 7TMD	^217^
Cryo-EM	Class A	M4R	compound-110	Pre-clinical	Allosteric agonist	(21)	7V6A	inside 7TMD	^217^
Cryo-EM	Class A	M4R	LY2033298	Pre-clinical	PAM	(22)	7TRP	inside 7TMD	^454^
Cryo-EM	Class A	M4R	VU0467154	Pre-clinical	PAM	(23)	7TRQ	inside 7TMD	^454^
Cryo-EM	Class A	TSHR	ML109	Pre-clinical	Allosteric agonist	(24)	7XW6	inside 7TMD	^455^
Cryo-EM	Class A	MRGPRX1	ML382	Pre-clinical	PAM	(25)	8DWG	inside 7TMD	^456^
X-ray diffraction	Class C	mGluR1	FITM	Pre-clinical	NAM	(26)	4OR2	inside 7TMD	^457^
Cryo-EM	Class C	CaSR	cinacalcet	Approved	PAM	(27)	7M3F	inside 7TMD	^230^
Cryo-EM	Class C	CaSR	evocalcet	Approved	PAM	(28)	7M3G	inside 7TMD	^230^
Cryo-EM	Class C	CaSR	NPS-2143	Pre-clinical	NAM	(29)	7DD5	inside 7TMD	^458^
Cryo-EM	Class C	CaSR	R-568	Pre-clinical	PAM	(30)	7SIL	inside 7TMD	^459^
Cryo-EM	Class C	mGluR2	JNJ-40411813	Phase 2	PAM	(31)	7E9G	inside 7TMD	^460^
X-ray diffraction	Class C	mGluR2	NAM563	Pre-clinical	NAM	(32)	7EPE	inside 7TMD	^461^
X-ray diffraction	Class C	mGluR2	NAM597	Pre-clinical	NAM	(33)	7EPF	inside 7TMD	^461^
X-ray diffraction	Class F	SMO	vismodegib	Approved	Allosteric antagonist	(34)	5L7I	inside 7TMD	^462^

**1) Outside 7TMD (TM I-II):**

**GLP-1R–LSN3160440 structure**

The glucagon-like peptide-1 receptor (GLP-1R) is a peptide hormone class B GPCR whose activation stimulates the glucose-dependent stimulation of insulin and decreases glucagon secretion.^183–185^ For such peptide receptors, allosteric pockets on GPCRs may be easier to target for small-molecule drugs than orthosteric drugs.^186^ Therefore, highly potent agonists and PAMs of GLP-1R must be developed to treat type 2 diabetes.^187–191^

**Fig. 14:**
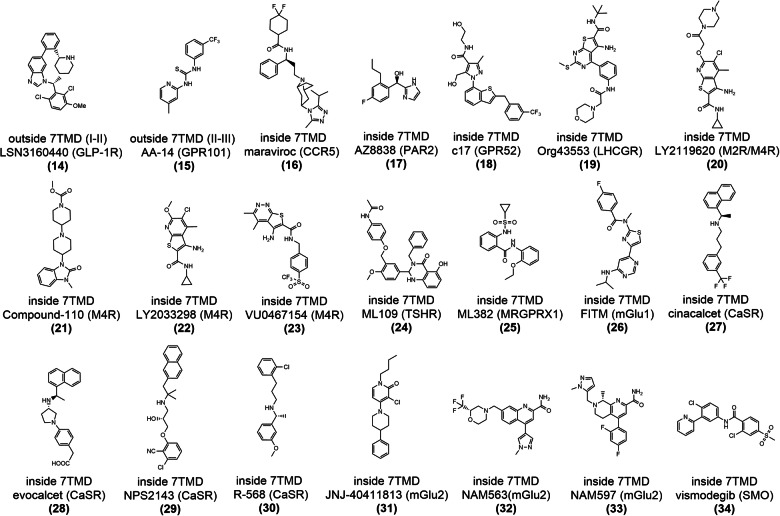
Two-dimensional (2D) chemical structures of synthetic allosteric ligands targeting the GPCR extracellular vestibule

**Fig. 15:**
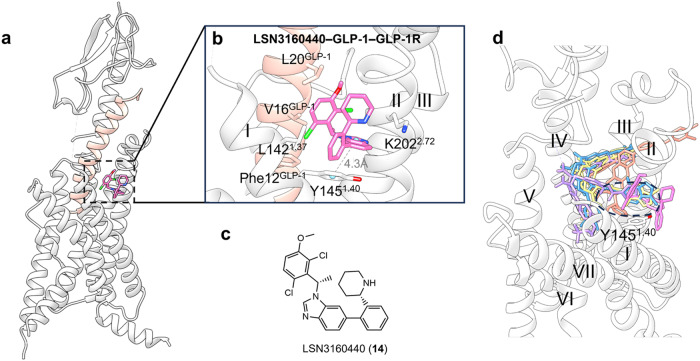
**a** Schematic representation of PAM LSN3160440 and orthosteric GLP-1 bound to GLP-1R (PDB: 6VCB). GLP-1 is indicated in pink. **b** Detailed binding modes of GLP-1R bound to LSN3160440; π–π stacking is indicated in gray dashes. **c** 2D structure of small-molecule allosteric ligand LSN3160440 presented for clarity. **d** Superposition of orthosteric small-molecule agonists Boc5 (displayed with purple sticks), TT-OAD2 (displayed with salmon sticks), LY3502970 (displayed with yellow sticks), and CHU-128 (displayed with blue sticks) to LSN3160440–GLP-1–GLP-1R structure reveals a partial overlap in the TM1-TM2 cleft. The conserved residue Tyr145^1.40^ is highlighted

LSN3160440 (**14**) (Fig. 14) is a small-molecule PAM targeted GLP-1R with an EC_50_ of 1 μM to enhance the potency and efficacy of GLP-1(9-36) becoming a full agonist.^180^ The cryo-EM structure of GLP-1R in complex with LSN3160440, the orthosteric ligand GLP-1, and the Gs protein revealed a clear depiction of the U-shaped binding mode of LSN3160440. The allosteric site is formed by residues on helices I and II in the extracellular vestibule (Fig. 15a).^180^ Within the binding pocket, the benzimidazole moiety of LSN3160440 (Fig. 15c) formed hydrophobic contacts with Leu142^1.37^ (the superscript represents the generic residue numbers of GPCRs) and engaged in aromatic interactions with Tyr145^1.40^ (Fig. 15b). Mutation and molecular dynamics (MD) simulation results also suggest that water-mediated hydrogen bonds may form between N3 of benzimidazole and Lys202^2.72^.^192,193^ Notably, LSN3160440 interacts with GLP-1 and acts as a molecular glue.^194^ The 2,6-dichloro-3-methoxyl phenyl moiety of LSN3160440 forms van der Waals interactions with Phe12^GLP-1^, Val16^GLP-1^ and Leu20^GLP-1^ simultaneously.

Several structures of orthosteric small-molecule agonists complexed with GLP-1R were resolved (Fig. 15d).^195–198^ Structural comparisons of these ligands with LSN3160440 revealed a shared region situated at the extracellular termini of the TM1-TM2 cleft, further suggesting that this is a promising area for lead optimization for both orthosteric and allosteric agonists. Within the binding site, the aromatic interactions with Tyr145^1.40^ are conserved.

**2) Outside 7TMD (TM I-II)**

**GPR101–AA-14 structure**

GPR101 is an orphan class A GPCR that is highly expressed in the nucleus accumbens and the hypothalamus and has constitutive Gs and Gq activity.^199^ GPR101 gene duplication or mutation modulates its constitutive activity, rendering GPR101 a promising target for metabolic diseases.^200^ Recent studies have identified AA-14 (**15**) (Fig. 14) as an allosteric agonist of GPR101, demonstrating robust Gs activation activity and high subtype selectivity.^201^ In vivo studies have shown that AA-14 exerts rejuvenating effects by activating GPR101 in the pituitary.

**Fig. 16:**
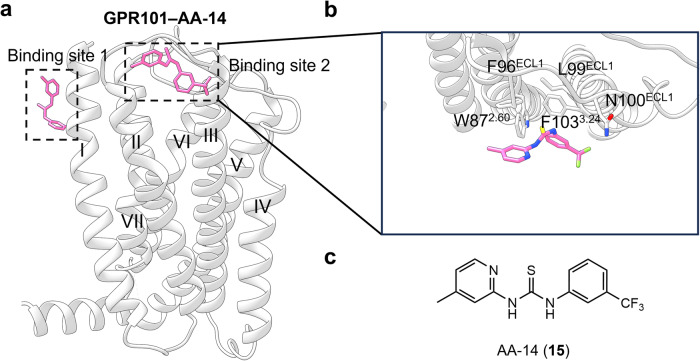
**a** Schematic representation of allosteric agonist AA-14 bound to GPR101 (PDB: 6VCB). **b** Detailed binding modes of GPR101 bound to AA-14. **c** 2D structure of small-molecule allosteric ligand AA-14 presented for clarity

The cryo-EM structure of the AA-14–GPR101–Gs complex unveils two distinct binding sites for AA-14 (Fig. 16a): one located outside 7TMD, surrounded by helices I, VI, and VII, while the other is positioned outside TM2–TM3 and ECL1.^201^ Within the extracellular allosteric site, the 3-(trifluoromethyl) phenyl group (Fig. 16c) establishes polar interactions with Asn100^ECL1^ and hydrophobic interactions with Phe103^3.24^ and Trp87^2.60^ (Fig. 16b). The 4-methyl-2-pyridinyl group packs against Phe96^ECL1^ and Leu99^ECL1^.

**3) Inside 7TMD:**

**GPR52–c17, MRGPRX1–ML382, PAR2–AZ8838, LHCGR–Org43553, M2R–LY2119620, M4R–LY2119620, M4R–compound-110, TSHR–ML109, CaSR–cinacalcet, CaSR–evocalcet, CaSR–NPS-2143, CaSR–R-568, mGluR1–FITM, SMO–vismodegib, mGluR2–JNJ-40411813, mGluR2–NAM563, mGluR2–NAM597, and CCR5–maraviroc structures**

This allosteric site is located in an extracellular pocket surrounded by a 7TM helical bundle, directly above the traditional orthosteric site of family A and B GPCRs and the cholesterol-binding site of the SMO receptor (Fig. 13).^202,203^ Until now, this allosteric site has been the most frequently targeted binding site for drug-like allosteric modulators, mainly because allosteric modulators can enter from the extracellular region, allowing ligand binding without the need to penetrate the membrane.^204^ For these receptors, the pocket in the extracellular vestibule can be partitioned into two subpockets, namely the orthosteric and allosteric pockets. The N-terminal group and ECL2 regulate the sizes of the two sub-pockets by pushing the ligand to one side,^205–207^ thereby contributing to the creation of a new ligand pocket.

As prototypical class A GPCRs, muscarinic M1–M5 acetylcholine receptors (mAChRs) are responsible for the release of acetylcholine into the brain and play fundamental roles in the central and peripheral nervous system.^208–210^ Muscarinic receptors have garnered attention as potential drug targets to treat several pathophysiological disorders including Alzheimer’s disease, schizophrenia, and drug addiction.^211–214^ LY2119620 (**20**) acts as a PAM that has activity at both the M2 and M4 receptors (Fig. 14) but is inappropriate for treatment, probably because of cross-reactivity and cardiovascular liability.^215^

**Fig. 17:**
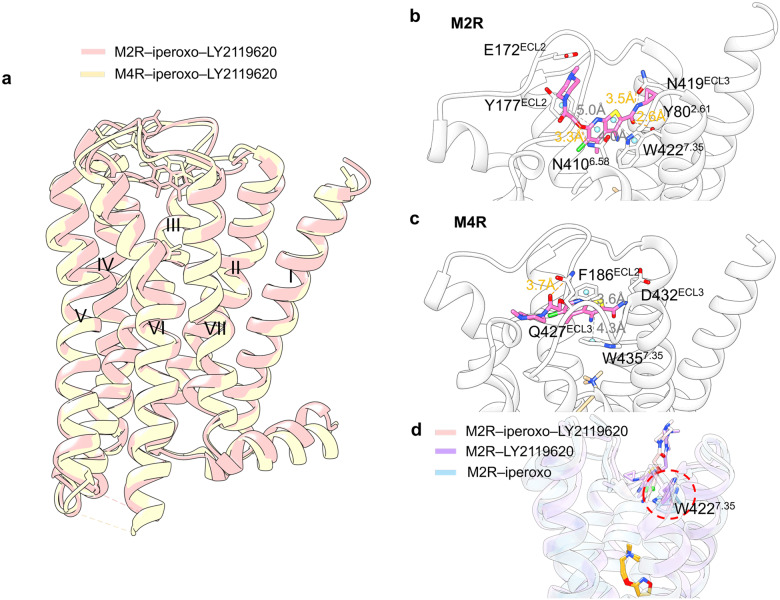
**a** Superposition of PAM LY2119620 bound to M2 receptor (pink cartoon, pink sticks; PDB: 4MQT) and M4 receptor (yellow cartoon, yellow sticks; PDB: 7V68). **b** Detailed binding modes of M2 receptor bound to LY2119620. **c** Detailed binding modes of M4 receptor bound to LY2119620. Hydrogen bonds are presented as orange dashes and π–π stacking is presented as gray dashes. **d** Superimposed views of highlighted residue Trp422^7.36^ on M2 receptor–iperoxo–LY2119620 (pink cartoon, pink sticks; PDB: 6U1N), M2 receptor–LY2119620 (purple cartoon, purple sticks; PDB: 4MQT), and M2 receptor–iperoxo (blue cartoon, blue sticks; PDB: 4MQS) structures. The orthosteric agonist iperoxo is presented in orange

The structures of the M2 and M4 receptors bound to PAM LY2119620 have been solved.^216–218^ LY2119620 demonstrated a similar binding pattern to both the M2 and M4 receptors; nonetheless, subtle differences were noted (Fig. 17a). LY2119620 binds to a spacious extracellular vestibule just above the orthosteric pocket and is segregated from the orthosteric pocket via three tyrosine residues: Tyr^3.33^, Tyr^6.51^, and Tyr^7.39^. The thienopyridine ring of LY2119620 is sandwiched by π–π stacking between Tyr177^ECL2^ and Trp422^7.35^ in M2 receptor (Fig. 17b), Phe186^ECL2^ and Trp435^7.35^ in M4 receptor (Fig. 17c). Particularly, in the M2 receptor, the residues Tyr80^2.61^, Asn410^6.58^, and Asn419^ECL3^ formed hydrogen bonds with the modulator, and Glu172^ECL2^ participated in ionic interactions with piperidine. Contrarily, in the M4 receptor, only Gln427^ECL3^ formed a hydrogen bond with the modulator.

In addition, the structures of M2 receptor–iperoxo–LY2119620 (PDB: 4MQT) and M2 receptor–LY2119620 (PDB: 6U1N) are highly similar, with Trp422^7.35^ perpendicular to the horizontal plane and forming a π–π stacking with LY2119620 (Fig. 17d). In contrast, Trp422^7.35^ of the M2 receptor–iperoxo (PDB:4MQS), exhibits a parallel conformation, suggesting that the allosteric binding site is formed predominantly in the presence of an allosteric modulator. MD simulations have revealed that LY2119620 modulates the conformation of Trp422^7.35^, causing reorientation of Tyr426^7.39^ within the orthosteric site.^219^ This reorientation may explain the observed increase in affinity for iperoxo, thereby providing insight into the underlying allosteric mechanism.^220^

For GPCRs that use other sites to bind endogenous ligands, the traditional orthosteric pocket is potentially druggable for allosteric modulators.^221^ Calcium-sensing receptors (CaSR), members of the family C GPCR, are found primarily in the parathyroid glands and kidneys to ensure strict control of calcium homeostasis.^222,223^ Elevated Ca^2+^ levels trigger the activation of CaSR, leading to the inhibition of parathyroid hormone (PTH) secretion. Thus, CaSR has become a potential target for calcimimetic drugs to treat parathyroid disorders.^224–226^ Cinacalcet (**27**) (Fig. 14), an orally active allosteric agonist of CaSR, has been used for the treatment of secondary hyperparathyroidism^227,228^ whereas calcilytic NPS-2143 (**29**) (Fig. 14) is a potent NAM-targeting CaSR that exhibits favorable in vitro and in vivo activity.^229^

**Fig. 18:**
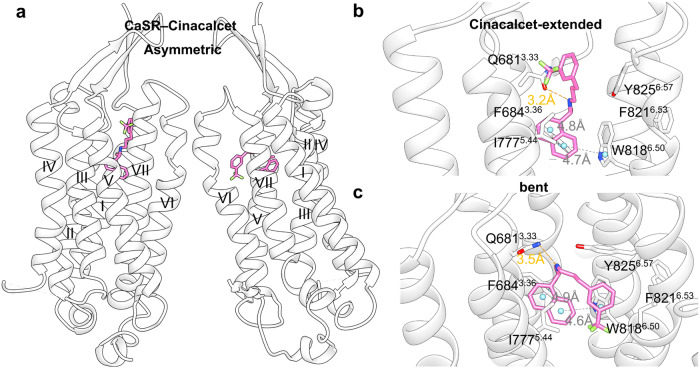
**a** Schematic representation of PAM cinacalcet bound to CaSR (PDB: 7M3F). **b** Detailed binding modes of CaSR bound to cinacalcet in extended conformations. **c** Detailed binding modes of CaSR bound to cinacalcet in bent conformations. Hydrogen bonds are presented as orange dashes and π–π stackings are presented as gray dashes

When bound to CaSR, PAM cinacalcet adopted extended and bent poses between CaSR homodimers (Fig. 18a). The naphthylethylamine moiety was bound to highly similar poses in both the extended and bent conformations (Fig. 18b, c). The naphthyl group engaged in hydrophobic interactions with Ile777^5.44^ on one side and formed edge-to-face π–π interactions with Phe684^3.36^ and Trp818^6.50^ on the other, thereby effectively securing the side chain of Trp818^6.50^ inside 7TM helical bundle. The NH group formed a hydrogen bond with Gln681^3.33^. In the extended conformation (Fig. 18b), the linker and phenyl group were parallel to TM VI, extending upward and driving Tyr825^6.57^ to orient downward. In the bent conformation (Fig. 18c), the phenyl group folded between TM V and TM VI to form a parallel-displaced π–π stacking with the naphthyl group, whereas Tyr825^6.57^ assumed a conformation perpendicular to TM VI and stabilized the ligand through a σ–π interaction.

**Fig. 19:**
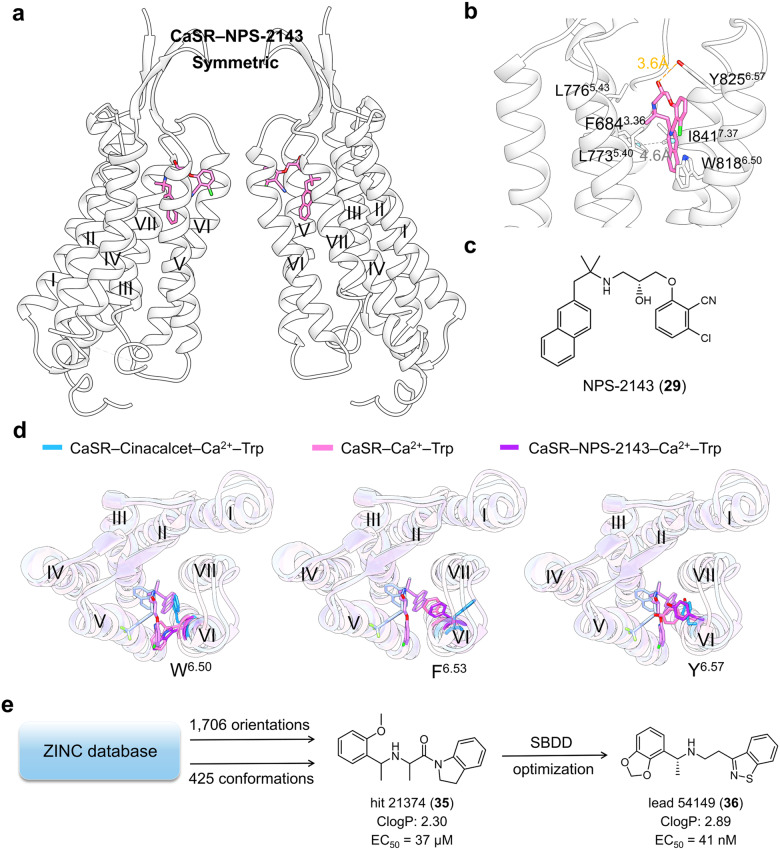
**a** Schematic representation of NAM NPS-2143 bound to CaSR (PDB: 7M3E). **b** Detailed binding modes of CaSR bound to NPS-2143. Hydrogen bond is presented as orange dashes and π–π stacking is presented as gray dashes. **c** 2D structure of small-molecule allosteric ligand NPS-2143 provided for clarity. **d** Superimposed views of highlighted residues on CaSR–Cinacalcet–Ca^2+^–Trp (blue cartoon, blue sticks; PDB: 7M3F), CaSR–Ca^2+^–Trp (pink cartoon, pink sticks; PDB: 7DD6), and CaSR–NPS-2143–Ca^2+^–Trp (purple cartoon, purple sticks; PDB: 7M3E) structures. **e** Workflow of discovery of novel CaSR PAMs utilizing structural information

In the CaSR–NPS-2143 complex, NPS-2143 exhibited the same crescent conformation as the homodimers (Fig. 19a). The naphthyl group at one end of NPS-2143 (Fig. 19c) was lined by residues Phe684^3.36^, Leu776^5.43^, Ile777^5.44^, Trp818^6.50^, and Ile841^7.37^ in the interior of the pocket (Fig. 19b). Conversely, the 3-chloro-2-cyano-phenyl ring of NPS-2143 protrudes out toward the lateral opening and forms hydrophobic contacts with Leu773^5.40^ and π–π stacking interactions with Tyr825^6.57^. A single hydrogen bond was established between the hydroxyl group and Tyr825^6.57^. Moreover, the conformation of the NAM-bound CaSR agrees well under both active (in the presence of Ca^2+^ and L-Trp) and inactive (no Ca^2+^) conditions.

Despite having highly similar binding sites, NPS-2143 and cinacalcet exhibit quite different pharmacological properties, which may be explained by the conformation of the receptor residues. The conformations of NPS-2143-bound and Ca^2+^-bound CaSR were similar.^230^ Nevertheless, cinacalcet binding induced significant conformational changes in Trp818^6.50^, Phe821^6.53^, and Tyr825^6.57^ within the allosteric pocket (Fig. 19d). Trp818^6.50^ rotates inwardly from a vertical to a horizontal conformation, forming extensive π–π interactions with cinacalcet. Phe821^6.53^ underwent an outward shift and was inserted into a crevice between TM6 and TM7 facing the dimer interfacial area. Simultaneously, Tyr825^6.57^ flips down, driven by structural conflicts in the extended conformation of cinacalcet. In summary, cinacalcet induces a bent conformation of TM6 and stabilizes the homodimer interface, thereby contributing to receptor activation.^231^ Contrarily, NPS-2143 decreased agonist efficacy by enhancing TM VI helicity, which spatially hindered receptor activation.

Based on the special binding conformations of cinacalcet, Liu et al. conducted a virtual screening of 1.2 billion compounds to discover novel PAMs with potentially novel pharmacology. To respectively mimic the “extended” and “bent” conformation, extensive orientations and conformations of library molecules were sampled, which gave 682 trillion configurations overall and finally achieved a 3.8% and 13.6% hit rate. The hits were then optimized to a pharmacologically potent lead (**36**) via synergistic application of structural information, fragment hybridization, and stereochemistry separation (Fig. 19e).^232^ Such practice serves as a paradigm for its elaborate utility of solved GPCR structures and conformation sampling strategy and is generalizable in the discovery of CaSR NAMs and other allosteric modulators.

#### Targeting of GPCR transmembrane domain (outside 7TMD)

**Fig. 20:**
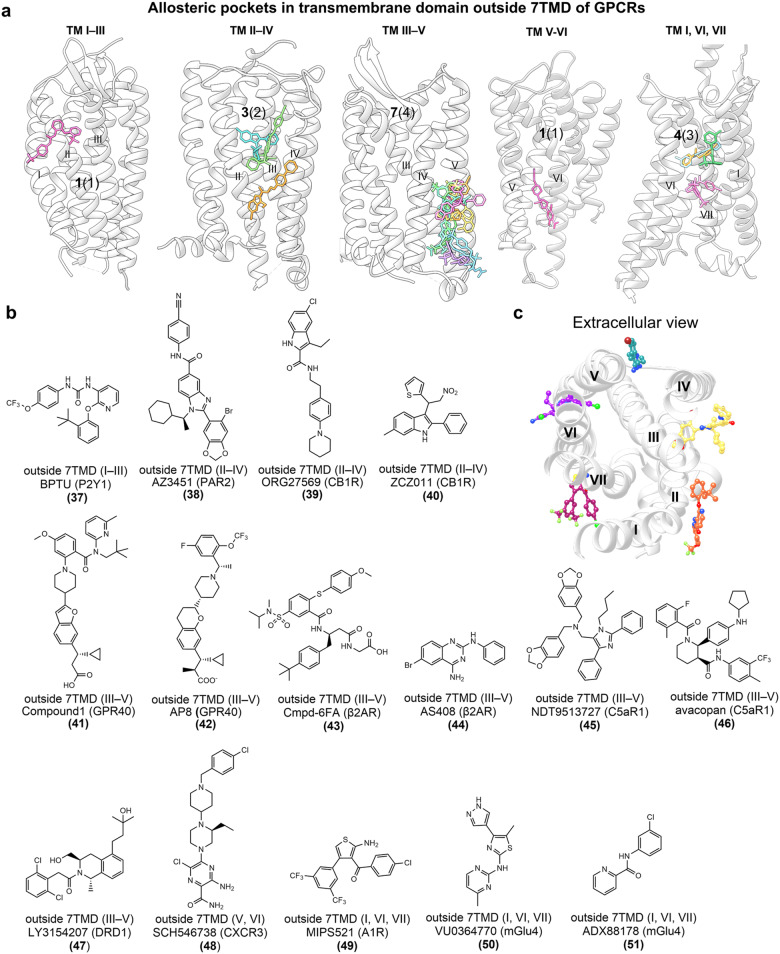
**a** Five allosteric binding sites in the transmembrane domain outside 7TMD of GPCRs and the corresponding small-molecule allosteric modulators. Stick models of small-molecule ligands are mapped to representative members of outside 7TMD (I–III) (P2Y_1_, PDB: 4XNV), outside 7TMD (II–IV) (CB1R, PDB: 6KQI), outside 7TMD (III and V) (C5aR1, PDB: 6C1R), outside 7TMD (V and VI) (CXCR3, PDB: 8HNN), and outside 7TMD (I, VI, and VII) (A1R, PDB: 7LD3) GPCRs. For each pocket, the number of unique modulators is indicated in boldface type, and the number of GPCRs containing the pocket is provided in parentheses. **b** 2D chemical structures of synthetic small-molecule allosteric ligands targeting the transmembrane domain outside 7TMD of GPCRs. **c** Extracellular view of the five allosteric sites

As shown by their structures, GPCRs utilize the domain outside 7TMD at the lipid interface to bind allosteric modulators. Till date, five different binding sites outside 7TMD in the transmembrane domain have been defined by their crystal structures (Table 2): the pocket outside helices I–III, the pocket outside helices II–IV, the pocket outside helices III–V, the pocket outside helices V–VI, and the pocket outside helices I, VI, and VII (Fig. 20a, c). Allosteric modulator binding to these regions targets class A GPCRs. These sites are typically shallow and not as well surrounded by the 7TM helical bundle as the traditional orthosteric sites. Polar functional groups are commonly found in allosteric modulators at these sites where they anchor themselves to the pocket. Thus, hydrogen atom donor or acceptor groups exposed between the receptor and lipid bilayer are more likely to mediate the binding of such allosteric ligands. These modulators are also required to preserve their overall hydrophobic character to enter the transmembrane domain. Allosteric modulators bound to the transmembrane domain outside 7TMD appear to regulate receptor signal transduction from outside the 7TM helices in a manner that stabilizes inactive or active interaction networks or impedes or facilitates the interhelical motions required for receptor activation.^233–235^

**Table 2:** Solved GPCR structures complexed with synthetic allosteric modulators bound to the transmembrane domain outside 7TMD

Structure Type	GPCR type	GPCR	Modulator	Highest Phase	Modulator type	Number	PDB code	Allosteric site	Refs
X-ray diffraction	class A	P2Y_1_	BPTU	Pre-clinical	Allosteric antagonist	(37)	4XNV	outside 7TMD (I–III)	^242^
X-ray diffraction	class A	PAR2	AZ3451	Pre-clinical	Allosteric antagonist	(38)	5NDZ	outside 7TMD (II–IV)	^451^
X-ray diffraction	class A	CB1R	ORG27569	Pre-clinical	NAM	(39)	6KQI	outside 7TMD (II–IV)	^257^
X-ray diffraction	class A	CB1R	ZCZ011	Pre-clinical	PAM	(40)	7FEE	outside 7TMD (II–IV)	^261^
X-ray diffraction	class A	GPR40	compound 1	Pre-clinical	Allosteric agonist	(41)	5KW2	outside 7TMD (III–V)	^463^
X-ray diffraction	class A	GPR40	AP8	Pre-clinical	AgoPAM	(42)	5TZY	outside 7TMD (III–V)	^464^
X-ray diffraction	class A	β2AR	Cmpd-6FA	Pre-clinical	PAM	(43)	6N48	outside 7TMD (III–V)	^269^
X-ray diffraction	class A	β2AR	AS408	Pre-clinical	NAM	(44)	6OBA	outside 7TMD (III–V)	^465^
X-ray diffraction	class A	C5aR1	NDT9513727	Pre-clinical	Allosteric inverse agonist	(45)	5O9H	outside 7TMD (III–V)	^466^
X-ray diffraction	class A	C5aR1	avacopan	Approved	Allosteric antagonist	(46)	6C1R	outside 7TMD (III–V)	^467^
Cryo-EM	class A	DRD1	LY3154207	Phase 2	PAM	(47)	7CKZ	outside 7TMD (III–V)	^277^
Cryo-EM	class A	CXCR3	SCH546738	Pre-clinical	Allosteric antagonist	(48)	8HNN	outside 7TMD (V–VI)	^299^
Cryo-EM	class A	A1R	MIPS521	Pre-clinical	PAM	(49)	7LD3	outside 7TMD (I, VI, VII)	^305^
Cryo-EM	class A	GPR101	AA-14	Pre-clinical	Allosteric agonist	(15)	8W8S	outside 7TMD (I, VI, VII)	^201^
Cryo-EM	class C	mGlu4	VU0364770	Pre-clinical	PAM	(50)	8JD5	outside 7TMD (I, VI, VII)	^468^
Cryo-EM	class C	mGlu4	ADX88178	Pre-clinical	PAM	(51)	8JD6	outside 7TMD (I, VI, VII)	^468^

**1) TM I–III:**

**P2Y**_**1**_ **–BPTU Structure**

To the best of our knowledge, one small molecule allosterically targets this relatively shallow pocket. Because of the flat TM helical bundle surface and relatively narrow cavity of the binding pocket, rational drug design in this area may be challenging.

In this instance, the protein target was the P2Y_1_ purinergic receptor. Agonists induce the activation of the P2Y_1_ receptor, leading to the potentiation of platelet aggregation that triggers platelet secretion;^236,237^ thus, antagonists targeting the P2Y_1_ receptor offer a prospective approach to treat thrombosis.^238,239^ BPTU (**37**) (Fig. 20b), a P2Y_1_ antagonist, has been gaining attention as an antithrombotic treatment and is the first allosteric GPCR modulator located outside the helical bundle.^240^ BPTU blocks the P2Y_1_-induced platelet aggregation with nanomolar potency and presents good selectivity for P2Y_1_ receptor and highly homologous P2Y_12_ receptor (P2Y_1_*K*_i_ = 75 nM, P2Y_12_*K*_i_ > 70 μM).^241^

**Fig. 21:**
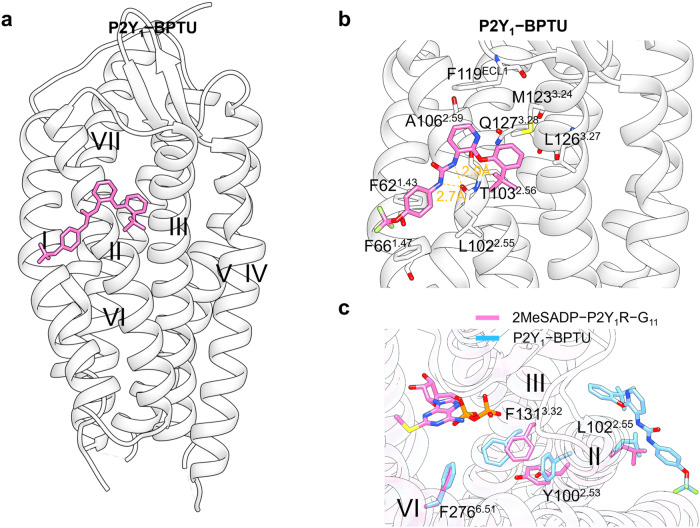
**a** Schematic representation of the allosteric antagonist BPTU bound to P2Y_1_ receptor (PDB code 4XNV). **b** Detailed binding modes of P2Y_1_ receptor bound to BPTU. Hydrogen bonds are presented as orange dashes. **c** Superimposed views of the highlighted residues on 2MeSADP − P2Y_1_R − G_11_ (pink cartoon, pink sticks; PDB: 7XXH) and P2Y_1_R − BPTU structures (blue cartoon, blue sticks; PDB: 4XNV)

The binary complex structure of P2Y_1_ − BPTU was determined and showed that the BPTU binding pocket consists mainly of residues in helices I − III (Fig. 21a).^242^ Notably, two crucial hydrogen bonds were formed between the two NH moieties of the urea group of BPTU and the main-chain carboxyl group of Leu102^2.55^ (Fig. 21b). In terms of hydrophobic interactions, the BPTU pyridyl group makes contact with the residues Ala106^2.59^ and Phe119^ECL1^. Hydrophobic interactions of the tert-butyl phenyl group occur within a distinct subpocket shaped by helices II and III, including residues Leu102^2.55^, Thr103^2.56^, Met123^3.24^, Leu126^3.27^, and Gln127^3.28^. On the opposite side of the ligand, the trifluoromethoxyphenyl group participated in hydrophobic interactions with Phe62^1.43^ and Phe66^1.47^.

This case suggests an effective shape-complementary mechanism for allosteric ligands: bulges on 7TM helices can be utilized as anchors for fixation. In this instance, Pro105^2.58^ serves as the corresponding anchor, which is conserved in 74% of non-olfactory class A GPCRs.^243^ A comparison between the P2Y_1_ receptor bound to the agonist 2MeSADP and the allosteric antagonist BPTU revealed that the BPTU induces a 1.4 Å shift in Tyr100^2.53^ toward TM3 (Fig. 21c), preventing the conformational change of Phe131^3.32^.^244^ Thus, Phe131^3.32^ interacts with Phe276^6.51^ and limits the TM6 transition, which is required for the activation of class A GPCRs.^245^ In addition, MD simulations have demonstrated that the binding of BPTU stabilizes the helical bundle, leading to an increase in lipid order.^246^ This, in turn, stabilizes the ionic lock formed between Lys46^1.46^ and Arg195^ECL2^ in the inactive receptor.

**2) TM II−IV:**

**CB1R−ORG27569, CB1R** **−** **ZCZ011, and PAR2** **−** **AZ3451 Structures**

Cannabinoid receptors, which consist of two subtypes, CB1R and CB2R, are activated by neurotransmitter endocannabinoids and play key modulatory roles in synaptic transmission.^247,248^ Among them is the most abundant GPCR in the human brain is CB1R.^249^ Because of its widespread distribution and regulatory roles in various physiological functions, CB1R is considered an important target for the treatment of various central nervous system (CNS) disorders.^250–253^

**Fig. 22:**
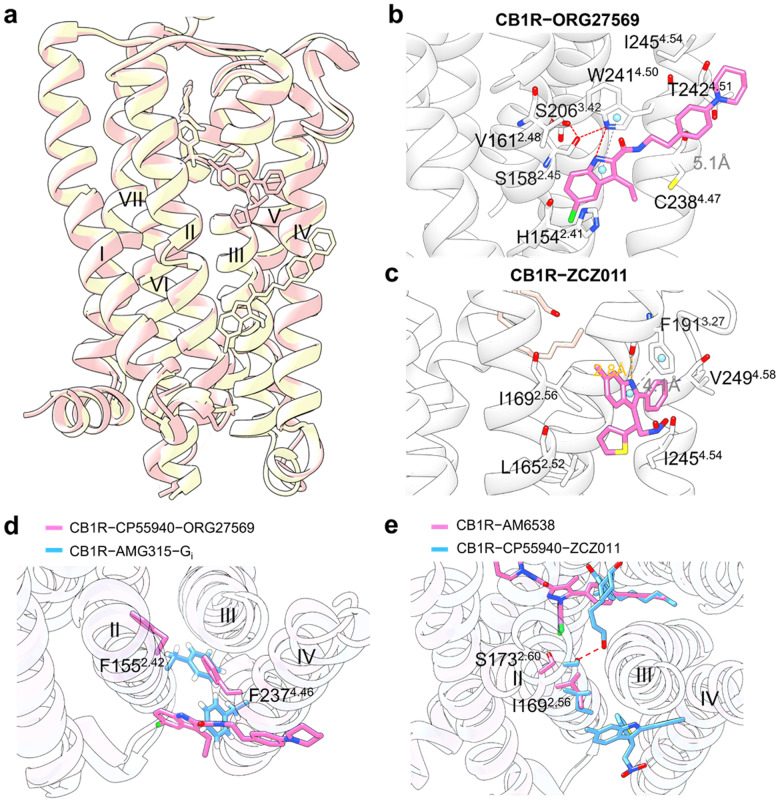
**a** Superposition of the cocrystal structures of NAM ORG27569 (yellow, PDB: 6KQI) and PAM ZCZ011 (pink, PDB: 7FEE) with orthosteric ligand-bound CB1R. **b** Detailed binding modes of CB1R binding to ORG27569. **c** Detailed binding modes of CB1R binding to ZCZ011. Hydrogen bond is presented as orange dashes, and π–π stacking is presented as gray dashes. **d** Superimposed views of highlighted residues on CB1R–CP55940–ORG27569 (pink cartoon, pink sticks; PDB: 6KQI) and CB1R–AMG315–G_i_ structures (blue cartoon, blue sticks; PDB: 8GHV). **e** Superimposed views of highlighted residues on CB1R–AM6538 (pink cartoon, pink sticks; PDB: 5TGZ) and CB1R-CP55940–ZCZ011 structures (blue cartoon, blue sticks; PDB: 7FEE)

ORG27569 (**39**) (Fig. 20b) is the first and most comprehensively studied NAM of CB1R.^254,255^ However, when tested in vivo, Org27569 was not sufficiently effective in modulating the effects of orthosteric cannabinoids.^256^ The co-crystal structure of CB1R and NAM ORG27569, together with its orthosteric CP55940 agonist, has been solved.^257^ ORG27569 occupied an allosteric site outside helices II and IV in the inner lobe of the phospholipid bilayer (Fig. 22a).^258^ Specifically, the chloro-indole ring of ORG27569 establishes a key aromatic interaction with the indole group of Trp241^4.50^ and buries in a hydrophobic pocket surrounded by His154^2.41^ and Val161^2.48^. Cys238^4.47^, Trp241^4.50^, Thr242^4.51^, and Ile245^4.54^ supply hydrophobic interactions for the amide-linked piperidinylphenyl chain (Fig. 22b).

The recently developed ZCZ011 (Fig. 20b) is also an indole derivative that exerts PAM and partial agonist effects in vivo assays.^259,260^ ZCZ011 showed good shape complementarity with the pocket outside 7TMD comprising helices II−IV (Fig. 22a). The indole group in ZCZ011 is anchored by Phe191^3.27^ and forms a hydrogen bond with the main chain of Phe191^3.27^ while forming π−π stacking interactions with the side chain. (Fig. 22c).^261^ In addition, the indole group interacted with Leu165^2.52^, Ile169^2.56^, Ile245^4.54^, and Val249^4.58^ from TM II and TM IV, respectively.

NAM ORG27569 and AZ3451 (**38**), as well as PAM ZCZ011, bind to the same TM II−IV surface, however, exert opposite allosteric effects.^262^ Unlike traditional NAMs, ORG27569 enhances the affinity of the agonist though reduces the activity of G_i_ turnover.^263^ Structurally, the inactivating efficacy of ORG27569 acts by stabilizing the “activation switch” formed by Phe155^2.42^ and Phe237^4.46^ in CB1R (Fig. 22d).^264,265^ Furthermore, a hydrogen bond was formed between ORG27569 and Trp241^4.50^, which, together with the hydrogen bond formed between Trp241^4.50^, Ser158^2.45^, and Ser206^3.42^ (Fig. 22b), created a polar network that contributed to maintaining the inactive conformation.^266^ However, the precise mechanism through which ORG27569 augments agonist affinity remains to be elucidated. Upon comparing the CB1 structures bound to the PAM ZCZ011 and the antagonist AM6538, a notable shift of Ile169^2.56^ in TM2 towards TM3 was observed. This shift results in the contraction of the receptor“s active site (Fig. 22e), and is believed to be associated with activation.^267,268^ Additionally, Ser173^2.60^ undergoes notable inward movement and forms a hydrogen bond with CP55940, thereby stabilizing the agonist binding.

**3) TM III–V:**

**GPR40–compound 1, GPR40–AP8, β2AR–AS408, β2AR–Cmpd-6FA, C5aR1–NDT9513727, C5aR1–avacopan, and DRD1–LY3154207 structures**

A deep pocket is present outside the transmembrane helices III–V among GPCRs, which allows for the presence of a population of allosteric regulators bound to this pocket. In this case, allosteric agonists and PAMs (i.e., compound 1 (**41**), AP8 (**42**), Cmpd-6FA (**43**), and LY3154207 (**47**)) localize to regions near ICL2 and stabilize the ICL2 α helix through direct interactions, facilitating the inward movement of Pro^ICL2^. This movement leads to a ~3° inward displacement of TM III, which in turn determines the outward shift of TM V together with TM VI, which is a hallmark of GPCR activation.^269,270^ Thus, the binding of allosteric modulators increases the proportion of receptors that adopt active conformations, thereby increasing their affinity for agonists. Contrarily, allosteric antagonists and NAMs (i.e., AS408 (**44**), NDT9513727 (**45**), and avacopan (**46**)) bind to regions far from ICL2. As these ligands are bound close to the proline kink of TM V, the receptor-ligand interactions collectively inhibit the interhelical movements and rotations within TM III, TM IV, and TM V, which are required for receptor activation.^271^

**Fig. 23:**
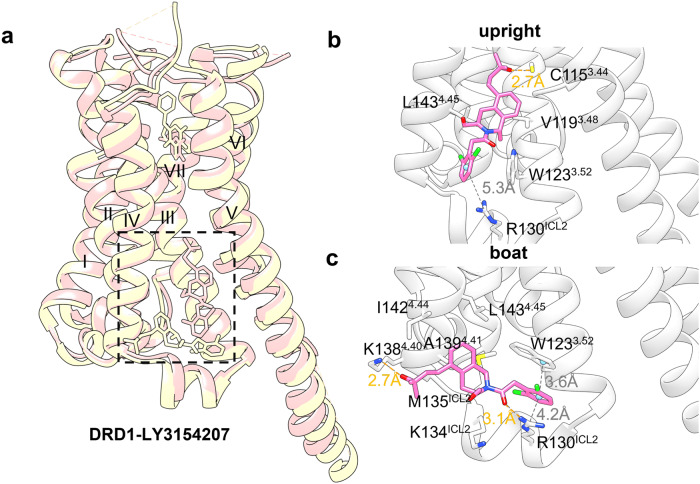
**a** Superposition of the cocrystal structures of PAM LY3154207 bound to dopamine D1 receptor in upright (pink, PDB: 7CKZ) and boat (yellow, PDB: 7LJC and 7X2F) conformations. **b** Detailed binding modes of dopamine D1 receptor binding to LY3154207 in upright conformations. c Detailed binding modes of dopamine D1 receptor binding to LY3154207 in boat conformations. Hydrogen bonds are presented as orange dashes; π–π stacking and π–cation stacking interactions are presented as gray dashes

The dopamine D1 receptor is a prototypical example. Dopamine functions as an essential catecholamine neurotransmitter that signals via the dopamine D1 to D5 receptors.^272,273^ The dopamine D1 receptor (DRD1) regulates neuronal growth, memory, and learning in the central nervous system.^274,275^ LY3154207 (Fig. 20b) is a selective PAM of the dopamine D1 receptor that improved motor symptoms associated with Lewy body dementia in a 2022 phase 2 clinical trial.^276^ Two distinct binding modes of LY3154207 to DRD1 have been reported, and structural comparisons have demonstrated upright and boat conformations (Fig. 23a).^277–280^ In both binding modes, LY3154207 was localized at the receptor–lipid bilayer interface surrounded by TM III, TM IV, and ICL2.

In the upright conformation, the tertiary alcohol group of LY3154207 extends toward TM III, establishing a single hydrogen bond with Cys115^3.44^ (Fig. 23b). The central tetrahydroisoquinoline ring of LY3154207 engages in extensive hydrophobic interactions with Val119^3.48^, Trp123^3.52^, and Leu143^4.45^. Additionally, the dichlorophenyl group forms a π-cation interaction with the side chain of Arg130^ICL2^. In the boat conformation, the dichlorophenyl group participates in π–cation interactions with the side chains of Arg130^ICL2^, and interacts sandwich-like π–π stacking with Trp123^3.52^ (Fig. 23c). The tetrahydroisoquinoline ring of LY3154207 established hydrophobic interactions with neighboring residues, including Met135^ICL2^, Ala139^4.41^, Ile142^4.44^, Leu143^4.45^, and the alkyl chain of Lys134^ICL2^. In addition, two hydrogen bonds were observed between LY3154207 and the polar residues Arg130^ICL2^ and Lys138^4.40^.

Superposition of the D1R–LY3154207–dopamine and D1R–dopamine structures showed near-identical binding positions for dopamine and the surrounding residues. Instead, LY3154207 interacted with Arg130^ICL2^ and Lys134^ICL2^ and stabilized ICL2, which interacts directly with G-proteins, thereby increasing the population of D1R adopting active conformations (Fig. 23b, c).^279^

Another example of an allosteric modulator that binds outside 7TMD formed by helices III–V is the allosteric antagonist avacopan of C5a receptor 1. Human C5a receptor 1 (C5aR1), which binds to the pro-inflammatory mediator C5a, is primarily expressed on the surfaces of various immune cells, such as neutrophils, eosinophils, and dendritic cells.^281,282^ Overactivation of the C5aR1-C5a axis will cause uncontrolled inflammation;^283^ thus, C5aR1 antagonists are ideal candidates for treating various inflammatory conditions, including sepsis COVID-19, etc.^284–286^

**Fig. 24:**
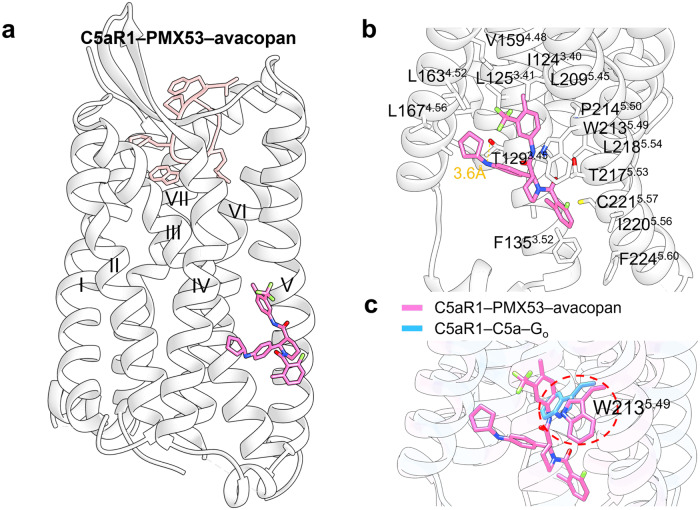
**a** Schematic representation of the allosteric antagonist avacopan bound to C5a receptor 1 (PDB: 6C1R). **b** Detailed binding modes of C5a receptor 1 bound to avacopan. Hydrogen bond is presented as orange dashes. **c** Superimposed views of highlighted residue Trp213^5.49^ on C5aR1–PMX53–avacopan (pink cartoon, pink sticks; PDB: 6C1R) and C5aR1–C5a–G_o_ (blue cartoon, blue sticks; PDB: 8IA2) structures

Avacopan (Fig. 20b) is an orally administered allosteric antagonist of C5aR1 approved by the FDA in 2021 to treat severe autoantibody (ANCA)-ANCA-associated vasculitis (granulomatosis with polyangiitis and microscopic polyangiitis).^287,288^ Pharmacological studies have indicated the ability of avacopan for biased inhibition of β-arrestin coupling.^289–291^ The co-crystal structure of C5aR1 was reported with avacopan, highlighting the binding site outside 7TMD between helices III and V (Fig. 24a). The cyclopentane group of avacopan extended into the crevice between helices III and IV and occupied the hydrophobic pocket consisting of Leu125^3.41^, Val159^4.48^, Leu163^4.52^, and Leu167^4.56^ (Fig. 24b). The o-methyltrifluoromethylbenzene group exhibited hydrophobic and aromatic interactions mediated by residues Ile124^3.40^, Leu125^3.41^, Leu209^5.45^, Trp213^5.49^, Pro214^5.50^, and Leu218^5.45^ in the binding cleft between helices III and V. The m-methylfluorobenzene group lies deeper and forms non-polar interactions with residues Phe135^3.52^, Ile220^5.56^, Cys221^5.57^, and Phe224^5.60^ in C5aR1. Only one hydrogen bond was observed between the carbonyl substituent of the amide bond of avacopan and Trp213^5.49^. Additionally, there is a water-mediated polar interaction between avacopan and Thr217^5.53^.

In the inactive C5aR1 structure, Trp213^5.49^ undergoes a conformational transition to accommodate avacopan binding, in contrast to the active C5aR1 structure (Fig. 24c). Avacopan may stabilize the conformation of the residues Ile124^3.40^, Pro214^5.50^, and Phe251^6.44^ in their inactive states through direct hydrophobic interactions (Fig. 24b). This stabilization, in turn, hinders conformational changes in transmembrane helices TM5 and TM6, which are necessary for receptor activation.^292,293^

**4) TM V–VI:**

**CXCR3–SCH546738 structure**

**Fig. 25:**
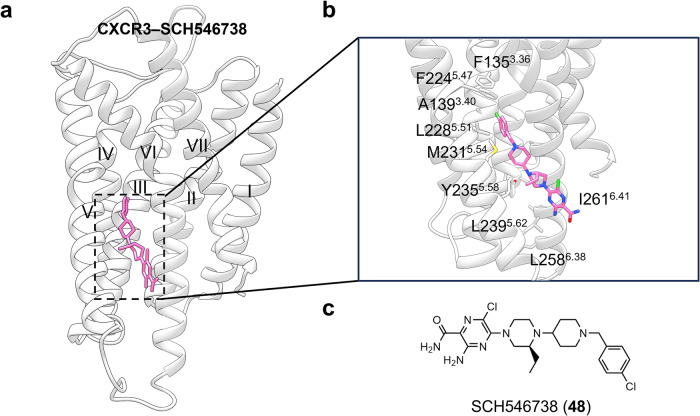
**a** Schematic representation of the allosteric antagonist SCH546738 bound to CXCR3 (PDB code 8HNN). **b** Detailed binding modes of CXCR3 binding to SCH546738. **c** 2D structure of small-molecule allosteric ligand SCH546738 provided for clarity

C-X-C chemokine receptor type 3 (CXCR3), a class A GPCR, is highly expressed on effector T cells and is activated by chemokines CXCL9, CXCL10 and CXCL11.^294^ Due to the critical role of CXCR3 in type 1 immunity, agonists and antagonists targeting CXCR3 have been synthesized to treat infection, autoimmune diseases, allograft rejection and cancers.^295–297^ Among these, SCH546738 (**48**) (Fig. 25c) has shown remarkable efficacy in several preclinical trials by effectively inhibiting the activation of T cell chemotaxis with an affinity of 0.4 nM.^298^

In the CXCR3–SCH546738 structure, SCH546738 is trapped in a narrow hydrophobic pocket surrounded by TM3, TM5 and TM6 (Fig. 25a).^299^ The head of SCH546738 is surrounded by a hydrophobic pocket formed by residues Phe135^3.36^, Ala139^3.40^, Phe224^5.47^, Leu228^5.51^, Met231^5.54^, Ile261^6.41^, Ala273^6.53^ (Fig. 25b). The tail of SCH546738 stretches out to the lipid bilayer and interacts with Tyr235^5.58^, Leu239^5.62^ and Leu258^6.38^. Given the unique allosteric site of SCH546738, the interposition of SCH546738 may weaken the repacking between TM5-TM6, maintaining the receptor in an inactive state.

**5) TM I, VI, VII:**

**A1R–MIPS521, GPR101–AA-14, mGlu4–VU0364770, and mGlu4–ADX88178 Structures**

The adenosine A1 receptor (A1R), a subtype of the adenosine receptor,^300^ has been a highly pursued non-opioid analgesic target for the treat chronic pain.^301–304^ Nonetheless, no selective clinically approved A1R agonists or antagonists are currently available.

**Fig. 26:**
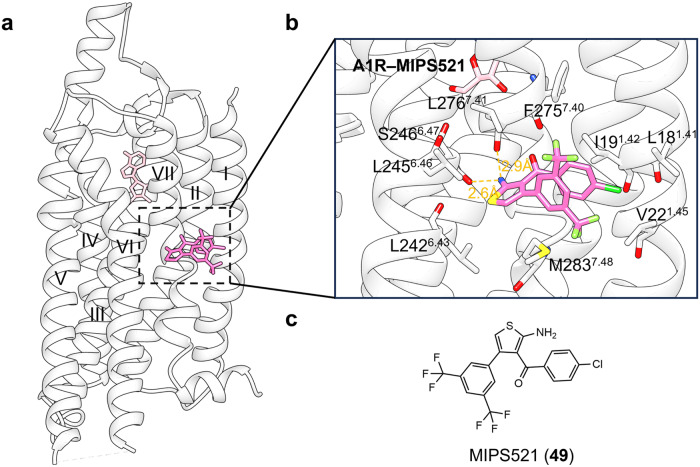
**a** Schematic representation of PAM MIPS521 bound to A1R (PDB code 7LD3). **b** Detailed binding modes of A1R binding to MIPS521. Hydrogen bonds are presented as orange dashes. **c** 2D structure of small-molecule allosteric ligand MIPS521 provided for clarity

MIPS521 (**49**) (Fig. 20b), a PAM of A1R, suppressed spinal nociceptive signaling and displayed an analgesic effect in a rat model with a pEC50 of 6.9 ± 0.4.^305^ Structural analysis revealed that MIPS521 binds outside 7TMD surrounded by helices I, VI, and VII (Fig. 26a). Residues Leu18^1.41^, Ile19^1.42^, Val22^1.45^, Leu242^6.43^, Leu245^6.46^, Ser246^6.47^, Phe275^7.40^, Leu276^7.41^, and Met283^7.48^ formed shallow hydrophobic pockets at the allosteric site (Fig. 26b). The amino group of MIPS521 (Fig. 26c) was hydrogen-bonded to the main-chain carbonyl groups in Ser246^6.47^ and Leu276^7.41^. Comparing the ADO–A1R–G_i2_ and MIPS521–ADO–A1R–G_i2_ structures showed that MIPS521 has minimal impact on the binding position of ADO and receptor conformations. Mechanistically, the binding of MIPS521 may stabilize the active conformation by interacting with the allosteric site, which, in turn, promotes the collapse of the Na^+^ pocket (a nearby conserved class A activation motif).^245,306^

#### Targeting of GPCR transmembrane domain (inside 7TMD)

**Fig. 27:**
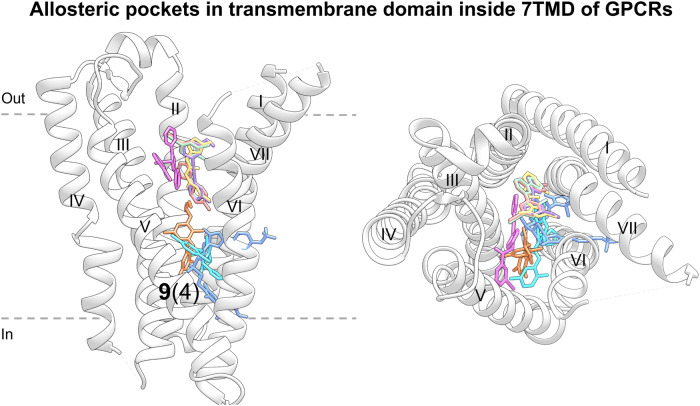
Allosteric binding sites in the transmembrane domain within 7TMD of GPCRs and corresponding small-molecule allosteric modulators. Stick models of small-molecule ligands are mapped to representative member CRF1R, PDB: 4K5Y. The number of unique modulators is indicated in boldface type, and the number of GPCRs containing the pocket is provided in parentheses

An empty pocket is present in the middle of the GPCR transmembrane domain that serves as an allosteric site inside 7TMD, as shown in Fig. 27. Allosteric modulators bound to this pocket primarily interact with helices other than TM I and TM IV. To date, a PAM of FFAR3, an allosteric antagonist of CRF1R, an allosteric agonist of PTH1R, and five NAM and one PAM of mGluR5 have been reported to bind to this region (Table 3). In contrast, the binding of NAMs and an allosteric antagonist blocks the outward movement of TM VI, thereby acting as an antagonist. Notably, the binding of only the allosteric agonist stabilizes the G protein through direct interactions.

**Table 3:** Solved GPCR structures complexed with synthetic allosteric modulators bound to the transmembrane domain inside 7TMD

Structure Type	GPCR type	GPCR	Modulator	Highest Phase	Modulator type	Number	PDB code	Allosteric site	Refs
Cryo-EM	class A	FFAR3	AR420626	Pre-clinical	PAM	(52)	8J20	inside 7TMD	^469^
X-ray diffraction	class B	CRF1R	CP-376395	Pre-clinical	Allosteric antagonist	(53)	4K5Y	inside 7TMD	^470^
Cryo-EM	class B	PTH1R	PCO371	Pre-clinical	Allosteric agonist	(54)	8GW8	inside 7TMD	^335^
X-ray diffraction	class C	mGluR5	mavoglurant	Phase 3	NAM	(55)	4OO9	inside 7TMD	^178^
X-ray diffraction	class C	mGluR5	compound 14	Pre-clinical	NAM	(56)	5CGC	inside 7TMD	^321^
X-ray diffraction	class C	mGluR5	HTL14242	Phase 1	NAM	(57)	5CGD	inside 7TMD	^321^
X-ray diffraction	class C	mGluR5	Fenobam	Phase 1	NAM	(58)	6FFH	inside 7TMD	^471^
X-ray diffraction	class C	mGluR5	M-MPEP	Pre-clinical	NAM	(59)	6FFI	inside 7TMD	^471^
Cryo-EM	class C	mGluR5	CDPPB	Pre-clinical	PAM	(60)	8TAO	inside 7TMD	^325^

**1) Inside 7TMD:**

**FFAR3–AR420626, CRF1R–CP-376395, mGluR5–mavoglurant, mGluR5–compound 14, mGluR5–HTL14242, mGluR5–Fenobam, mGluR5–M-MPEP, and PTH1R–PCO371 structures**

The metabotropic glutamate (mGlu) receptor type 5 is among the eight most widely expressed mGlu receptors in the brain.^307^ Recently, mGluR5 has been shown to be involved in a growing number of cognitive and psychiatric disorders including schizophrenia.^308–310^ The mGlu receptor family is abundant in allosteric pharmacology among the class C GPCRs.^311–315^ In particular, allosteric regulation of mGlu5 receptors has received significant attention as a novel modality to treat diseases, including Parkinson’s disease and schizophrenia.^316–318^ NAMs of the mGlu5 receptors may serve to normalize excessive glutamate activity without blocking the physiological roles of the brain; thus, they are regarded as prospective agents for the treatment of multiple neurological disorders.^319,320^

**Fig. 28:**
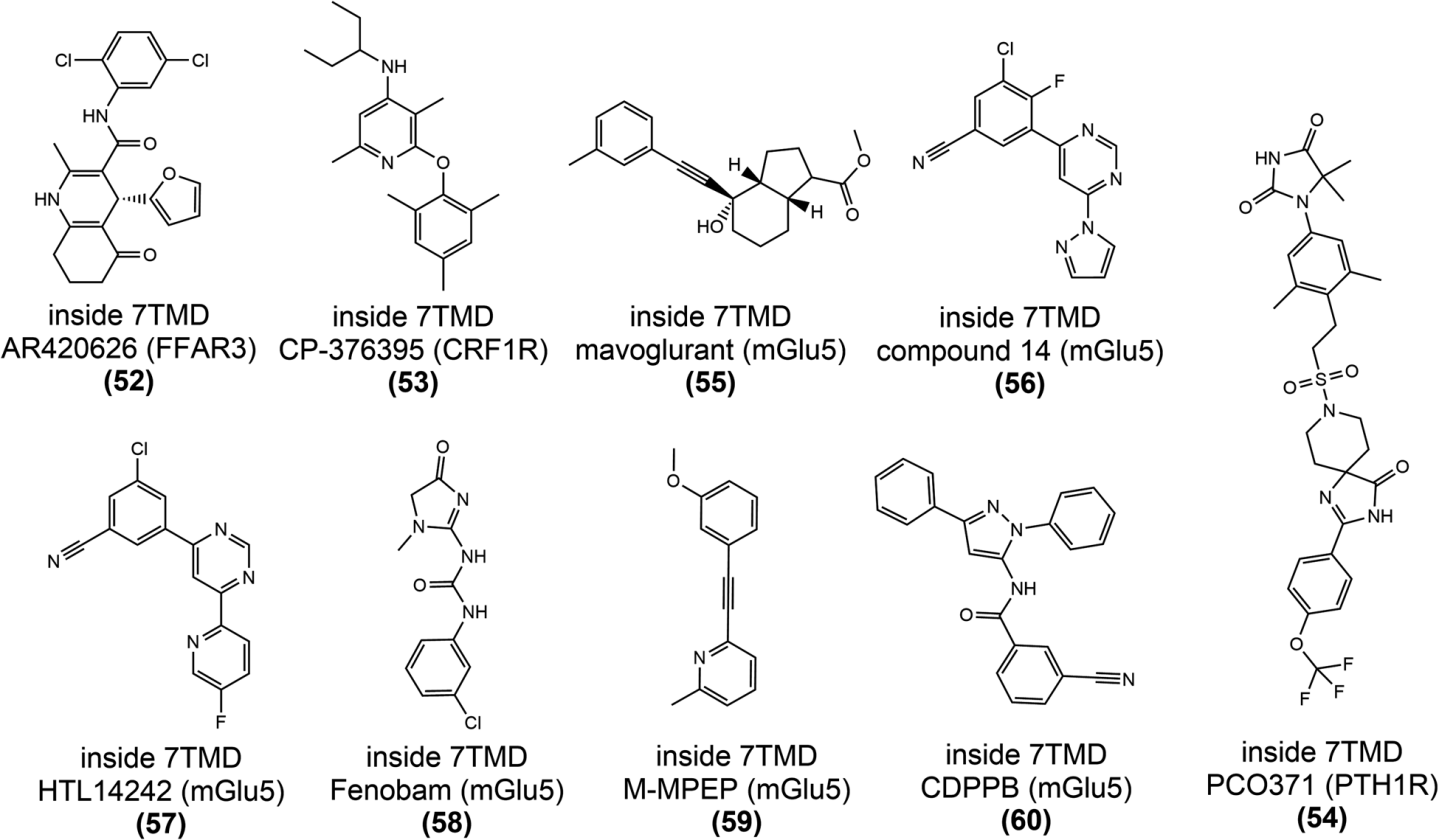
2D chemical structures of synthetic small-molecule allosteric ligands targeting the transmembrane domain within 7TMD of GPCRs

**Fig. 29:**
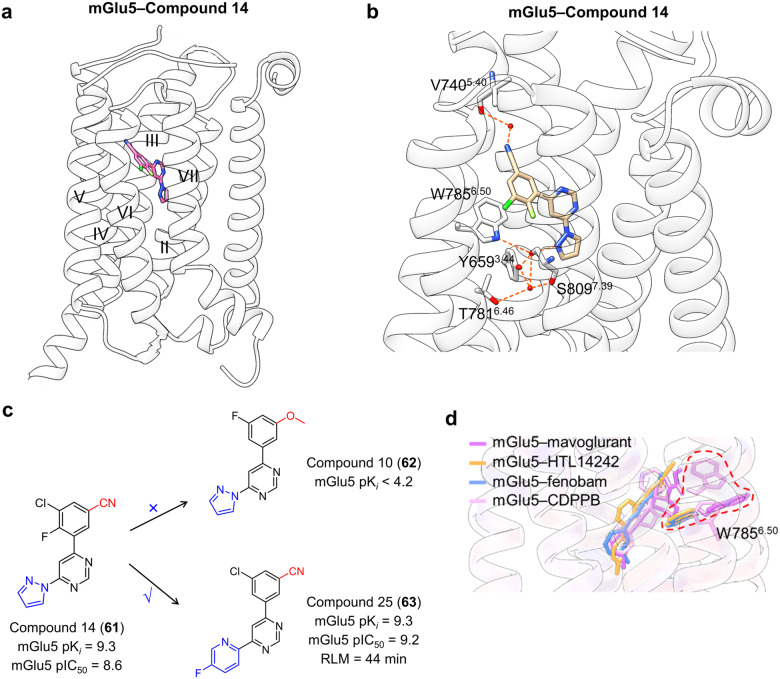
**a** Schematic representation of the NAM fenobam bound to mGlu5 receptor (PDB: 5CGC). **b** Detailed binding modes of mGlu5 receptor binding to compound 14; polar interactions are shown in orange dashes. **c** SAR optimization of mGlu5 receptor NAMs. **d** Superimposed views of the highlighted residues on mGlu5–mavoglurant (purple cartoon, purple sticks; PDB: 4OO9), mGlu5–HTL14242 (orange cartoon, orange sticks; PDB: 5CGD), mGlu5–fenobam (blue cartoon, blue sticks; PDB: 6FFH), and mGlu5–CDPPB (pink cartoon, pink sticks; PDB: 8TAO) structures

The key binding determinants of these NAMs were identified in TM III, VI, and VII (Fig. 29a). Among these, HTL14242 (**57**) (Fig. 28) is an advanced and orally active NAM that progresses in early clinical testing for the potential treatment of amyotrophic lateral sclerosis (ALS) (Fig. 29). Crystal structures of mGluR5 with the lead compound of HTL14242, compound 14 (**56**), demonstrate the underlying basis for SAR optimization. With the pyrimidine linker traversing the narrow channel in the allosteric pocket formed by Tyr659^3.44^, Ser809^7.39^, Val806^7.36^, and Pro655^3.40^, the benzene ring and pyrazole ring of compound **56** respectively sit in two sub-pockets. The presence of the nitrile moiety on the benzene ring is crucial due to its delicate orientation that mediates the formation of hydrogen bonds with Val740^5.40^ via the bridging of a water molecule (Fig. 29b). Therefore, removal or replacement of the nitrile moiety caused a substantial drop in affinity, underlying the significance of maintaining this moiety during SAR studies (proved by compounds **57** and **58**) (Fig. 29c). Notably, the conformation of the pyrazole end allows for slightly bulkier rings and small substitutes, as long as the hydrogen bond between the nitrogen atom and Ser809^7.39^, and the surrounding polar network is maintained (Fig. 29b). Such SBDD analysis thereupon yielded HTL14242, which harbors highly similar binding modes with **56** while simultaneously benefiting from its favorable physicochemical and pharmacokinetic properties (Fig. 29c).^321^

Furthermore, by aligning mGlu5-NAM structures, a common salt bridge interaction was found between the residue pair Lys665^3.50^ and Glu770^6.35^, whereas it was absent in the PAM-bound complex, suggesting that NAM hinders the outward motion of TM VI by enhancing the interactions between TM III and TM VI at the cytoplasmic end.^322–324^ Conformational transitions of the highly conserved Trp785^6.50^ occur in the allosteric modulator-bound mGluR5 structure to adjust the size of the allosteric pocket and accommodate binding (Fig. 29d).^178,325^ Trp785^6.50^ within the FxxCWxP^6.50^ motif serves as a “toggle switch” in class A receptors, undergoing a conformation change during activation.^321,326^ It is plausible that this residue may also play a role in class C GPCR activation, though further investigation is required to elucidate its involvement.^178^

Other allosteric inhibitors bound to the transmembrane domain include allosteric PTH1R agonists. The parathyroid hormone receptor (PTH1R), a class B1 GPCR,^327^ is activated by parathyroid hormone and parathyroid hormone-related peptides and plays a central role in maintaining mineral ion homeostasis and skeletal metabolism.^328–330^ Recently, a highly selective PTH1R agonist, the orally active non-peptidic small molecule PCO371 (**54**) (Fig. 28), was identified and is currently undergoing phase 1 clinical trials to treat hypoparathyroidism.^331,332^ PCO371 consists of four chemical modules, from left to right: trifluoromethoxyphenyl, spiro-imidazolone, dimethylphenyl, and dimethylhydantoin.^333^

**Fig. 30:**
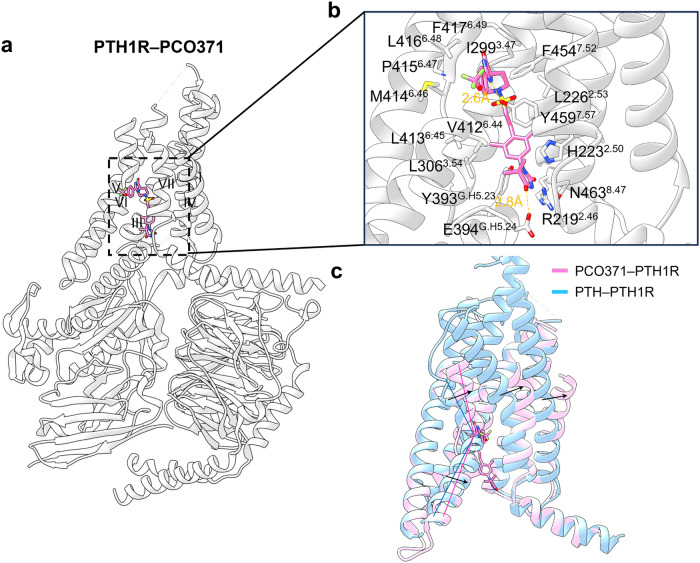
**a** Schematic representation of the allosteric agonist PCO371 bound to PTH1R (PDB: 8GW8). **b** Detailed binding modes of PTH1R binding to PCO371. Hydrogen bonds are presented as orange dashes. **c** Superposition of the PTH1R bound to PCO371 (displayed in pink) and PTH (displayed in blue) reveals conformational changes upon PCO371 binding

The solved crystal structure complex of PTH1R–PCO371 reveals important information regarding the PCO371 binding cavity,^334^ which was observed to reside in a pocket surrounded by residues on TM II, TM III, TM VI, and TM VII, analogous to mGluR5 (Fig. 30a).^335,336^ In the PCO371 binding site, the NH moiety of the spiro-imidazolone in PCO371 is engaged in a hydrogen binding interacts with Tyr459^7.57^ (Fig. 30b). The carbonyl group of dimethylhydantoin in PCO371 forms a salt bridge interaction with the protonated nitrogen of Arg219^2.46^. In addition to polar contacts, the trifluoromethoxyphenyl group formed nonpolar interactions with Met414^6.46^, Leu416^6.48^, and Phe454^7.52^; the spiroimidazolone group formed hydrophobic interactions with Leu226^2.53^, Ile299^3.47^, Pro415^6.47^, and Phe417^6.49^; the dimethylphenyl group formed hydrophobic interactions with His223^2.50^, Leu306^3.54^, Val412^6.44^, Leu413^6.45^ and Tyr459^7.57^; and the dimethylhydantoin group formed hydrophobic interactions with Asn463^8.47^. Notably, Glu394^G.H5.24^ in the Gs protein was hydrogen-bonded to the carbonyl group of dimethylhydantoin, and Tyr393^G.H5.23^ formed a hydrophobic interaction with dimethylhydantoin, thus stabilizing the ternary PCO371–PTH1R–Gs complex.

As the binding of PCO371 precludes the endogenous ligand from occupying the core of the 7TM helical bundle, only structures in which PCO371 alone binds to PTH1R exist. The presence of PCO371 induced inward displacement of the extracellular and cytoplasmic termini of TM6 cells (Fig. 30c). Comparison of the structures of PCO371–PTH1R and CP-376395–CRF1R revealed that PCO371 has a deeper binding site and therefore does not impede the formation of the conserved Pro^6.47^-X-X-Gly^6.50^ kink, as CP-376395 does. In addition, the binding site of PCO371 is shallower than that of allosteric modulators that bind to the intracellular surface inside 7TMD and therefore does not impede G-protein or arrestin binding. Till date, PCO371 is probably the only receptor allosteric agonist reported to stabilize the active state through direct interactions with downstream transducers.

#### Targeting of GPCR Intracellular Surface (outside and inside 7TMD)

**Fig. 31:**
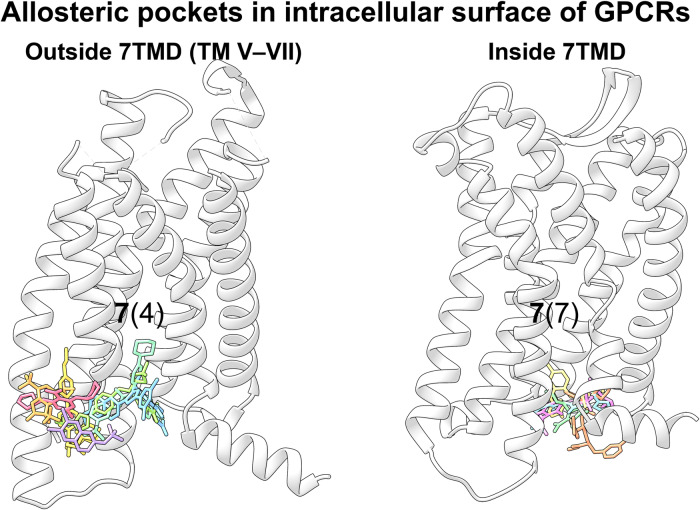
Two intracellular allosteric binding sites in GPCRs and the corresponding small-molecule allosteric modulators. Stick models of small-molecule ligands are mapped to representative members of outside 7TMD (V−VII) (GCGR, PDB: 5EE7) and inside 7TMD (CCR2, PDB: 5T1A) GPCRs. For each pocket, the number of unique modulators is indicated in boldface type, and the number of GPCRs containing the pocket is provided in parentheses

In these instances, allosteric modulators bind to interfaces located between the cytoplasmic terminus of the 7TM helical bundle and downstream transducers,^337^ including the outside and inside of 7TMD.^46^ Till date, only two different binding sites in the intracellular surface have been identified using crystal structures (Table 4), namely the pocket outside helices V − VII and the pocket inside 7TMD (Fig. 31).

**Table 4:** Solved GPCR structures complexed with synthetic allosteric modulators bound to the intracellular surface

Structure Type	GPCR type	GPCR	Modulator	Highest Phase	Modulator type	Number	PDB code	Allosteric site	Refs
Cryo-EM	class A	GPR88	2-PCCA	Pre-clinical	Allosteric agonist	(64)	7EJX	outside 7TMD (V−VII)	^472^
X-ray diffraction	class B	GCGR	MK-0893	Phase 2	Allosteric antagonist	(65)	5EE7	outside 7TMD (V−VII)	^363^
X-ray diffraction	class B	GCGR	NNC0640	Pre-clinical	NAM	(66)	5XEZ	outside 7TMD (V−VII)	^364^
X-ray diffraction	class B	GLP-1R	NNC0640	Pre-clinical	NAM	(66)	5VEX	outside 7TMD (V−VII)	^365^
X-ray diffraction	class B	GLP-1R	PF-06372222	Pre-clinical	NAM	(67)	5VEW	outside 7TMD (V−VII)	^365^
Cryo-EM	class B	GLP-1R	compound 2	Pre-clinical	ago-PAM	(68)	7EVM	outside 7TMD (V−VII)	^358^
Cryo-EM	class C	GABA_B_	GS39783	Pre-clinical	PAM	(69)	6UO8	outside 7TMD (V−VII)	^352^
Cryo-EM	class C	GABA_B_	BHFF	Pre-clinical	ago-PAM	(70)	7C7Q	outside 7TMD (V−VII)	^351^
X-ray diffraction	class A	β2AR	Cmp-15PA	Pre-clinical	Allosteric antagonist	(71)	5X7D	inside 7TMD	^473^
X-ray diffraction	class A	CCR2	CCR2-RA-[R]	Pre-clinical	Allosteric antagonist	(72)	5T1A	inside 7TMD	^474^
X-ray diffraction	class A	CCR9	vercirnon	Phase 3	Allosteric antagonist	(73)	5LWE	inside 7TMD	^385^
X-ray diffraction	class A	CCR7	Cmp2105	Pre-clinical	Allosteric antagonist	(74)	6QZH	inside 7TMD	^475^
X-ray diffraction	class A	CXCR2	00767013	Pre-clinical	Allosteric antagonist	(75)	6LFL	inside 7TMD	^476^
Cryo-EM	class A	GPR61	Compound 1	Pre-clinical	Allosteric inverse agonist	(76)	8TB7	inside 7TMD	^477^
Cryo-EM	class A	NTSR1	SBI-553	Pre-clinical	PAM	(77)	8JPB	inside 7TMD	^478^

**1) Outside 7TMD (V−VII):**

**GPR88−2-PCCA, GCGR−MK-0893, GCGR−NNC0640, GLP-1R−PF-06372222, GLP-1R−compound 2, GABA**_**B**_ **−GS39783, and GABA**_**B**_ **−BHFF structures**

The reported allosteric sites on the intracellular surface outside 7TMD are all attached to TM VI, located between helices V and VII, suggesting that TM VI plays a functional role in transmitting ligand-binding information to the orthosteric pocket.^338^ G-protein coupling requires outward motion of the intracellular end of TM VI, a key signature for activating GPCR,^339^ which inspired the design of small-molecule allosteric modulators that target this site.

The GABA_B_ receptor, classified as a heterodimeric class C GPCR, comprises two distinct subunits, GB1 and GB2, which can be activated by γ-aminobutyric acid (GABA), a primary inhibitory neurotransmitter in the central nervous system.^340,341^ GABA_B_ receptors are key therapeutic targets in the treatment of multiple neurological disorders, including depression, schizophrenia, and drug addiction.^342–344^ Dimerization of GABA_B_ receptors results in the emergence of this new dimer-specific allosteric binding site (Fig. 33a),^345–349^ which prevents GS39783 (**69**) and BHFF (**70**) from acting on the monomer, but instead specifically controls GPCR dimer activity.

**Fig. 32:**
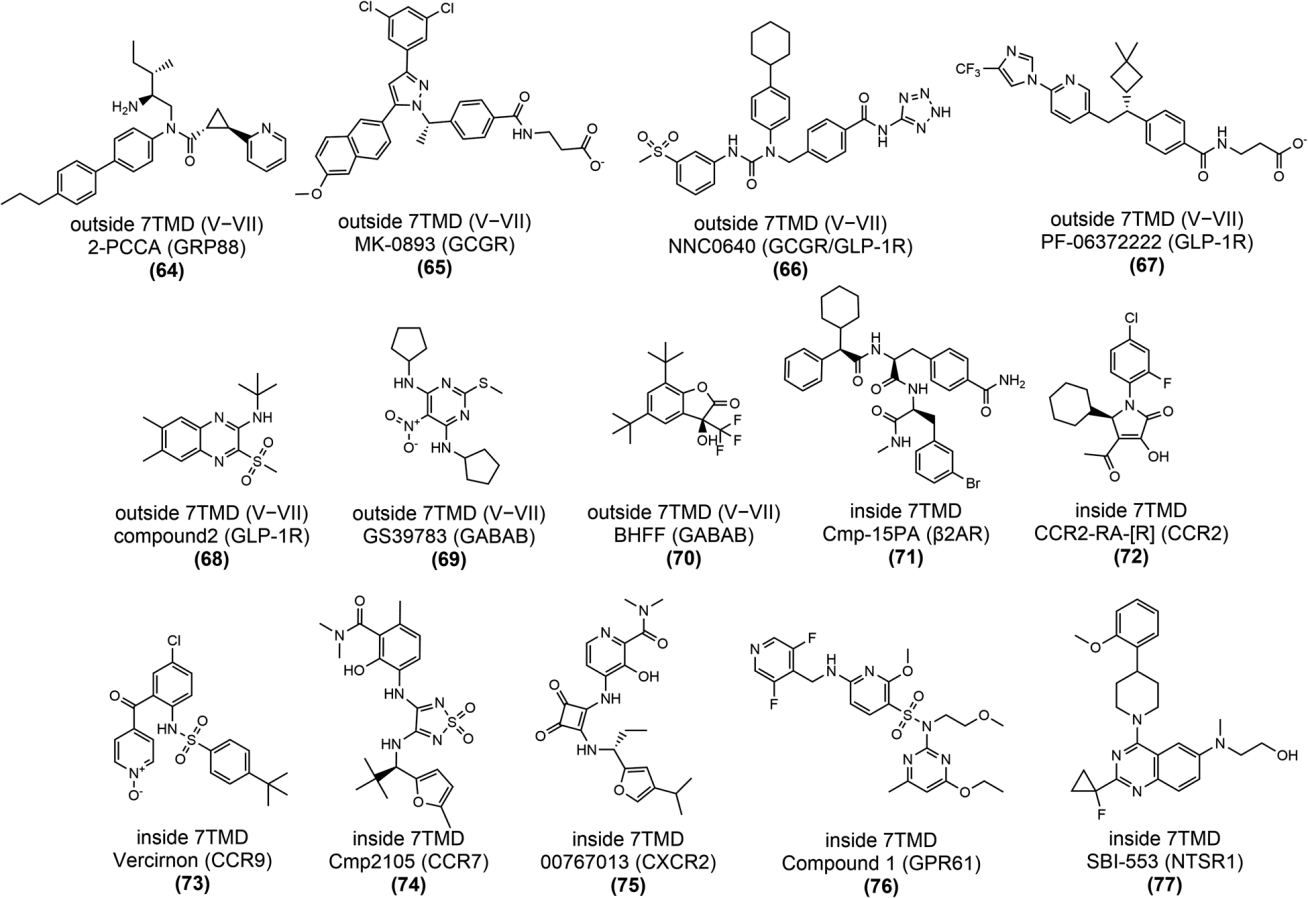
2D structures of synthetic allosteric ligands targeting the GPCR intracellular surface

**Fig. 33:**
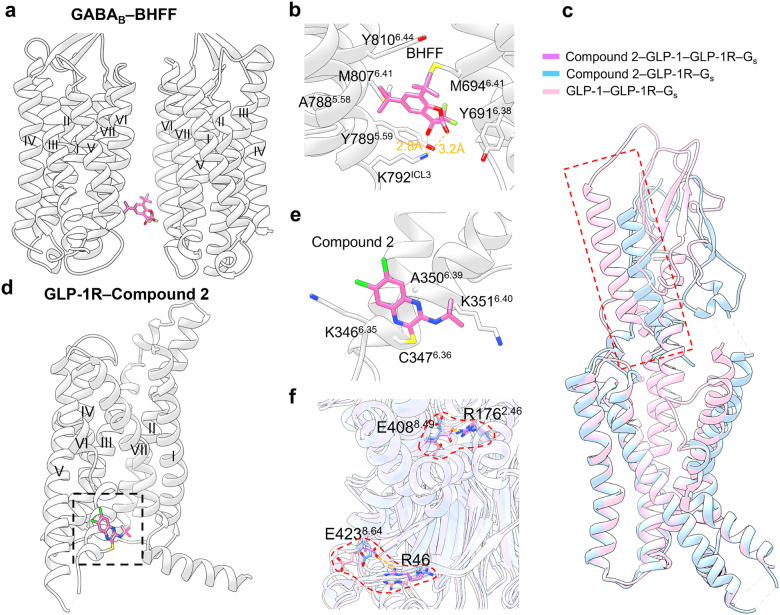
**a** Schematic representation of PAM BHFF bound to GABA_B_ receptor (PDB: 7C7Q). **b** Detailed binding modes of GABA_B_ receptor binding to BHFF. Hydrogen bonds are presented by orange dashes. **c** Superimposed views of the N-terminal α-helix on compound 2–GLP-1R–G_s_ (blue cartoon, blue sticks; PDB: 7DUR) and GLP-1–GLP-1R–G_s_ (pink cartoon, pink sticks; PDB: 6×18) structures. **d** Schematic representation of ago-PAM compound 2 bound to GLP-1R (PDB: 7EVM). **e** Detailed binding modes of GLP-1R bound to compound 2. **f** Superimposed views of highlighted residues on compound 2–GLP-1R–G_s_ (blue cartoon, blue sticks; PDB: 7DUR), GLP-1–GLP-1R–G_s_ (pink cartoon, pink sticks; PDB: 6×18), and compound 2–GLP-1–GLP-1R–G_s_ (purple cartoon, purple sticks; PDB: 7DUQ) structures

BHFF (Fig. 32), a potent PAM of the GABA_B_ receptor that enhances receptor potency only when activated by orthosteric agonists, lacks efficacy in mouse experiments.^350^ Determination of the BHFF−GABA_B_ receptor co-crystal structure clearly shows that BHFF is bound between TM V−TM VI of GB1 and TM VI of GB2 within the intracellular tips (Fig. 33a).^351^ The 3-hydroxy group and ketone of BHFF formed two hydrogen bonds with the Lys792^ICL3^ side chain of GB1 (Fig. 33b). For hydrophobic interactions, BHFF is buried in a cavity defined by the residues Ala788^5.58^, Tyr789^5.59^, Met807^6.41^, Tyr810^6.44^ of GB1, and Tyr691^6.38^ and Met694^6.41^ of GB2.

Compared with GS39783, another PAM molecule for the GABA_B_ receptor, both PAM molecules bind to highly similar locations within the dimer interface, and the overall complex structure is almost identical. In contrast to the binding of the orthosteric agonist baclofen alone, the PAM induces a straightening and inward shift of TM3 and TM5 in GB2 on the intracellular side and stabilizes the TM6-mediated dimerization. This alteration contributes to the stabilization of the receptor in its active state.^352–354^

Compound 2 (**68**) (Fig. 32) is a novel ago-PAM of GLP-1R that covalently binds to Cys347^6.36^ on helices VI and displays partial and almost full agonism.^355,356^ However, clinical trials of compound 2 failed because of its poor pharmacokinetic properties.^357^ In the co-crystal structure of GLP-1R complexed with compound 2, it was demonstrated that compound 2 was located on the TM VI membrane-facing surface and formed interactions only with residues on TM VI (Fig. 33d), among which a covalent disulfide bond was formed between the sulfonic group of compound 2 and Cys347^6.36^.^358^ In addition, the tert-butyl group of compound 2 extended toward TM VII and formed nonpolar interactions with Ala350^6.39^ and Lys351^6.40^ (Fig. 33e). The dichloroquinoxaline group was directed toward ICL3, forming van der Waals contacts with Lys346^6.35^ and Cys347^6.36^.

The binding of compound 2 alone, GLP-1 alone, and compound 2 − GLP-1 together all led to a similar extent of outward movement in the intracellular terminus of TM6 (Fig. 33c). Nonetheless, in the compound 2–GLP-1–GLP-1R–G_s_ structure, two additional salt bridge interactions have arisen (R176^2.46^ and E408^8.49^, E423^8.64^ and R46 in Gβ) (Fig. 33f), offering the potential to strengthen G-protein binding through long-range allosteric communications.^358^ Furthermore, the N-terminal α-helix of the GLP‐1 R extends downward into the orthosteric pocket upon binding to compound 2, which may serve to stabilize the active conformation (Fig. 33c).^359^

Covalent binding confers a higher potential energy barrier upon compound 2 dissociation, thus minimizing potential off-target effects.^360–362^ This suggests that the protruding free cysteine residues in the transmembrane helix provide an opportunity to develop irreversible GPCR allosteric modulators.

All allosteric antagonists and NAMs (MK-0893 (**65**), NNC0640 (**66**), and PF-06372222 (**67**)) bind to the other side of TM VI near TM VII and appear to be well conserved with respect to the allosteric site and binding modes.^363–365^ In this case, the protruding TM VI can serve as a base for the attachment of modulators in a clamp shape. The binding of such allosteric modulators requires certain conditions, such as a cleft between helices for the insertion of the pincers and adequate hydrophobic contacts with the surrounding residues for stable binding. The outward displacement within the TM VI required for transducer binding is restricted, preventing receptor activation.^366^

The glucagon receptor belongs to the class B GPCR and plays a critical role in the maintenance of glucose homeostasis.^367,368^ Given the key role of glucagon in elevating glycemia, small-molecule antagonists targeting GCGR are considered promising treatments for diabetes.^369–371^ In support of this concept, a novel allosteric antagonist targeting GCGR, MK-0893 (Fig. 32), has advanced into phase 2 clinical studies to treat type 2 diabetes mellitus;^372^ however, side effects, including increased LDL-C, have hindered its clinical use.^373^

**Fig. 34:**
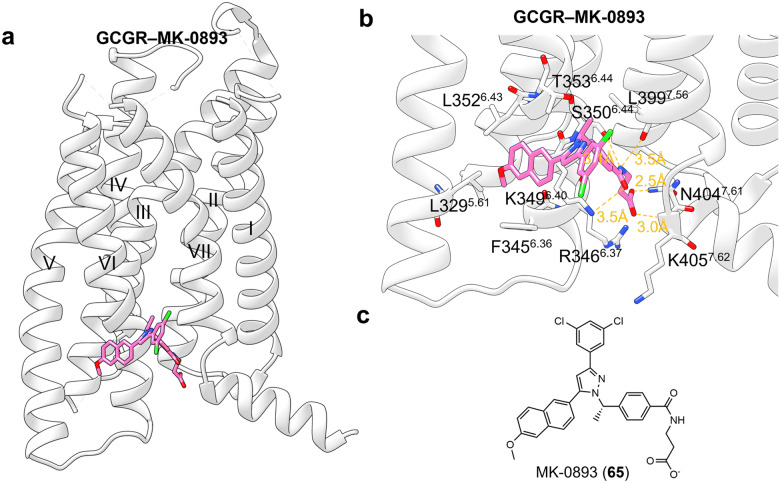
**a** Schematic representation of the allosteric antagonist MK-0893 bound to GCGR (PDB: 5EE7). **b** Detailed binding modes of GCGR binding to MK-0893. Hydrogen bonds are presented as orange dashes. **c** 2D structure of small-molecule allosteric ligand MK-0893 shown for clarity

The X-ray crystal structure of MK-0893 with GCGR was solved, revealing the atomic details of allosteric modulator binding.^363^ In this structure, MK-0893 is situated within an intracellular pocket outside helices V–VII (Fig. 34a). In terms of polar interactions, the terminal anionic carboxylic acid moiety of MK-0893 (Fig. 34c) established a salt-bridge interaction with Arg346^6.37^ (Fig. 34b).^374^ It contributes to the establishment of a hydrogen-bonding network involving the side chain of Asn404^7.61^ and the main-chain amine of Lys405^7.62^. It also forms a water-mediated hydrogen bond with the side chain of Ser350^6.44^. Additionally, the amide group of the ligand formed hydrogen bonds with the backbone carbonyl groups of Ser350^6.41^, Leu399^7.56^, and the side chain of Lys349^6.40^. Regarding hydrophobic interactions, the methoxynaphthalene moiety wedges into a cavity formed by helices V and VI and interacts with residues Leu329^5.61^, Phe345^6.36^, Leu352^6.43^, and Thr353^6.44^, and the alkyl chain of Lys349^6.40^. The phenylethylpyrazole core scaffold engages in hydrophobic interactions with residues Thr353^6.44^, and Leu399^7.56^, and forms a π−cation interaction with Lys349^6.40^. This structure closely resembles other inactive structures (PDB:5XEZ, 8JRV, and 8JRU)^375^ and does not exhibit specific conformations induced by MK-0893.

**2) Inside 7TMD:**

**β2AR–Cmp-15PA, CCR2–CCR2-RA-[R], CCR9–vercirnon, CCR7–Cmp2105, CXCR2–00767013, GPR61–Compound 1, and NTSR1–SBI-553 structures**

In contrast to orthosteric ligands, GPCRs utilize their intracellular surface inside 7TMD to directly mediate downstream signaling via G proteins or arrestins. This site has also been found to act as a binding site for allosteric regulators and is quite similar in all solved complexes (a cavity composed of the cytoplasmic region of helices I, II, VI, and VII) (Fig. 31).^24,376,377^ Allosteric antagonists bound to this region inhibit GPCR-mediated signaling through a novel dual mechanism, which appears to be a powerful way to antagonize the receptor. These allosteric modulators not only compete with G-proteins or arrestins by occupying their binding sites but also display π-π interactions (parallel or T-shaped) with Tyr^7.53^ from the conserved NPxxY motif in TM VII, acting as a molecular glue to hold together the cytoplasmic ends of the helical bundle, further preventing conformational transitions associated with activation. Therefore, targeting this druggable allosteric pocket in receptors using small-molecule allosteric antagonists may provide new opportunities for the discovery of GPCR drugs.

Chemokine receptors and their ligands are involved in chemotactic trafficking during the inflammatory responses.^378–380^ CC chemokine receptor 9 (CCR9) activated by CCL25 has emerged as a potential therapeutic target for inflammatory bowel disease because it is essential for mediating leukocyte homing to the gut.^381–383^

Vercirnon (**73**) (Fig. 32), an allosteric antagonist of CCR9 used in Crohn’s treatment, has progressed to Phase 3 clinical studies. However, its efficacy is limited due to the requirement for significantly high doses to effectively block receptor activation.^384^ This uncertainty may stem from the fact that drug molecules might not necessarily bind to the intracellular G protein region upon entering the cytoplasm.^271^ This challenge could be common among this class of modulators, but only vercirnon has entered clinical trials at present.

**Fig. 35:**
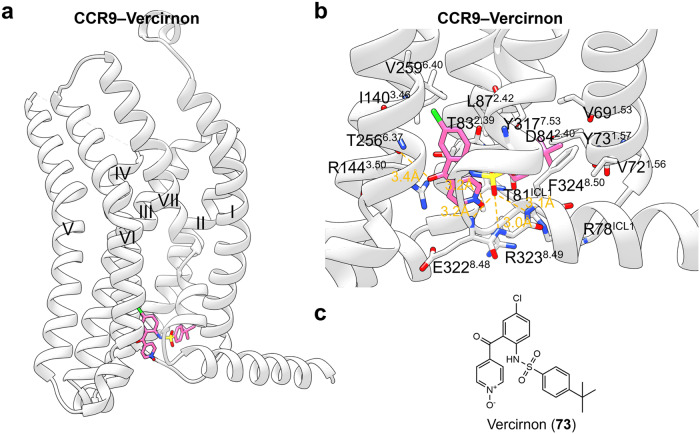
**a** Schematic representation of the allosteric antagonist vercirnon bound to CCR9 (PDB: 5LWE). **b** Detailed binding modes of CCR9 binding to vercirnon. Hydrogen bonds are presented as orange dashes. **c** 2D structure of small-molecule allosteric ligand vercirnon shown for clarity

The binding site of vercirnon has been shown via X-ray crystallography to be the center of the 7TM helical bundle on the intracellular side (Fig. 35a). The sulfone group (Fig. 35c) of vercirnon engages in a trivalent hydrogen bond with the main-chain amino groups of three neighboring residues: Glu322^8.48^, Arg323^8.49^, and Phe324^8.50^.^385^ Additionally, the pyridine-N-oxide group is hydrogen-bonded to the side chain of Thr81^ICL1^, and the ketone group forms a hydrogen bond with the side chain of Thr256^6.37^ (Fig. 35b). Regarding hydrophobic interactions, the tert-butylphenyl group is deeply buried in the hydrophobic pocket surrounded by Val69^1.53^, Val72^1.56^, Tyr73^1.57^, Leu87^2.42^, Tyr317^7.53^, and Phe324^8.50^. The chlorophenyl group was situated within a narrow crevice formed by the hydrophobic parts of residues Leu87^2.43^, Ile140^3.46^, Val259^6.40^, and Tyr317^7.53^. Furthermore, the pyridine-N-oxide group is enclosed within a polar cavity formed by residues Thr81^ICL1^, Thr83^2.39^, Asp84^2.40^, Arg144^3.50^, and Arg323^8.49^.

## Conclusions and perspectives

Over the past few decades, crystallographic, biochemical, and computational studies have provided unprecedented atomic and structural insights into the comprehensive regulation of GPCR structures.^386–389^ Till date, 388 orthosteric modulators, as well as 717 complex structures of GPCRs bound to the orthosteric modulators, have been reported. In addition, 53 different allosteric small-molecule modulators bound to GPCR complex structures and nine different allosteric sites were also solved. In this study, we first review the structure advances, signaling mechanisms, and functional diversity of GPCRs to outline the current profile and up-to-date development in this field. Importantly, by means of the aforementioned crystallographic advancements, orthosteric and allosteric modulators are discussed separately in terms of their complex structures, signaling mechanisms, and consequential implications for drug discovery. Such in-depth investigation underlines the significance of understanding GPCR structures and mechanisms for developing effective therapeutics.

Because of the relatively large amount of orthosteric modulators and their shared functional mechanism of competing with the endogenous ligands, representative cases of orthosteic drugs launched within the past five years have been carefully selected and dissected, including μ-OR-oliceridine complex (stand-out for G-protein biased signaling), S1PR-siponimod complex (stand-out for subtype selectivity and “toggle switch” activation mechanism), OX2R-lemborexant complex (stand-out for kinetics and dynamics parameters), 5-HT_1F_-lasmiditan complex (distinctive for subtype selectivity), and GnRH1-elagolix complex (distinctive for signal transmission based on atypical receptor structure). Structural analysis revealed that modulators are stabilized within the orthosteric pockets by key polar interactions with residues on the TM bundles, in which residues that are unconserved among the family subtypes are often determinants of selectivity. Furthermore, the “toggle switch”, PIF, DRY, and NPxxY motifs are critical mechanical switches that transmit extracellular stimuli to the intracellular regions, and some specific polar interactions between TM3 and TM5/6 may also serve as a boost for the displacement of TM5/6, which is a hallmark of receptor activation/deactivation.

Since the binding patterns and action mechanisms of allosteric modulators tend to be unique, we placed special emphasis on the depiction of allosteric regulators.^390^ From the analysis of these structural complexes, the extracellular vestibule inside 7TMD was identified as the most prevalent binding site for allosteric modulators, and induced-fit shape matching and charge matching are the determinants of allosteric ligand accommodation. The binding of allosteric modulators alters the free-energy landscape and stabilizes the different dominant conformations of the receptor.^391^ Specifically, we established a novel classification of allosteric modulators into two categories. First, the allosteric effects of the modulators can be realized by directly modulating the binding of orthosteric ligands or intracellular transducer proteins to receptors. In this instance, allosteric modulators may occupy a pocket above the orthosteric binding pocket and interact directly with orthosteric ligands and/or surrounding residues to alter association or dissociation. Allosteric modulators may also occupy the binding pockets of G-proteins and arrestins to impede their binding or occupy the pockets above their binding sites to regulate binding. Second, allosteric modulators regulate the ability of receptor complexes to interact with intracellular transducer proteins by indirectly altering their activation pathways. In this instance, allosteric modulators appear to act as steric wedges, stabilizing or destabilizing certain interactions to restrict or facilitate conformational rearrangements of the receptor.

In addition to the commonalities summarized above, it is of broad pharmaceutical interest to understand how the drug recognition basis and delicate mechanistic regulation of orthosteric/allosteric modulators can expand upon drug design and why we need to pursue this line of research.^117,392,393^ From a structural and chemical biology perspective, understanding the mechanical switch cascade assists in acquiring insights into fine-tuning regulation and enables human control of different downstream functions of GPCRs.^62^ Therefore, GPCRs after mutagenesis may be employed as biosensors to initiate distinct intracellular signaling events.^394^

Medicinal chemists can be inspired by five perspectives based on the analysis presented in this review:

1. With orthosteric small-molecule modulators remaining the mainstream therapeutic agents for GPCRs, the rational design of orthosteric ligands with high affinity, efficacy, and selectivity has been a long-standing topic.^395–398^ Concerning with enhancement of affinity and efficacy, designing ligands that constitute “anchors” and “drivers” has been suggested.^399,400^ The “anchors” represent the primary parts of the ligands that contribute to affinity by binding with utmost residues and remain nearly unchanged during the transition between different states of the receptor. The “drivers” should be designed to form certain interactions with “toggle switches” and thus trigger activation/deactivation signaling. The “anchors” provide a foundation that allows the “drivers” to exert a “pull” and/or “push” action that shifts the receptor population, thereby enhancing efficacy. This “mechanism-based drug design” concept, expanding upon the traditional “structure-based drug design” strategy, may shed light on effective and efficient GPCR drug discovery.^401,402^ Regarding the improvement of selectivity, although the orthosteric sites are highly conserved in one family, capturing slight differences in loop/TM bundle/key residue conformation from other receptor subtypes and specifically interacting with these hotspots may result in a multifold increase in selectivity, thus alleviating side effects.
2. Uncovering the appropriate allosteric pockets and further conducting allosteric drug design are also durable and questionable research topics.^403–405^ With the development of crystallography offering credible initial structures, MD simulations, together with enhanced sampling methodologies, have enabled the characterization of various intermediate conformations where cryptic allosteric sites may emerge.^406–414^ Based on the identified allosteric sites, allosteric modulators can be designed using a three-dimensional (3D) molecular generation algorithm that uses the topological surface and geometric structure.^415^ Although the low conservation of allosteric sites may result in a lack of a general formula concerning the scaffold of allosteric modulators,^416^ it can be concluded that while the hydrophobic nature of allosteric ligands will contribute to receptor binding and regulation in most instances, polar groups can also interact, especially when the modulators bind outside 7TMD and require immobilization to the receptor. Moreover, an “allosteric-like” rule condensed by our group (molecular weight ≤600; 2≤ number of rotatable bonds ≤6; number of rings ≤5; number of rings in the largest ring system= 1 or 2, 3≤ SlogP ≤7) can serve as a filter criteria when designing molecules.^417^
3. With the GPCR-ligand complex structural information in hand, the topic of whether the kinetic and dynamic properties of ligands can also be modified by medicinal chemists is considered.^177,418,419^ Based on the desirable k_on_ and k_off_ values of lemborexant analyzed above, it is inferred that designing a ligand that can assemble to its receptor-bound state in advance may help achieve a high k_on_ value, in which case a simple MD simulation of designed ligands in a solvent can acquire the preferential conformation of the ligand and guide our selection. In contrast, estimating the binding free energy of the designed ligands may help predict and rank their k_off_ values, thus providing valuable guidance for ligand optimization from a kinetic perspective.^420,421^
4. For both orthosteric and allosteric modulators, achieving biased signaling is of scientific and clinical significance for any GPCR target.^26,422–428^ With the design of G-protein-biased modulators of μ-OR as a paradigm, it is suggested that comparison of the biased and unbiased ligands binding modes may help locate the key residues or regions that contribute to biased signaling. A “mechanism-based drug design” that designs ligand forming/avoiding interactions with hotspots may lead to the successful distinguishing of promiscuous signals.
5. In the regulation of PAM and NAM, orthosteric and allosteric sites are coupled through mutual signal perturbations within the protein.^429–431^ Thus, bitopic ligands, which connect the pharmacophores of both orthosteric and allosteric ligands through a linker, may facilitate a deeper investigation of site-site coupling within GPCRs and harvest ligands with higher selectivity and biased signaling abilities.^432–437^ With GPCR bitopic ligands occupying a relatively blank zone, linker optimization while immobilizing fragments at both ends, as well as fixing the orthosteric fragment while attaching a warhead to the allosteric fragment to anchor it in an allosteric pocket containing nucleophilic amino acids, may prove to be promising strategies for exploiting this field.

Despite significant technological advances and studies on GPCR structure and drug discovery,^438–442^ obstacles still exist. As GPCR crystallography is still a time-consuming and labor-intensive process,^31^ structure-dependent scenarios can somewhat hinder the study of GPCR mechanism and drug discovery, even with the use of AlphaFold and RoseTTAFold.^443–445^ For example, modeling from AlphaFold is not precise in residue orientation and can thus mislead the analysis of signaling mechanisms.^446^ Moreover, MD simulations do not guarantee the identification of novel cryptic allosteric sites in allosteric drug design.^447^ Therefore, other novel perspectives such as sequence, coarse-grained topology,^448^ and evolution are urgently required and may alleviate structural dependencies.^449^ One possibility is the use of available large data of sequences and end-to-end concepts in deep learning to develop “sequence-to-mechanism” or “sequence-to-drug discovery” methodologies,^450^ which can help avoid error accumulation from various models if the experimental strategies fail to provide the crystal structures.

In summary, we are at a stage of in-depth research on the orthosteric and allosteric modulation of GPCRs. Although our understanding of drug-target interactions, binding hotspots, and mechanisms for small-molecule modulators has become increasingly clear, some aspects still require further study. The present study contributes to a better understanding of the ligand recognition and regulatory mechanisms of GPCRs. Furthermore, by proposing a novel classification of the mechanism of allosteric modulators and an innovative concept of “mechanism-based drug discovery,” we aim to outline the latest landscape of GPCRs and interest researchers to facilitate this field. More effective, selective, and safer small-molecule therapeutics for GPCRs should be the focus of future studies in this field.

## Supplementary information

Supplementary Material
