# Supplementary material for: G protein-coupled receptors (GPCRs): advances in structures, mechanisms and drug discovery
Source: Signal Transduct Target Ther. 2024 Apr 10;9:88. doi: 10.1038/s41392-024-01803-6 (PMC11004190; doi:10.1038/s41392-024-01803-6)
Supplement: Supplementary file 1 — Supplementary Material [file 41392_2024_1803_MOESM1_ESM.docx]

**Supplementary Materials for**

G protein-coupled receptors (GPCRs): advances in structures, mechanisms, and drug discovery

Mingyang Zhang^a,b,#^, Ting Chen^c,#^, Xun Lu^b,#^, Xiaobing Lan^a^, Ziqiang Chen^*,d^, Shaoyong Lu^*,a,b^

Correspondence to: Shaoyong Lu ([lushaoyong@sjtu.edu.cn](mailto:lushaoyong@sjtu.edu.cn)) & Ziqiang Chen (ziqiang_chensuper81@vip.163.com)

**This PDF file includes:**

Supplementary Table 1-5

Supplementary Table 1. 657 Solved class A GPCR structures complexed with synthetic orthosteric modulators

| Structure Type | GPCR Type | GPCR | Modulator | Modulator Type | PDB code |
| --- | --- | --- | --- | --- | --- |
| Cryo-EM | Class A | [DRD2](http://www.uniprot.org/uniprot/P14416) | [rotigotine](../../../ligand/3308/info) | Agonist | <8IRS> |
| Cryo-EM | Class A | [DRD3](http://www.uniprot.org/uniprot/P35462) | [rotigotine](../../../ligand/3308/info) | Agonist | <8IRT> |
| Cryo-EM | Class A | [DRD5](http://www.uniprot.org/uniprot/P21918) | [rotigotine](../../../ligand/3308/info) | Agonist | <8IRV> |
| Cryo-EM | Class A | [P2RY1](http://www.uniprot.org/uniprot/P47900) | [2MeSADP](../../../ligand/75/info) | Agonist | <7XXH> |
| Cryo-EM | Class A | [P2Y12](http://www.uniprot.org/uniprot/Q9H244) | [2MeSADP](../../../ligand/75/info) | Agonist | <7XXI> |
| X-ray diffraction | Class A | [AA2AR](http://www.uniprot.org/uniprot/P29274) | [AB928](../../../ligand/215/info) | Antagonist | <8CIC> |
| Cryo-EM | Class A | [MRGX1](http://www.uniprot.org/uniprot/Q96LB2) | [compound 16 [PMID: 31498617]](../../../ligand/982/info) | Agonist | <8HJ5> |
| Cryo-EM | Class A | [TAAR9](http://www.uniprot.org/uniprot/Q5QD04) | [2-Phenylethanaminium](../../../ligand/217395/info) | Agonist | <8IWM> |
| Cryo-EM | Class A | [TAAR9](http://www.uniprot.org/uniprot/Q5QD04) | [SPERMIDINE](../../../ligand/71085/info) | Agonist | <8IW4> |
| Cryo-EM | Class A | [TAAR9](http://www.uniprot.org/uniprot/Q5QD04) | [rotigotine](../../../ligand/3308/info) | Agonist | <8IW9> |
| Cryo-EM | Class A | [TAAR9](http://www.uniprot.org/uniprot/Q5QD04) | [Cadaverine](../../../ligand/217396/info) | Agonist | <8ITF> |
| Cryo-EM | Class A | [TAAR9](http://www.uniprot.org/uniprot/Q5QD04) | [N,N-Dimethylcyclohexylamine](../../../ligand/217408/info) | Agonist | <8IW1> |
| Cryo-EM | Class A | [TAAR9](http://www.uniprot.org/uniprot/Q5QD04) | [2-Phenylethanaminium](../../../ligand/217395/info) | Agonist | <8IW7> |
| Cryo-EM | Class A | [TAAR9](http://www.uniprot.org/uniprot/Q5QD04) | [2-Phenylethanaminium](../../../ligand/217395/info) | Agonist | <8IWE> |
| Cryo-EM | Class A | [CNR1](http://www.uniprot.org/uniprot/P21554) | [SPERMIDINE](../../../ligand/71085/info) | Agonist | <8GHV> |
| Cryo-EM | Class A | [FFAR1](http://www.uniprot.org/uniprot/O14842) | [CHEMBL4208628](../../../ligand/163459/info) | Agonist | <8EIT> |
| Cryo-EM | Class A | [FFAR1](http://www.uniprot.org/uniprot/O14842) | [docosahexaenoic acid](../../../ligand/1422/info) | Agonist | <8EJC> |
| Cryo-EM | Class A | [FFAR1](http://www.uniprot.org/uniprot/O14842) | [fasiglifam](../../../ligand/1587/info) | Agonist | <8EJK> |
| Cryo-EM | Class A | [ACM4](http://www.uniprot.org/uniprot/P08173) | [fasiglifam](../../../ligand/1587/info) | Agonist | <7TRS> |
| Cryo-EM | Class A | [ACM4](http://www.uniprot.org/uniprot/P08173) | [acetylcholine](../../../ligand/253/info) | Agonist | <7TRK> |
| Cryo-EM | Class A | [ACM4](http://www.uniprot.org/uniprot/P08173) | [iperoxo](../../../ligand/2020/info) | Agonist | <7TRQ> |
| Cryo-EM | Class A | [ACM4](http://www.uniprot.org/uniprot/P08173) | [iperoxo](../../../ligand/2020/info) | PAM | <7TRP> |
| Cryo-EM | Class A | [CNR2](http://www.uniprot.org/uniprot/P34972) | [VU0467154](../../../ligand/3972/info) | Agonist | <8GUQ> |
| Cryo-EM | Class A | [CNR2](http://www.uniprot.org/uniprot/P34972) | [iperoxo](../../../ligand/2020/info) | PAM | <8GUR> |
| Cryo-EM | Class A | [CNR2](http://www.uniprot.org/uniprot/P34972) | [LY2033298](../../../ligand/2355/info) | Agonist | <8GUS> |
| Cryo-EM | Class A | [CNR2](http://www.uniprot.org/uniprot/P34972) | [olorinab](../../../ligand/2868/info) | Agonist | <8GUT> |
| Cryo-EM | Class A | [AA2BR](http://www.uniprot.org/uniprot/P29275) | [CP55940](../../../ligand/1157/info) | Agonist | <7XY7> |
| Cryo-EM | Class A | [AA2BR](http://www.uniprot.org/uniprot/P29275) | [CHEMBL2179724](../../../ligand/82304/info) | Agonist | <7XY6> |
| Cryo-EM | Class A | [OPRK](http://www.uniprot.org/uniprot/P41145) | 1-[[4-[5-Fluoro-6-[(oxan-4-ylamino)methyl]pyridin-2-yl]phenyl]methyl]-3-(2-methylpropyl)imidazolidine-2,4-dione | Agonist | <8DZR> |
| Cryo-EM | Class A | [OPRK](http://www.uniprot.org/uniprot/P41145) | [NECA](../../../ligand/2707/info) | Agonist | <8DZQ> |
| Cryo-EM | Class A | [OPRK](http://www.uniprot.org/uniprot/P41145) | [BAY 60-6583](../../../ligand/578/info) | Agonist | <8DZP> |
| Cryo-EM | Class A | [OPRK](http://www.uniprot.org/uniprot/P41145) | [CHEMBL277863](../../../ligand/98262/info) | Agonist | <8DZS> |
| X-ray diffraction | Class A | [ADRB2](http://www.uniprot.org/uniprot/P07550) | [SALVINORIN B METHOXYMETHYL ETHER](../../../ligand/95219/info) | Agonist | <7XKA> |
| X-ray diffraction | Class A | [ADRB2](http://www.uniprot.org/uniprot/P07550) | [SALVINORIN B METHOXYMETHYL ETHER](../../../ligand/95219/info) | Agonist | <7XK9> |
| Cryo-EM | Class A | [SSR2](http://www.uniprot.org/uniprot/P30874) | [CHEMBL277863](../../../ligand/98262/info) | Agonist | <7YAC> |
| Cryo-EM | Class A | [MTLR](http://www.uniprot.org/uniprot/O43193) | [(5R,6R)-6-(methylamino)-5,6,7,8-tetrahydronaphthalene-1,2,5-triol](../../../ligand/217397/info) | Agonist | <8IBU> |
| X-ray diffraction | Class A | [OPRK](http://www.uniprot.org/uniprot/P41145) | [CHEMBL6913](../../../ligand/202216/info) | Agonist | <7YIT> |
| Cryo-EM | Class A | [5HT6R](http://www.uniprot.org/uniprot/P50406) | [Paltusotine](../../../ligand/217405/info) | Agonist | <7YS6> |
| X-ray diffraction | Class A | [OPSD](http://www.uniprot.org/uniprot/P02699) | [erythromycin](../../../ligand/1548/info) | Agonist | <7ZBE> |
| X-ray diffraction | Class A | [OPSD](http://www.uniprot.org/uniprot/P02699) | [nalfurafine](../../../ligand/2669/info) | Agonist | <8A6E> |
| X-ray diffraction | Class A | [OPSD](http://www.uniprot.org/uniprot/P02699) | [5-hydroxytryptamine](../../../ligand/139/info) | Inverse agonist | <7ZBC> |
| X-ray diffraction | Class A | [OPSD](http://www.uniprot.org/uniprot/P02699) | [Retinal (11-cis)](../../../ligand/217172/info) | Agonist | <8A6C> |
| X-ray diffraction | Class A | [OPSD](http://www.uniprot.org/uniprot/P02699) | [Retinal (11-cis)](../../../ligand/217172/info) | Inverse agonist | <8A6D> |
| X-ray diffraction | Class A | [AA2AR](http://www.uniprot.org/uniprot/P29274) | [Retinal (11-cis)](../../../ligand/217172/info) | Agonist | <8GNE> |
| X-ray diffraction | Class A | [AA2AR](http://www.uniprot.org/uniprot/P29274) | [Retinal (11-cis)](../../../ligand/217172/info) | Agonist | <8GNG> |
| Cryo-EM | Class A | [O51E2](http://www.uniprot.org/uniprot/Q9H255) | [Retinal (11-cis)](../../../ligand/217172/info) | Inverse agonist | <8F76> |
| Cryo-EM | Class A | [FFAR4](http://www.uniprot.org/uniprot/Q5NUL3) | [Sipagladenant](../../../ligand/217388/info) | Antagonist | <8ID3> |
| Cryo-EM | Class A | [FFAR4](http://www.uniprot.org/uniprot/Q5NUL3) | [istradefylline](../../../ligand/2059/info) | Agonist | <8ID4> |
| Cryo-EM | Class A | [FFAR4](http://www.uniprot.org/uniprot/Q5NUL3) | [propanoic acid](../../../ligand/3124/info) | Agonist | <8ID6> |
| Cryo-EM | Class A | [FFAR4](http://www.uniprot.org/uniprot/Q5NUL3) | [9-Hydroxyoctadecanoic acid](../../../ligand/217392/info) | Agonist | <8ID8> |
| Cryo-EM | Class A | [FFAR4](http://www.uniprot.org/uniprot/Q5NUL3) | [linoleic acid](../../../ligand/2280/info) | Agonist | <8ID9> |
| MicroED | Class A | [AA2AR](http://www.uniprot.org/uniprot/P29274) | [oleic acid](../../../ligand/2861/info) | Agonist | <8FYN> |
| Cryo-EM | Class A | [FFAR4](http://www.uniprot.org/uniprot/Q5NUL3) | [TUG-891](../../../ligand/3812/info) | Agonist | <8G59> |
| Cryo-EM | Class A | [RL3R2](http://www.uniprot.org/uniprot/Q8TDU9) | [EPA](../../../ligand/1534/info) | Antagonist | <7YK6> |
| Cryo-EM | Class A | [RL3R2](http://www.uniprot.org/uniprot/Q8TDU9) | [ZM-241385](../../../ligand/4069/info) | Agonist | <7YK7> |
| X-ray diffraction | Class A | [GRPR](http://www.uniprot.org/uniprot/P30550) | [TUG-891](../../../ligand/3812/info) | Agonist | <7W41> |
| Cryo-EM | Class A | [HCAR2](http://www.uniprot.org/uniprot/Q8TDS4) | [CHEMBL4522365](../../../ligand/172603/info) | Agonist | <7XK2> |
| Cryo-EM | Class A | [GP174](http://www.uniprot.org/uniprot/Q9BXC1) | [DC591053](../../../ligand/1307/info) | Antagonist | <7XV3> |
| Cryo-EM | Class A | [RXFP1](http://www.uniprot.org/uniprot/Q9HBX9) | [PD 176252](../../../ligand/2975/info) | Agonist | <7TMW> |
| Cryo-EM | Class A | [GPR35](http://www.uniprot.org/uniprot/Q9HC97) | [MK 6892](../../../ligand/2513/info) | Agonist | <8H8J> |
| Cryo-EM | Class A | [ACM2](http://www.uniprot.org/uniprot/P08172) | [(2~{S})-2-$l^{4}-azanyl-3-[[(2~{R})-3-octadecanoyloxy-2-oxidanyl-propoxy]-oxidanyl-oxidanylidene-$l^{6}-phosphanyl]oxy-propanoic acid](../../../ligand/217399/info) | Agonist | <7T8X> |
| Cryo-EM | Class A | [ACM2](http://www.uniprot.org/uniprot/P08172) | [H2 relaxin](../../../ligand/216470/info) | Agonist | <7T90> |
| Cryo-EM | Class A | [ACM2](http://www.uniprot.org/uniprot/P08172) | [lodoxamide](../../../ligand/2297/info) | Agonist | <7T94> |
| Cryo-EM | Class A | [ACM2](http://www.uniprot.org/uniprot/P08172) | [acetylcholine](../../../ligand/253/info) | Agonist | <7T96> |
| Cryo-EM | Class A | [AA2BR](http://www.uniprot.org/uniprot/P29275) | [acetylcholine](../../../ligand/253/info) | Agonist | <8HDO> |
| Cryo-EM | Class A | [AA2BR](http://www.uniprot.org/uniprot/P29275) | [acetylcholine](../../../ligand/253/info) | PAM | <8HDP> |
| Cryo-EM | Class A | [GP119](http://www.uniprot.org/uniprot/Q8TDV5) | [LY2119620](../../../ligand/2356/info) | Agonist | <7WCN> |
| Cryo-EM | Class A | [GP119](http://www.uniprot.org/uniprot/Q8TDV5) | [acetylcholine](../../../ligand/253/info) | PAM | <7WCM> |
| Cryo-EM | Class A | [OPRM](http://www.uniprot.org/uniprot/P42866) | [LY2119620](../../../ligand/2356/info) | Agonist | <7T2G> |
| Cryo-EM | Class A | [OPRM](http://www.uniprot.org/uniprot/P42866) | [BAY 60-6583](../../../ligand/578/info) | Agonist | <7T2H> |
| Cryo-EM | Class A | [OPRM](http://www.uniprot.org/uniprot/P42866) | [adenosine](../../../ligand/280/info) | Agonist | <7U2K> |
| Cryo-EM | Class A | [ACM3](http://www.uniprot.org/uniprot/P20309) | [AR231453](../../../ligand/448/info) | Agonist | <8E9Y> |
| Cryo-EM | Class A | [ACM3](http://www.uniprot.org/uniprot/P20309) | [MBX-2982](../../../ligand/2422/info) | Agonist | <8EA0> |
| Cryo-EM | Class A | [ACM3](http://www.uniprot.org/uniprot/P20309) | [CHEMBL58362](../../../ligand/197137/info) | Agonist | <8E9W> |
| Cryo-EM | Class A | [ACM3](http://www.uniprot.org/uniprot/P20309) | [LOFENTANIL](../../../ligand/98820/info) | Agonist | <8E9Z> |
| Cryo-EM | Class A | [ACM4](http://www.uniprot.org/uniprot/P08173) | [CHEMBL147044](../../../ligand/39003/info) | Agonist | <8E9X> |
| X-ray diffraction | Class A | [OX2R](http://www.uniprot.org/uniprot/O43614) | [Clozapine N-oxide](../../../ligand/217380/info) | Agonist | <7XRR> |
| Cryo-EM | Class A | [OPRM](http://www.uniprot.org/uniprot/P35372) | [iperoxo](../../../ligand/2020/info) | Agonist | <8EF6> |
| Cryo-EM | Class A | [OPRM](http://www.uniprot.org/uniprot/P35372) | [CHEMBL73538](../../../ligand/202922/info) | Agonist | <8EFL> |
| Cryo-EM | Class A | [OPRM](http://www.uniprot.org/uniprot/P35372) | [iperoxo](../../../ligand/2020/info) | Agonist | <8EF5> |
| Cryo-EM | Class A | [OPRM](http://www.uniprot.org/uniprot/P35372) | [CHEMBL73538](../../../ligand/202922/info) | Antagonist | <8EFB> |
| Cryo-EM | Class A | [OPRM](http://www.uniprot.org/uniprot/P35372) | [lemborexant](../../../ligand/2250/info) | Agonist | <8EFO> |
| Cryo-EM | Class A | [MRGX1](http://www.uniprot.org/uniprot/Q96LB2) | [morphine](../../../ligand/2546/info) | Agonist | <8DWH> |
| X-ray diffraction | Class A | [P2Y12](http://www.uniprot.org/uniprot/Q9H244) | [CHEMBL4452384](../../../ligand/170167/info) | Agonist | <7PP1> |
| X-ray diffraction | Class A | [HRH3](http://www.uniprot.org/uniprot/Q9Y5N1) | [fentanyl](../../../ligand/1593/info) | Agonist | <7F61> |
| Cryo-EM | Class A | [LPAR1](http://www.uniprot.org/uniprot/Q92633) | [OLICERIDINE](../../../ligand/92699/info) | Agonist | <7YU3> |
| Cryo-EM | Class A | [LPAR1](http://www.uniprot.org/uniprot/Q92633) | [PZM21](../../../ligand/3183/info) | Agonist | <7YU6> |
| Cryo-EM | Class A | [LPAR1](http://www.uniprot.org/uniprot/Q92633) | [compound 16 [PMID: 31498617]](../../../ligand/982/info) | Antagonist | <7YU8> |
| Cryo-EM | Class A | [LPAR1](http://www.uniprot.org/uniprot/Q92633) | [selatogrel](../../../ligand/217372/info) | Antagonist | <7YU4> |
| Cryo-EM | Class A | [LPAR1](http://www.uniprot.org/uniprot/Q92633) | [N-ethyl-3-fluoro-3-[3-fluoro-4-(pyrrolidin-1-ylmethyl)phenyl]cyclobutane-1-carboxamide](../../../ligand/217371/info) | Agonist | <7YU5> |
| Cryo-EM | Class A | [LPAR1](http://www.uniprot.org/uniprot/Q92633) | [[(2~{R})-2-[5-(2-hexylphenyl)pentanoylamino]-3-oxidanyl-propyl] dihydrogen phosphate](../../../ligand/217377/info) | Agonist | <7YU7> |
| Cryo-EM | Class A | [ADA2A](http://www.uniprot.org/uniprot/P08913) | [[(2~{R})-2-[5-(2-hexylphenyl)pentanoylamino]-3-oxidanyl-propyl] dihydrogen phosphate](../../../ligand/217377/info) | Agonist | <7W7E> |
| Cryo-EM | Class A | [ADA2A](http://www.uniprot.org/uniprot/P08913) | [[(2~{R})-2-[5-(2-hexylphenyl)pentanoylamino]-3-oxidanyl-propyl] dihydrogen phosphate](../../../ligand/217377/info) | Agonist | <7W6P> |
| Cryo-EM | Class A | [S1PR1](http://www.uniprot.org/uniprot/P21453) | [[(2~{R})-2-[5-(2-hexylphenyl)pentanoylamino]-3-oxidanyl-propyl] dihydrogen phosphate](../../../ligand/217377/info) | Agonist | <7VIE> |
| Cryo-EM | Class A | [S1PR1](http://www.uniprot.org/uniprot/P21453) | [[(2~{R})-2-[5-(2-hexylphenyl)pentanoylamino]-3-oxidanyl-propyl] dihydrogen phosphate](../../../ligand/217377/info) | Agonist | <7VIF> |
| Cryo-EM | Class A | [S1PR1](http://www.uniprot.org/uniprot/P21453) | [[(2~{R})-2-[5-(2-hexylphenyl)pentanoylamino]-3-oxidanyl-propyl] dihydrogen phosphate](../../../ligand/217377/info) | Agonist | <7VIH> |
| Cryo-EM | Class A | [S1PR1](http://www.uniprot.org/uniprot/P21453) | [5-(3-Bicyclo[4.2.0]octa-1,3,5-trienyl)-1,2,3,6-tetrahydropyridine](../../../ligand/217375/info) | Agonist | <7VIG> |
| Cryo-EM | Class A | [5HT2B](http://www.uniprot.org/uniprot/P41595) | [N-pyridin-4-ylisoquinolin-4-amine](../../../ligand/217374/info) | Agonist | <7SRQ> |
| Cryo-EM | Class A | [5HT2B](http://www.uniprot.org/uniprot/P41595) | [sphingosine 1-phosphate](../../../ligand/3579/info) | Agonist | <7SRR> |
| Cryo-EM | Class A | [5HT2B](http://www.uniprot.org/uniprot/P41595) | [(S)-FTY720-phosphate](../../../ligand/3526/info) | Agonist | <7SRS> |
| Cryo-EM | Class A | [5HT5A](http://www.uniprot.org/uniprot/P47898) | [icanbelimod](../../../ligand/1933/info) | Agonist | <7X5H> |
| X-ray diffraction | Class A | [AA2AR](http://www.uniprot.org/uniprot/P29274) | [icanbelimod](../../../ligand/1933/info) | Agonist | <8CU6> |
| X-ray diffraction | Class A | [AA2AR](http://www.uniprot.org/uniprot/P29274) | [Lysergide](../../../ligand/2386/info) | Agonist | <8CU7> |
| Cryo-EM | Class A | [TRFR](http://www.uniprot.org/uniprot/P34981) | [Lysergide](../../../ligand/2386/info) | Agonist | <7X1T> |
| Cryo-EM | Class A | [5HT2C](http://www.uniprot.org/uniprot/P28335) | [Lysergide](../../../ligand/2386/info) | Agonist | <8DPG> |
| Cryo-EM | Class A | [5HT2C](http://www.uniprot.org/uniprot/P28335) | [5-CT](../../../ligand/134/info) | Antagonist | <8DPI> |
| Cryo-EM | Class A | [5HT2C](http://www.uniprot.org/uniprot/P28335) | [LJ-4517](../../../ligand/2292/info) | Antagonist | <8DPF> |
| Cryo-EM | Class A | [5HT2C](http://www.uniprot.org/uniprot/P28335) | [LJ-4517](../../../ligand/2292/info) | Agonist | <8DPH> |
| Cryo-EM | Class A | [GP119](http://www.uniprot.org/uniprot/Q8TDV5) | [taltirelin](../../../ligand/3677/info) | Agonist | <7XZ6> |
| Cryo-EM | Class A | [GP119](http://www.uniprot.org/uniprot/Q8TDV5) | [psilocin](../../../ligand/3157/info) | Agonist | <7XZ5> |
| Cryo-EM | Class A | [AA2AR](http://www.uniprot.org/uniprot/P29274) | [lorcaserin](../../../ligand/2302/info) | Agonist | <7T32> |
| X-ray diffraction | Class A | [AA2AR](http://www.uniprot.org/uniprot/P29274) | [lorcaserin](../../../ligand/2302/info) | Agonist | <8DU3> |
| X-ray diffraction | Class A | [S1PR5](http://www.uniprot.org/uniprot/Q9H228) | [lorcaserin](../../../ligand/2302/info) | Agonist | <7YXA> |
| X-ray diffraction | Class A | [SSR2](http://www.uniprot.org/uniprot/P30874) | [APD668](../../../ligand/432/info) | Agonist | <7XN9> |
| Cryo-EM | Class A | [SSR4](http://www.uniprot.org/uniprot/P31391) | [(4R,7R,18E)-4,7-dihydroxy-N,N,N-trimethyl-10-oxo-3,5,9-trioxa-4-phosphaheptacos-18-en-1-aminium 4-oxide](../../../ligand/217363/info) | Antagonist | <7XMT> |
| Cryo-EM | Class A | [5HT4R](http://www.uniprot.org/uniprot/Q13639) | [ZM-241385](../../../ligand/4069/info) | Antagonist | <7XT8> |
| Cryo-EM | Class A | [5HT4R](http://www.uniprot.org/uniprot/Q13639) | [CHEMBL5206530](../../../ligand/191721/info) | Antagonist | <7XT9> |
| Cryo-EM | Class A | [5HT4R](http://www.uniprot.org/uniprot/Q13639) | [4-[6-(2-naphthalen-1-ylethoxy)-2,3,4,5-tetrahydro-1H-3-benzazepin-3-ium-3-yl]butanoic acid](../../../ligand/217359/info) | Antagonist | <7XTA> |
| Cryo-EM | Class A | [5HT6R](http://www.uniprot.org/uniprot/P50406) | [L-054,522](../../../ligand/2167/info) | Agonist | <7XTB> |
| Cryo-EM | Class A | [5HT7R](http://www.uniprot.org/uniprot/P34969) | [J-2156](../../../ligand/2070/info) | Agonist | <7XTC> |
| Cryo-EM | Class A | [ACKR3](http://www.uniprot.org/uniprot/P25106) | [5-hydroxytryptamine](../../../ligand/139/info) | Agonist | <7SK9> |
| Cryo-EM | Class A | [ADRB1](http://www.uniprot.org/uniprot/P07700) | [5-hydroxytryptamine](../../../ligand/139/info) | Agonist | <8DCS> |
| Cryo-EM | Class A | [ADRB1](http://www.uniprot.org/uniprot/P07700) | [5-hydroxytryptamine](../../../ligand/139/info) | Agonist | <8DCR> |
| Cryo-EM | Class A | [APJ](http://www.uniprot.org/uniprot/P35414) | [5-hydroxytryptamine](../../../ligand/139/info) | Agonist | <7W0M> |
| X-ray diffraction | Class A | [APJ](http://www.uniprot.org/uniprot/P35414) | [5-CT](../../../ligand/134/info) | Partial agonist | <7SUS> |
| Cryo-EM | Class A | [APJ](http://www.uniprot.org/uniprot/P35414) | [(1R)-4-[7-(3-carboxypropoxy)-6-methylquinolin-8-yl]-1-{[2-(4-hydroxypiperidin-1-yl)-1,3-thiazol-4-yl]methyl}-1,4-diazepan-1-ium](../../../ligand/217346/info) | Agonist | <7W0L> |
| Cryo-EM | Class A | [5HT5A](http://www.uniprot.org/uniprot/P47898) | [4-{[(2S)-3-(tert-butylamino)-2-hydroxypropyl]oxy}-3H-indole-2-carbonitrile](../../../ligand/217348/info) | Agonist | <7UM7> |
| X-ray diffraction | Class A | [5HT5A](http://www.uniprot.org/uniprot/P47898) | [Dobutamine](../../../ligand/217179/info) | Agonist | <7UM4> |
| Cryo-EM | Class A | [5HT5A](http://www.uniprot.org/uniprot/P47898) | [(1R,2S)-N-[4-(2,6-dimethoxyphenyl)-5-(6-methylpyridin-2-yl)-1,2,4-triazol-3-yl]-1-(5-methylpyrimidin-2-yl)-1-oxidanyl-propane-2-sulfonamide](../../../ligand/217340/info) | Agonist | <7UM5> |
| Cryo-EM | Class A | [5HT5A](http://www.uniprot.org/uniprot/P47898) | [(1R,2S)-N-[4-(2,6-dimethoxyphenyl)-5-(6-methylpyridin-2-yl)-1,2,4-triazol-3-yl]-1-(5-methylpyrimidin-2-yl)-1-oxidanyl-propane-2-sulfonamide](../../../ligand/217340/info) | Agonist | <7UM6> |
| Cryo-EM | Class A | [CCKAR](http://www.uniprot.org/uniprot/P32238) | [(1R,2S)-N-[4-(2,6-dimethoxyphenyl)-5-(6-methylpyridin-2-yl)-1,2,4-triazol-3-yl]-1-(5-methylpyrimidin-2-yl)-1-oxidanyl-propane-2-sulfonamide](../../../ligand/217340/info) | Agonist | <7XOV> |
| Cryo-EM | Class A | [MRGRD](http://www.uniprot.org/uniprot/Q8TDS7) | [methylergonovine](../../../ligand/2463/info) | Antagonist | <7Y12> |
| Cryo-EM | Class A | [MRGRD](http://www.uniprot.org/uniprot/Q8TDS7) | [CHEMBL3654198](../../../ligand/126256/info) | Agonist | <7Y14> |
| Cryo-EM | Class A | [5HT2A](http://www.uniprot.org/uniprot/P28223) | [5-CT](../../../ligand/134/info) | Agonist | <7RAN> |
| Cryo-EM | Class A | [GPBAR](http://www.uniprot.org/uniprot/Q8TDU6) | [lisuride](../../../ligand/2284/info) | Agonist | <7XTQ> |
| Cryo-EM | Class A | [DRD1](http://www.uniprot.org/uniprot/P21728) | [SR146131](../../../ligand/3597/info) | Agonist | <7X2C> |
| Cryo-EM | Class A | [GALR2](http://www.uniprot.org/uniprot/O43603) | [β-alanine](../../../ligand/596/info) | Agonist | <7XJL> |
| Cryo-EM | Class A | [HRH2](http://www.uniprot.org/uniprot/P25021) | [β-alanine](../../../ligand/596/info) | Agonist | <7UL3> |
| Cryo-EM | Class A | [NTR1](http://www.uniprot.org/uniprot/P30989) | [3-[(3R)-3-methyl-1,2,3,6-tetrahydropyridin-1-ium-5-yl]-1H-pyrrolo[2,3-b]pyridine](../../../ligand/217339/info) | Agonist | <7UL2> |
| Cryo-EM | Class A | [OPRM](http://www.uniprot.org/uniprot/P42866) | [CHEMBL2181246](../../../ligand/82584/info) | Agonist | <7UL4> |
| X-ray diffraction | Class A | [CNR1](http://www.uniprot.org/uniprot/P21554) | [fenoldopam](../../../ligand/1591/info) | Agonist | <7FEE> |
| Cryo-EM | Class A | [CNR1](http://www.uniprot.org/uniprot/P21554) | [Spexin](../../../ligand/216473/info) | Antagonist | <7WV9> |
| Cryo-EM | Class A | [DRD1](http://www.uniprot.org/uniprot/P21728) | [famotidine](../../../ligand/1584/info) | Inverse agonist | <7F0T> |
| Cryo-EM | Class A | [DRD1](http://www.uniprot.org/uniprot/P21728) | [meclinertant](../../../ligand/2436/info) | Antagonist | <7F1O> |
| Cryo-EM | Class A | [DRD1](http://www.uniprot.org/uniprot/P21728) | [alvimopan](../../../ligand/368/info) | Agonist | <7F1Z> |
| Cryo-EM | Class A | [DRD1](http://www.uniprot.org/uniprot/P21728) | [CP55940](../../../ligand/1157/info) | PAM | <7F23> |
| Cryo-EM | Class A | [DRD1](http://www.uniprot.org/uniprot/P21728) | [6-methyl-3-[(1S)-2-nitro-1-thiophen-2-yl-ethyl]-2-phenyl-1H-indole](../../../ligand/217337/info) | Agonist | <7F24> |
| Cryo-EM | Class A | [DRD1](http://www.uniprot.org/uniprot/P21728) | [CP55940](../../../ligand/1157/info) | PAM | <7X2D> |
| Cryo-EM | Class A | [DRD1](http://www.uniprot.org/uniprot/P21728) | [6-methyl-3-[(1S)-2-nitro-1-thiophen-2-yl-ethyl]-2-phenyl-1H-indole](../../../ligand/217337/info) | Agonist | <7X2F> |
| Cryo-EM | Class A | [OX2R](http://www.uniprot.org/uniprot/O43614) | [dopamine](../../../ligand/1432/info) | Agonist | <7SR8> |
| Cryo-EM | Class A | [SSR2](http://www.uniprot.org/uniprot/P30874) | [dopamine](../../../ligand/1432/info) | Agonist | <7WIG> |
| Cryo-EM | Class A | [OX2R](http://www.uniprot.org/uniprot/O43614) | [dopamine](../../../ligand/1432/info) | Agonist | <7SQO> |
| Cryo-EM | Class A | [ACM4](http://www.uniprot.org/uniprot/P08173) | [dopamine](../../../ligand/1432/info) | Agonist | <7V68> |
| Cryo-EM | Class A | [ACM4](http://www.uniprot.org/uniprot/P08173) | [dopamine](../../../ligand/1432/info) | Agonist | <7V6A> |
| Cryo-EM | Class A | [ADRB3](http://www.uniprot.org/uniprot/O02662) | [TAVAPADON](../../../ligand/131942/info) | Agonist | <7XJH> |
| Cryo-EM | Class A | [ADRB3](http://www.uniprot.org/uniprot/O02662) | [dopamine](../../../ligand/1432/info) | PAM | <7XJI> |
| Cryo-EM | Class A | [OPRM](http://www.uniprot.org/uniprot/P42866) | [mevidalen](../../../ligand/2478/info) | Agonist | <7U2L> |
| Cryo-EM | Class A | [OPRM](http://www.uniprot.org/uniprot/P42866) | [methyl (2R,3S)-3-[(methanesulfonyl)amino]-2-({[(1s,4S)-4-phenylcyclohexyl]oxy}methyl)piperidine-1-carboxylate](../../../ligand/217330/info) | Agonist | <7SCG> |
| Cryo-EM | Class A | [OPRM](http://www.uniprot.org/uniprot/P42866) | [~{N}-[(2~{R})-1-[[(1~{R},3~{S})-3-(aminomethyl)cyclohexyl]methylamino]-3-(1~{H}-indol-3-yl)-1-oxidanylidene-propan-2-yl]spiro[indene-1,4'-piperidine]-1'-carboxamide](../../../ligand/217334/info) | Agonist | <7SBF> |
| X-ray diffraction | Class A | [AA2AR](http://www.uniprot.org/uniprot/P29274) | [methyl (2R,3S)-3-[(methanesulfonyl)amino]-2-({[(1s,4S)-4-phenylcyclohexyl]oxy}methyl)piperidine-1-carboxylate](../../../ligand/217330/info) | Agonist | <7EZC> |
| Cryo-EM | Class A | [ADA2A](http://www.uniprot.org/uniprot/P08913) | [iperoxo](../../../ligand/2020/info) | PAM | <7EJ0> |
| Cryo-EM | Class A | [ADA2A](http://www.uniprot.org/uniprot/P08913) | [LY2119620](../../../ligand/2356/info) | Agonist | <7EJA> |
| Cryo-EM | Class A | [ADA2A](http://www.uniprot.org/uniprot/P08913) | [methyl 4-[4-(3-methyl-2-oxidanylidene-benzimidazol-1-yl)piperidin-1-yl]piperidine-1-carboxylate](../../../ligand/217331/info) | Agonist | <7EJ8> |
| Cryo-EM | Class A | [ADA2A](http://www.uniprot.org/uniprot/P08913) | [LEVISOPRENALINE](../../../ligand/10123/info) | Agonist | <7EJK> |
| Cryo-EM | Class A | [GP183](http://www.uniprot.org/uniprot/P32249) | [3-[3-[2-[[(2~{S})-2-(3-chlorophenyl)-2-oxidanyl-ethyl]amino]ethylamino]phenyl]benzoic acid](../../../ligand/217333/info) | Agonist | <7TUY> |
| Cryo-EM | Class A | [GP183](http://www.uniprot.org/uniprot/P32249) | N-(5-carbamimidamidopentyl)-N-[1-(2-phenylethyl)piperidin-4-yl]propanamide | Agonist | <7TUZ> |
| Cryo-EM | Class A | [GPR88](http://www.uniprot.org/uniprot/Q9GZN0) | [(2E)-N-[(2S)-2-(dimethylamino)-3-(4-hydroxyphenyl)propyl]-3-(naphthalen-1-yl)prop-2-enamide](../../../ligand/217318/info) | Agonist | <7EJX> |
| Cryo-EM | Class A | [S1PR2](http://www.uniprot.org/uniprot/O95136) | [N-[(2S)-2-(dimethylamino)-3-(4-hydroxyphenyl)propyl]-N'-[(2S)-1-(thiophen-3-yl)propan-2-yl]urea](../../../ligand/217317/info) | Agonist | <7T6B> |
| Cryo-EM | Class A | [FPR2](http://www.uniprot.org/uniprot/P25090) | [UK-432,097](../../../ligand/3861/info) | Agonist | <7T6S> |
| Cryo-EM | Class A | [LT4R1](http://www.uniprot.org/uniprot/Q15722) | [(-)-noradrenaline](../../../ligand/2805/info) | Agonist | <7VKT> |
| X-ray diffraction | Class A | [AA2AR](http://www.uniprot.org/uniprot/P29274) | [dexmedetomidine](../../../ligand/1372/info) | Agonist | <7PX4> |
| X-ray diffraction | Class A | [AA2AR](http://www.uniprot.org/uniprot/P29274) | [brimonidine](../../../ligand/713/info) | Agonist | <7PYR> |
| Cryo-EM | Class A | [MTR1A](http://www.uniprot.org/uniprot/P48039) | [oxymetazoline](../../../ligand/2921/info) | Inverse agonist | <7VGY> |
| Cryo-EM | Class A | [MTR1A](http://www.uniprot.org/uniprot/P48039) | [8-[(2E)-3-(4-chlorophenyl)prop-2-enoyl]-3-[(3,4-dichlorophenyl)methyl]-1-oxa-3,8-diazaspiro[4.5]decan-2-one](../../../ligand/217320/info) | Agonist | <7VGZ> |
| Cryo-EM | Class A | [MTR1B](http://www.uniprot.org/uniprot/P49286) | [(2S,4aS,4bS,7R,8S,8aS,9R,10aR)-7-[(2R,3R)-7-hydroxy-3,7-dimethyloctan-2-yl]-4a,7,8-trimethyltetradecahydrophenanthrene-2,9-diol](../../../ligand/217321/info) | Agonist | <7VH0> |
| X-ray diffraction | Class A | [AGTR2](http://www.uniprot.org/uniprot/P50052) | [compound 2 [PMID: 24793972]](../../../ligand/1044/info) | Agonist | <7JNI> |
| Cryo-EM | Class A | [LPAR1](http://www.uniprot.org/uniprot/Q92633) | [sphingosine 1-phosphate](../../../ligand/3579/info) | Agonist | <7TD1> |
| Cryo-EM | Class A | [LPAR1](http://www.uniprot.org/uniprot/Q92633) | [N-(4-chlorophenyl)-N'-[1-methyl-3-oxo-2-phenyl-5-(propan-2-yl)-2,3-dihydro-1H-pyrazol-4-yl]urea](../../../ligand/217300/info) | Agonist | <7TD0> |
| Cryo-EM | Class A | [LPAR1](http://www.uniprot.org/uniprot/Q92633) | [LTB4](../../../ligand/2326/info) | Antagonist | <7TD2> |
| Cryo-EM | Class A | [S1PR1](http://www.uniprot.org/uniprot/P21453) | [Preladenant conjugate PSB-2113](../../../ligand/217306/info) | Antagonist | <7TD3> |
| Cryo-EM | Class A | [S1PR1](http://www.uniprot.org/uniprot/P21453) | Preladenant conjugate PSB-2115 | Agonist | <7TD4> |
| X-ray diffraction | Class A | [5HT2A](http://www.uniprot.org/uniprot/P28223) | [2-iodo-melatonin](../../../ligand/70/info) | Agonist | <7WC4> |
| X-ray diffraction | Class A | [5HT2A](http://www.uniprot.org/uniprot/P28223) | [ramelteon](../../../ligand/3224/info) | Agonist | <7WC6> |
| X-ray diffraction | Class A | [5HT2A](http://www.uniprot.org/uniprot/P28223) | [ramelteon](../../../ligand/3224/info) | Antagonist | <7WC8> |
| X-ray diffraction | Class A | [5HT2A](http://www.uniprot.org/uniprot/P28223) | [olodanrigan](../../../ligand/2866/info) | Agonist | <7WC5> |
| X-ray diffraction | Class A | [5HT2A](http://www.uniprot.org/uniprot/P28223) | [lysophosphatidic acid](../../../ligand/217305/info) | Agonist | <7WC7> |
| X-ray diffraction | Class A | [5HT2A](http://www.uniprot.org/uniprot/P28223) | [lysophosphatidic acid](../../../ligand/217305/info) | Agonist | <7WC9> |
| X-ray diffraction | Class A | [GHSR](http://www.uniprot.org/uniprot/Q92847) | [lysophosphatidic acid](../../../ligand/217305/info) | Agonist | <7F83> |
| X-ray diffraction | Class A | [ADA1B](http://www.uniprot.org/uniprot/P35368) | [sphingosine 1-phosphate](../../../ligand/3579/info) | Agonist | <7B6W> |
| Cryo-EM | Class A | [S1PR1](http://www.uniprot.org/uniprot/P21453) | [siponimod](../../../ligand/3539/info) | Agonist | <7EO2> |
| Cryo-EM | Class A | [S1PR1](http://www.uniprot.org/uniprot/P21453) | [5-hydroxytryptamine](../../../ligand/139/info) | Agonist | <7WF7> |
| Cryo-EM | Class A | [S1PR1](http://www.uniprot.org/uniprot/P21453) | [Lysergide](../../../ligand/2386/info) | Agonist | <7EO4> |
| Cryo-EM | Class A | [GP139](http://www.uniprot.org/uniprot/Q6DWJ6) | [LUMATEPERONE](../../../ligand/112295/info) | Agonist | <7VUH> |
| Cryo-EM | Class A | [GP139](http://www.uniprot.org/uniprot/Q6DWJ6) | [psilocin](../../../ligand/3157/info) | Agonist | <7VUJ> |
| Cryo-EM | Class A | [GP139](http://www.uniprot.org/uniprot/Q6DWJ6) | [lisuride](../../../ligand/2284/info) | Agonist | <7VUG> |
| Cryo-EM | Class A | [GP139](http://www.uniprot.org/uniprot/Q6DWJ6) | [IHCH-7086](../../../ligand/217050/info) | Inverse agonist | <7VUI> |
| X-ray diffraction | Class A | [5HT2A](http://www.uniprot.org/uniprot/P28223) | [PF-05190457](../../../ligand/3004/info) | Inverse agonist | <7VOD> |
| X-ray diffraction | Class A | [5HT2A](http://www.uniprot.org/uniprot/P28223) | [(+)-cyclazosin](../../../ligand/1247/info) | Agonist | <7VOE> |
| Cryo-EM | Class A | [GHSR](http://www.uniprot.org/uniprot/Q92847) | [(S)-FTY720-phosphate](../../../ligand/3526/info) | Agonist | <7NA8> |
| Cryo-EM | Class A | [MRGX2](http://www.uniprot.org/uniprot/Q96LB1) | [sphingosine 1-phosphate](../../../ligand/3579/info) | Agonist | <7VV6> |
| Cryo-EM | Class A | [MRGX2](http://www.uniprot.org/uniprot/Q96LB1) | [siponimod](../../../ligand/3539/info) | Agonist | <7VDH> |
| Cryo-EM | Class A | [MRGX2](http://www.uniprot.org/uniprot/Q96LB1) | [JNJ-63533054](../../../ligand/2109/info) | Agonist | <7VV5> |
| X-ray diffraction | Class A | [CNR1](http://www.uniprot.org/uniprot/P21554) | [JNJ-63533054](../../../ligand/2109/info) | Agonist | <7V3Z> |
| Cryo-EM | Class A | [ADRB1](http://www.uniprot.org/uniprot/P07700) | [JNJ-63533054](../../../ligand/2109/info) | Agonist | <7S0G> |
| Cryo-EM | Class A | [ADRB1](http://www.uniprot.org/uniprot/P07700) | [JNJ-63533054](../../../ligand/2109/info) | Partial agonist | <7S0F> |
| Cryo-EM | Class A | [MRGX2](http://www.uniprot.org/uniprot/Q96LB1) | [cariprazine](../../../ligand/786/info) | Partial agonist | <7S8N> |
| Cryo-EM | Class A | [MRGX2](http://www.uniprot.org/uniprot/Q96LB1) | [aripiprazole](../../../ligand/467/info) | Agonist | <7S8O> |
| Cryo-EM | Class A | [MRGX4](http://www.uniprot.org/uniprot/Q96LA9) | [ibutamoren](../../../ligand/1932/info) | Agonist | <7S8P> |
| Cryo-EM | Class A | [MC4R](http://www.uniprot.org/uniprot/P32245) | [compound 48/80](../../../ligand/1082/info) | Agonist | <7F58> |
| X-ray diffraction | Class A | [CCKAR](http://www.uniprot.org/uniprot/P32238) | [compound 48/80](../../../ligand/1082/info) | Agonist | <7F8U> |
| X-ray diffraction | Class A | [CCKAR](http://www.uniprot.org/uniprot/P32238) | [compound 48/80](../../../ligand/1082/info) | Agonist | <7F8Y> |
| X-ray diffraction | Class A | [ACM1](http://www.uniprot.org/uniprot/P11229) | [CP55940](../../../ligand/1157/info) | Agonist | <6ZG9> |
| X-ray diffraction | Class A | [ACM1](http://www.uniprot.org/uniprot/P11229) | [LEVISOPRENALINE](../../../ligand/10123/info) | Agonist | <6ZFZ> |
| X-ray diffraction | Class A | [ACM1](http://www.uniprot.org/uniprot/P11229) | [LEVISOPRENALINE](../../../ligand/10123/info) | Agonist | <6ZG4> |
| Cryo-EM | Class A | [S1PR1](http://www.uniprot.org/uniprot/P21453) | [(R)-ZINC-3573](../../../ligand/3359/info) | Agonist | <7EVZ> |
| Cryo-EM | Class A | [S1PR1](http://www.uniprot.org/uniprot/P21453) | [(R)-ZINC-3573](../../../ligand/3359/info) | Agonist | <7EW7> |
| Cryo-EM | Class A | [S1PR1](http://www.uniprot.org/uniprot/P21453) | [N-[(1r,3R,5S,7R)-3,5-dimethyltricyclo[3.3.1.1~3,7~]decane-1-carbonyl]-D-phenylalanine](../../../ligand/217291/info) | Agonist | <7EVY> |
| Cryo-EM | Class A | [S1PR1](http://www.uniprot.org/uniprot/P21453) | [THIQ](../../../ligand/3735/info) | Antagonist | <7EW0> |
| Cryo-EM | Class A | [S1PR3](http://www.uniprot.org/uniprot/Q99500) | [lintitript](../../../ligand/2281/info) | Antagonist | <7EW2> |
| Cryo-EM | Class A | [S1PR3](http://www.uniprot.org/uniprot/Q99500) | [DEVAZEPIDE](../../../ligand/205862/info) | Agonist | <7EW4> |
| Cryo-EM | Class A | [S1PR5](http://www.uniprot.org/uniprot/Q9H228) | [7-fluoro-5-methyl-3-[1-(oxan-4-yl)piperidin-4-yl]-1H-benzimidazol-2-one](../../../ligand/217288/info) | Agonist | <7EW1> |
| Cryo-EM | Class A | [AA1R](http://www.uniprot.org/uniprot/P30542) | [CHEMBL3354065](../../../ligand/115259/info) | Agonist | <7LD4> |
| Cryo-EM | Class A | [AA1R](http://www.uniprot.org/uniprot/P30542) | [Ethyl (4S)-4-[4-[(1-methylcyclobutyl)carbamoyl]piperidin-1-yl]azepane-1-carboxylate](../../../ligand/217287/info) | Agonist | <7LD3> |
| MicroED | Class A | [AA2AR](http://www.uniprot.org/uniprot/P29274) | [cenerimod](../../../ligand/863/info) | Agonist | <7RM5> |
| X-ray diffraction | Class A | [PD2R2](http://www.uniprot.org/uniprot/Q9Y5Y4) | [SEW2871](../../../ligand/3525/info) | Agonist | <7M8W> |
| Cryo-EM | Class A | [MTR1A](http://www.uniprot.org/uniprot/P48039) | [siponimod](../../../ligand/3539/info) | Agonist | <7DB6> |
| Cryo-EM | Class A | [5HT1F](http://www.uniprot.org/uniprot/P30939) | [ozanimod](../../../ligand/2924/info) | Agonist | <7EXD> |
| Cryo-EM | Class A | [ADRB3](http://www.uniprot.org/uniprot/O02662) | [(S)-FTY720-phosphate](../../../ligand/3526/info) | Agonist | <7DH5> |
| Cryo-EM | Class A | [OPSD](http://www.uniprot.org/uniprot/P02699) | [CYM-5541](../../../ligand/1267/info) | Agonist | <7MT8> |
| Cryo-EM | Class A | [OPSD](http://www.uniprot.org/uniprot/P02699) | [siponimod](../../../ligand/3539/info) | Agonist | <7MTA> |
| Cryo-EM | Class A | [OPSD](http://www.uniprot.org/uniprot/P02699) | [adenosine](../../../ligand/280/info) | Ago-PAM | <7MT9> |
| Cryo-EM | Class A | [OPSD](http://www.uniprot.org/uniprot/P02699) | [{2-Amino-4-[3,5-bis(trifluoromethyl)phenyl]thiophen-3-yl}(4-chlorophenyl)methanone](../../../ligand/217281/info) | Agonist | <7MTB> |
| X-ray diffraction | Class A | [S1PR3](http://www.uniprot.org/uniprot/Q99500) | [adenosine](../../../ligand/280/info) | Antagonist | <7C4S> |
| Cryo-EM | Class A | [PE2R2](http://www.uniprot.org/uniprot/P43116) | [ZM-241385](../../../ligand/4069/info) | Antagonist | <7CX2> |
| Cryo-EM | Class A | [PE2R2](http://www.uniprot.org/uniprot/P43116) | [15(R)-15-methyl-PGD2](../../../ligand/22/info) | Agonist | <7CX4> |
| Cryo-EM | Class A | [PE2R2](http://www.uniprot.org/uniprot/P43116) | [ramelteon](../../../ligand/3224/info) | Agonist | <7CX3> |
| Cryo-EM | Class A | [5HT1D](http://www.uniprot.org/uniprot/P28221) | [lasmiditan](../../../ligand/2241/info) | Agonist | [7.00E+32](7E32) |
| Cryo-EM | Class A | [5HT1A](http://www.uniprot.org/uniprot/P08908) | [mirabegron](../../../ligand/2493/info) | Agonist | <7E2Y> |
| Cryo-EM | Class A | [5HT1A](http://www.uniprot.org/uniprot/P08908) | [Retinal (all-trans)](../../../ligand/217173/info) | Agonist | <7E2Z> |
| Cryo-EM | Class A | [5HT1E](http://www.uniprot.org/uniprot/P28566) | [Retinal (all-trans)](../../../ligand/217173/info) | Agonist | [7.00E+33](7E33) |
| X-ray diffraction | Class A | [DRD1](http://www.uniprot.org/uniprot/P21728) | [Retinal (all-trans)](../../../ligand/217173/info) | Agonist | <7JOZ> |
| X-ray diffraction | Class A | [AA2AR](http://www.uniprot.org/uniprot/P29274) | [Retinal (all-trans)](../../../ligand/217173/info) | Agonist | <7ARO> |
| Cryo-EM | Class A | [HRH1](http://www.uniprot.org/uniprot/P35367) | [sphingosine 1-phosphate](../../../ligand/3579/info) | Agonist | <7DFL> |
| Cryo-EM | Class A | [DRD3](http://www.uniprot.org/uniprot/P35462) | [PGE2](../../../ligand/3021/info) | Agonist | <7CMU> |
| Cryo-EM | Class A | [DRD3](http://www.uniprot.org/uniprot/P35462) | [evatanepag](../../../ligand/1569/info) | Agonist | <7CMV> |
| Cryo-EM | Class A | [DRD1](http://www.uniprot.org/uniprot/P21728) | [taprenepag](../../../ligand/3684/info) | Agonist | <7CKW> |
| Cryo-EM | Class A | [DRD1](http://www.uniprot.org/uniprot/P21728) | [5-hydroxytryptamine](../../../ligand/139/info) | Agonist | <7CKY> |
| Cryo-EM | Class A | [DRD1](http://www.uniprot.org/uniprot/P21728) | [5-hydroxytryptamine](../../../ligand/139/info) | Partial agonist | <7CRH> |
| Cryo-EM | Class A | [DRD1](http://www.uniprot.org/uniprot/P21728) | [aripiprazole](../../../ligand/467/info) | Agonist | <7LJC> |
| Cryo-EM | Class A | [DRD1](http://www.uniprot.org/uniprot/P21728) | [BRL-54443](../../../ligand/717/info) | Agonist | <7CKX> |
| Cryo-EM | Class A | [DRD1](http://www.uniprot.org/uniprot/P21728) | [CHEMBL4453318](../../../ligand/170233/info) | Partial agonist | <7CKZ> |
| Cryo-EM | Class A | [DRD1](http://www.uniprot.org/uniprot/P21728) | [CHEMBL124345](../../../ligand/16568/info) | Agonist | <7LJD> |
| Cryo-EM | Class A | [DRD1](http://www.uniprot.org/uniprot/P21728) | [histamine](../../../ligand/1893/info) | Agonist | <7JVP> |
| Cryo-EM | Class A | [DRD1](http://www.uniprot.org/uniprot/P21728) | [pramipexole](../../../ligand/3098/info) | Agonist | <7JV5> |
| Cryo-EM | Class A | [DRD1](http://www.uniprot.org/uniprot/P21728) | [CHEMBL70565](../../../ligand/202444/info) | Agonist | <7JVQ> |
| Cryo-EM | Class A | [DRD2](http://www.uniprot.org/uniprot/P14416) | [fenoldopam](../../../ligand/1591/info) | Agonist | <7JVR> |
| X-ray diffraction | Class A | [LT4R1](http://www.uniprot.org/uniprot/Q15722) | [CHEMBL3697578](../../../ligand/131902/info) | Agonist | <7K15> |
| X-ray diffraction | Class A | [NTR1](http://www.uniprot.org/uniprot/P20789) | [CHEMBL1416789](../../../ligand/32846/info) | Agonist | <6Z4Q> |
| X-ray diffraction | Class A | [NTR1](http://www.uniprot.org/uniprot/P20789) | [CHEMBL1160787](../../../ligand/10128/info) | PAM | <6Z4S> |
| X-ray diffraction | Class A | [NTR1](http://www.uniprot.org/uniprot/P20789) | [mevidalen](../../../ligand/2478/info) | Agonist | <6ZA8> |
| X-ray diffraction | Class A | [NTR1](http://www.uniprot.org/uniprot/P20789) | [A77636](../../../ligand/206/info) | Agonist | <6Z8N> |
| X-ray diffraction | Class A | [NTR1](http://www.uniprot.org/uniprot/P20789) | [dopamine](../../../ligand/1432/info) | PAM | <6ZIN> |
| Cryo-EM | Class A | [OX2R](http://www.uniprot.org/uniprot/O43614) | [mevidalen](../../../ligand/2478/info) | Agonist | <7L1V> |
| X-ray diffraction | Class A | [NPY2R](http://www.uniprot.org/uniprot/P49146) | [dopamine](../../../ligand/1432/info) | PAM | <7DDZ> |
| X-ray diffraction | Class A | [DRD2](http://www.uniprot.org/uniprot/P14416) | [mevidalen](../../../ligand/2478/info) | Agonist | <7DFP> |
| Cryo-EM | Class A | [ADRB2](http://www.uniprot.org/uniprot/P07550) | [39YLC3L0ZU](../../../ligand/217276/info) | Agonist | <7DHR> |
| Cryo-EM | Class A | [ADRB2](http://www.uniprot.org/uniprot/P07550) | [CHEMBL1160787](../../../ligand/10128/info) | Agonist | <7DHI> |
| X-ray diffraction | Class A | [ADRB1](http://www.uniprot.org/uniprot/P08588) | [apomorphine](../../../ligand/441/info) | Agonist | <7BU6> |
| X-ray diffraction | Class A | [ADRB1](http://www.uniprot.org/uniprot/P08588) | [bromocriptine](../../../ligand/719/info) | Antagonist | <7BVQ> |
| X-ray diffraction | Class A | [ADRB1](http://www.uniprot.org/uniprot/P08588) | [N-(tert-butylsulfonyl)-4-fluoro-2-{(3S,4R)-4-hydroxy-3-[(pyridin-2-yl)methyl]-3,4-dihydro-2H-1-benzopyran-7-yl}benzamide](../../../ligand/217275/info) | Inverse agonist | <7BTS> |
| X-ray diffraction | Class A | [ADRB1](http://www.uniprot.org/uniprot/P08588) | [SR142948A](../../../ligand/3595/info) | Inverse agonist | <7BU7> |
| X-ray diffraction | Class A | [AA2AR](http://www.uniprot.org/uniprot/P29274) | [meclinertant](../../../ligand/2436/info) | Partial agonist | <6LPJ> |
| X-ray diffraction | Class A | [AA2AR](http://www.uniprot.org/uniprot/P29274) | [CHEMBL508044](../../../ligand/188342/info) | Agonist | <6LPL> |
| X-ray diffraction | Class A | [AA2AR](http://www.uniprot.org/uniprot/P29274) | [(2~{S})-4-methyl-2-[(1-quinolin-8-ylsulfonylindol-3-yl)carbonylamino]pentanoic acid](../../../ligand/217273/info) | Inverse agonist | <6LPK> |
| X-ray diffraction | Class A | [AA2AR](http://www.uniprot.org/uniprot/P29274) | [meclinertant](../../../ligand/2436/info) | Agonist | <6WQA> |
| Cryo-EM | Class A | [PE2R4](http://www.uniprot.org/uniprot/P35408) | [compound 1 [PMID: 33547286]](../../../ligand/1003/info) | Antagonist | <7D7M> |
| X-ray diffraction | Class A | [GNRHR](http://www.uniprot.org/uniprot/P30968) | [CHEMBL1823578](../../../ligand/64708/info) | Antagonist | <7BR3> |
| X-ray diffraction | Class A | [5HT2A](http://www.uniprot.org/uniprot/P28223) | [spiperone](../../../ligand/3583/info) | Agonist | <6WGT> |
| Cryo-EM | Class A | [5HT2A](http://www.uniprot.org/uniprot/P28223) | [LEVISOPRENALINE](../../../ligand/10123/info) | Agonist | <6WHA> |
| X-ray diffraction | Class A | [5HT2A](http://www.uniprot.org/uniprot/P28223) | [levosalbutamol](../../../ligand/2275/info) | Agonist | <6WH4> |
| X-ray diffraction | Class A | [AA2AR](http://www.uniprot.org/uniprot/P29274) | [Noradrenaline](../../../ligand/217270/info) | Inverse agonist | <6ZDR> |
| X-ray diffraction | Class A | [AA2AR](http://www.uniprot.org/uniprot/P29274) | [Carazolol](../../../ligand/217176/info) | Agonist | <6ZDV> |
| X-ray diffraction | Class A | [SUCR1](http://www.uniprot.org/uniprot/Q6IYF9) | [(-)-adrenaline](../../../ligand/287/info) | Agonist | <6Z10> |
| Cryo-EM | Class A | [GPBAR](http://www.uniprot.org/uniprot/Q8TDU6) | [CHEMBL1615159](../../../ligand/54654/info) | Antagonist | <7CFM> |
| Cryo-EM | Class A | [GPBAR](http://www.uniprot.org/uniprot/Q8TDU6) | [ZM-241385](../../../ligand/4069/info) | Antagonist | <7CFN> |
| Cryo-EM | Class A | [ADRB1](http://www.uniprot.org/uniprot/P07700) | [ZM-241385](../../../ligand/4069/info) | Antagonist | <7JJO> |
| X-ray diffraction | Class A | [CXCR2](http://www.uniprot.org/uniprot/P25025) | [ZM-241385](../../../ligand/4069/info) | Antagonist | <6LFL> |
| X-ray diffraction | Class A | [GHSR](http://www.uniprot.org/uniprot/Q92847) | [ZM-241385](../../../ligand/4069/info) | Agonist | <6KO5> |
| Cryo-EM | Class A | [ADRB2](http://www.uniprot.org/uniprot/P07550) | [PGE2](../../../ligand/3021/info) | Antagonist | <7BZ2> |
| X-ray diffraction | Class A | [OXYR](http://www.uniprot.org/uniprot/P30559) | [elagolix](../../../ligand/1510/info) | Agonist | <6TPK> |
| X-ray diffraction | Class A | [5HT1B](http://www.uniprot.org/uniprot/P28222) | [Lysergide](../../../ligand/2386/info) | Agonist | <7C61> |
| X-ray diffraction | Class A | [AA2AR](http://www.uniprot.org/uniprot/P29274) | [CHEMBL5184791](../../../ligand/190269/info) | Inverse agonist | <6S0L> |
| X-ray diffraction | Class A | [AA2AR](http://www.uniprot.org/uniprot/P29274) | [CHEMBL428892](../../../ligand/166481/info) | Antagonist | <6S0Q> |
| X-ray diffraction | Class A | [OX1R](http://www.uniprot.org/uniprot/O43613) | [CHEMBL2030687](../../../ligand/74354/info) | Antagonist | <6V9S> |
| X-ray diffraction | Class A | [ACM1](http://www.uniprot.org/uniprot/P11229) | [2-Methyl-3-(4-methylthiazol-2-yl)-4-oxo-6-propyl-4H-chromen-7-yl acetate](../../../ligand/217267/info) | Antagonist | <6WJC> |
| X-ray diffraction | Class A | [OPSD](http://www.uniprot.org/uniprot/P02699) | [CHEMBL4751488](../../../ligand/179768/info) | Agonist | <6PGS> |
| X-ray diffraction | Class A | [OPSD](http://www.uniprot.org/uniprot/P02699) | [CHEMBL2331646](../../../ligand/86847/info) | Agonist | <6PH7> |
| X-ray diffraction | Class A | [OPSD](http://www.uniprot.org/uniprot/P02699) | [INT-777](../../../ligand/217266/info) | PAM | <6PEL> |
| Cryo-EM | Class A | [ADRB1](http://www.uniprot.org/uniprot/P07700) | [INT-777](../../../ligand/217266/info) | Agonist | <6TKO> |
| Cryo-EM | Class A | [DRD2](http://www.uniprot.org/uniprot/P14416) | [LEVISOPRENALINE](../../../ligand/10123/info) | Antagonist | <6VMS> |
| Cryo-EM | Class A | [ADA2B](http://www.uniprot.org/uniprot/P18089) | [4-[[3,4-Dioxo-2-[[(1R)-1-(4-propan-2-ylfuran-2-yl)propyl]amino]cyclobuten-1-yl]amino]-3-hydroxy-N,N-dimethylpyridine-2-carboxamide](../../../ligand/217263/info) | Antagonist | <6K41> |
| Cryo-EM | Class A | [ADA2B](http://www.uniprot.org/uniprot/P18089) | [CHEMBL1956994](../../../ligand/70918/info) | Agonist | <6K42> |
| X-ray diffraction | Class A | [ADRB2](http://www.uniprot.org/uniprot/P07550) | [arformoterol](../../../ligand/462/info) | Antagonist | <6OBA> |
| X-ray diffraction | Class A | [OPRK](http://www.uniprot.org/uniprot/P41145) | [retosiban](../../../ligand/3257/info) | Antagonist | <6VI4> |
| X-ray diffraction | Class A | [DRD2](http://www.uniprot.org/uniprot/P14416) | [ergotamine](../../../ligand/1545/info) | Antagonist | <6LUQ> |
| Cryo-EM | Class A | [ACM2](http://www.uniprot.org/uniprot/P08172) | [ZM-241385](../../../ligand/4069/info) | Antagonist | <6U1N> |
| X-ray diffraction | Class A | [GPR52](http://www.uniprot.org/uniprot/Q9Y2T5) | [ZM-241385](../../../ligand/4069/info) | Antagonist | <6LI0> |
| Cryo-EM | Class A | [CNR1](http://www.uniprot.org/uniprot/P21554) | [JH112](../../../ligand/2075/info) | Antagonist | <6KPG> |
| X-ray diffraction | Class A | [CNR2](http://www.uniprot.org/uniprot/P34972) | [ENDO-ATROPINE](../../../ligand/92815/info) | unknown | <6KPC> |
| Cryo-EM | Class A | [CNR2](http://www.uniprot.org/uniprot/P34972) | [Geraniol](../../../ligand/217254/info) | unknown | <6KPF> |
| Cryo-EM | Class A | [CNR2](http://www.uniprot.org/uniprot/P34972) | [Nerol](../../../ligand/217255/info) | unknown | <6PT0> |
| X-ray diffraction | Class A | [OPSD](http://www.uniprot.org/uniprot/P02699) | [Citronellol](../../../ligand/217253/info) | Agonist | <6NWE> |
| X-ray diffraction | Class A | [OX1R](http://www.uniprot.org/uniprot/O43613) | [arformoterol](../../../ligand/462/info) | Agonist | <6TOS> |
| X-ray diffraction | Class A | [OX1R](http://www.uniprot.org/uniprot/O43613) | [bromocriptine](../../../ligand/719/info) | Agonist | <6TOT> |
| X-ray diffraction | Class A | [OX1R](http://www.uniprot.org/uniprot/O43613) | [dexmedetomidine](../../../ligand/1372/info) | Agonist | <6TOD> |
| X-ray diffraction | Class A | [OX1R](http://www.uniprot.org/uniprot/O43613) | [dexmedetomidine](../../../ligand/1372/info) | Antagonist | <6TP4> |
| X-ray diffraction | Class A | [OX1R](http://www.uniprot.org/uniprot/O43613) | [CHEMBL1160734](../../../ligand/10125/info) | NAM | <6TQ4> |
| X-ray diffraction | Class A | [OX1R](http://www.uniprot.org/uniprot/O43613) | [AS408](../../../ligand/482/info) | Antagonist | <6TQ7> |
| X-ray diffraction | Class A | [OX1R](http://www.uniprot.org/uniprot/O43613) | [JDTic](../../../ligand/2074/info) | Antagonist | <6TO7> |
| X-ray diffraction | Class A | [OX1R](http://www.uniprot.org/uniprot/O43613) | [haloperidol](../../../ligand/1872/info) | Agonist | <6TP3> |
| X-ray diffraction | Class A | [OX1R](http://www.uniprot.org/uniprot/O43613) | [LY2119620](../../../ligand/2356/info) | Agonist | <6TP6> |
| X-ray diffraction | Class A | [OX1R](http://www.uniprot.org/uniprot/O43613) | [derivative 17 [Nakahata et al., 2018]](../../../ligand/1339/info) | Agonist | <6TQ6> |
| X-ray diffraction | Class A | [OX1R](http://www.uniprot.org/uniprot/O43613) | [AM841](../../../ligand/385/info) | Agonist | <6TQ9> |
| X-ray diffraction | Class A | [OX2R](http://www.uniprot.org/uniprot/O43614) | [7-[(6aR,9R,10aR)-1-Hydroxy-9-(hydroxymethyl)-6,6-dimethyl-6a,7,8,9,10,10a-hexahydro-6H-benzo[c]chromen-3-yl]-7-methyloctanenitrile](../../../ligand/217247/info) | Agonist | <6TPG> |
| X-ray diffraction | Class A | [OX2R](http://www.uniprot.org/uniprot/O43614) | [7-[(6aR,9R,10aR)-1-Hydroxy-9-(hydroxymethyl)-6,6-dimethyl-6a,7,8,9,10,10a-hexahydro-6H-benzo[c]chromen-3-yl]-7-methyloctanenitrile](../../../ligand/217247/info) | Agonist | <6TPN> |
| X-ray diffraction | Class A | [OX2R](http://www.uniprot.org/uniprot/O43614) | [WIN55212-2](../../../ligand/4007/info) | unknown | <6TPJ> |
| X-ray diffraction | Class A | [ACM5](http://www.uniprot.org/uniprot/P08912) | [B-Octylglucoside](../../../ligand/217185/info) | Antagonist | <6OL9> |
| X-ray diffraction | Class A | [CLTR2](http://www.uniprot.org/uniprot/Q9NS75) | [CHEMBL2413522](../../../ligand/91449/info) | Antagonist | <6RZ6> |
| X-ray diffraction | Class A | [CLTR2](http://www.uniprot.org/uniprot/Q9NS75) | [lemborexant](../../../ligand/2250/info) | Antagonist | <6RZ8> |
| X-ray diffraction | Class A | [CLTR2](http://www.uniprot.org/uniprot/Q9NS75) | [EMPA](../../../ligand/1519/info) | Antagonist | <6RZ7> |
| X-ray diffraction | Class A | [CLTR2](http://www.uniprot.org/uniprot/Q9NS75) | [ACT-462206](../../../ligand/271/info) | Antagonist | <6RZ9> |
| X-ray diffraction | Class A | [OPRD](http://www.uniprot.org/uniprot/P41143) | [CHEMBL4590386](../../../ligand/175456/info) | Antagonist | <6PT3> |
| X-ray diffraction | Class A | [ADA2A](http://www.uniprot.org/uniprot/P08913) | [SB-334867](../../../ligand/3450/info) | Antagonist | <6KUX> |
| X-ray diffraction | Class A | [ADA2A](http://www.uniprot.org/uniprot/P08913) | [suvorexant](../../../ligand/3644/info) | Antagonist | <6KUY> |
| X-ray diffraction | Class A | [ADA2C](http://www.uniprot.org/uniprot/P18825) | [daridorexant](../../../ligand/1291/info) | Antagonist | <6KUW> |
| X-ray diffraction | Class A | [DRD4](http://www.uniprot.org/uniprot/P51436) | [filorexant](../../../ligand/1600/info) | Antagonist | <6IQL> |
| Cryo-EM | Class A | [ADRB2](http://www.uniprot.org/uniprot/P07550) | [CHEMBL4592653](../../../ligand/175566/info) | Antagonist | <6NI3> |
| X-ray diffraction | Class A | [AA2AR](http://www.uniprot.org/uniprot/P29274) | [SB-408124](../../../ligand/3453/info) | Antagonist | <6PS7> |
| X-ray diffraction | Class A | [ADRB2](http://www.uniprot.org/uniprot/P07550) | [EMPA](../../../ligand/1519/info) | Antagonist | <6PRZ> |
| X-ray diffraction | Class A | [ADRB2](http://www.uniprot.org/uniprot/P07550) | [HTL6641](../../../ligand/1906/info) | Antagonist | <6PS1> |
| X-ray diffraction | Class A | [ADRB2](http://www.uniprot.org/uniprot/P07550) | [suvorexant](../../../ligand/3644/info) | Inverse agonist | <6PS3> |
| X-ray diffraction | Class A | [ADRB2](http://www.uniprot.org/uniprot/P07550) | [tiotropium](../../../ligand/3752/info) | Antagonist | <6PS5> |
| X-ray diffraction | Class A | [ADRB2](http://www.uniprot.org/uniprot/P07550) | [ONO-2570366](../../../ligand/2875/info) | Antagonist | <6PS0> |
| X-ray diffraction | Class A | [ADRB2](http://www.uniprot.org/uniprot/P07550) | [ONO-2080365](../../../ligand/217243/info) | Antagonist | <6PS2> |
| X-ray diffraction | Class A | [ADRB2](http://www.uniprot.org/uniprot/P07550) | [ONO-2570366](../../../ligand/2875/info) | Antagonist | <6PS4> |
| X-ray diffraction | Class A | [ADRB2](http://www.uniprot.org/uniprot/P07550) | [ONO-2770372](../../../ligand/217244/info) | Agonist | <6PS6> |
| X-ray diffraction | Class A | [MTR1A](http://www.uniprot.org/uniprot/P48039) | [DPI-287](../../../ligand/217242/info) | Antagonist | <6PS8> |
| X-ray diffraction | Class A | [AA2AR](http://www.uniprot.org/uniprot/P29274) | [RSC](../../../ligand/217239/info) | Partial agonist | <6JZH> |
| X-ray diffraction | Class A | [CLTR1](http://www.uniprot.org/uniprot/Q9Y271) | [(2~{S})-4-fluoranyl-2-(1~{H}-imidazol-5-yl)-1-propan-2-yl-2,3-dihydroindole](../../../ligand/217240/info) | Antagonist | <6RZ5> |
| X-ray diffraction | Class A | [CLTR1](http://www.uniprot.org/uniprot/Q9Y271) | [(8Ar,12aS,13aR)-12-ethylsulfonyl-3-methoxy-5,6,8,8a,9,10,11,12a,13,13a-decahydroisoquinolino[2,1-g][1,6]naphthyridine](../../../ligand/217238/info) | Antagonist | <6RZ4> |
| X-ray diffraction | Class A | [CNR1](http://www.uniprot.org/uniprot/P21554) | [L745870](../../../ligand/2204/info) | Agonist | <6KQI> |
| X-ray diffraction | Class A | [CCR7](http://www.uniprot.org/uniprot/P32248) | [CHEMBL1615159](../../../ligand/54654/info) | Antagonist | <6QZH> |
| X-ray diffraction | Class A | [SUCR1](http://www.uniprot.org/uniprot/Q6IYF9) | [ZM-241385](../../../ligand/4069/info) | Antagonist | <6RNK> |
| Cryo-EM | Class A | [OPSD](http://www.uniprot.org/uniprot/P02699) | [CHEMBL1160734](../../../ligand/10125/info) | Antagonist | <6OYA> |
| Cryo-EM | Class A | [OPSD](http://www.uniprot.org/uniprot/P02699) | [timolol](../../../ligand/3749/info) | Antagonist | <6OY9> |
| Cryo-EM | Class A | [OPSD](http://www.uniprot.org/uniprot/P02699) | [CHEMBL3799125](../../../ligand/139303/info) | Antagonist | <6QNO> |
| X-ray diffraction | Class A | [B1B1U5](http://www.uniprot.org/uniprot/B1B1U5) | [propranolol](../../../ligand/3126/info) | Antagonist | <6I9K> |
| X-ray diffraction | Class A | [AA2AR](http://www.uniprot.org/uniprot/P29274) | [carazolol](../../../ligand/779/info) | Antagonist | <6GT3> |
| X-ray diffraction | Class A | [ADRB2](http://www.uniprot.org/uniprot/P07550) | [CHEMBL1160734](../../../ligand/10125/info) | Antagonist | <6N48> |
| X-ray diffraction | Class A | [ADRB2](http://www.uniprot.org/uniprot/P07550) | [ICI 118551](../../../ligand/1935/info) | Antagonist | [6.00E+67](6E67) |
| Cryo-EM | Class A | [ACM1](http://www.uniprot.org/uniprot/P11229) | [timolol](../../../ligand/3749/info) | Agonist | <6OIJ> |
| Cryo-EM | Class A | [ACM2](http://www.uniprot.org/uniprot/P08172) | [CHEMBL15060](../../../ligand/42959/info) | Antagonist | <6OIK> |
| X-ray diffraction | Class A | [AA2AR](http://www.uniprot.org/uniprot/P29274) | [ZM-241385](../../../ligand/4069/info) | Antagonist | <6MH8> |
| X-ray diffraction | Class A | [MTR1A](http://www.uniprot.org/uniprot/P48039) | [zafirlukast](../../../ligand/4056/info) | Antagonist | <6ME2> |
| X-ray diffraction | Class A | [MTR1A](http://www.uniprot.org/uniprot/P48039) | [pranlukast](../../../ligand/3101/info) | Agonist | <6ME3> |
| X-ray diffraction | Class A | [MTR1A](http://www.uniprot.org/uniprot/P48039) | [CP55940](../../../ligand/1157/info) | NAM | <6ME4> |
| X-ray diffraction | Class A | [MTR1A](http://www.uniprot.org/uniprot/P48039) | [Org27569](../../../ligand/2906/info) | Antagonist | <6ME5> |
| X-ray diffraction | Class A | [MTR1B](http://www.uniprot.org/uniprot/P49286) | [SCHEMBL13407377](../../../ligand/217237/info) | Antagonist | <6ME6> |
| X-ray diffraction | Class A | [MTR1B](http://www.uniprot.org/uniprot/P49286) | [NF-56-EJ40](../../../ligand/2763/info) | Agonist | <6ME8> |
| X-ray diffraction | Class A | [MTR1B](http://www.uniprot.org/uniprot/P49286) | [Retinal (all-trans)](../../../ligand/217173/info) | Agonist | <6ME7> |
| X-ray diffraction | Class A | [MTR1B](http://www.uniprot.org/uniprot/P49286) | [Retinal (all-trans)](../../../ligand/217173/info) | Agonist | <6ME9> |
| X-ray diffraction | Class A | [NK1R](http://www.uniprot.org/uniprot/P25103) | [Retinal (all-trans)](../../../ligand/217173/info) | Inverse agonist | <6J21> |
| X-ray diffraction | Class A | [NK1R](http://www.uniprot.org/uniprot/P25103) | [Retinal (11-cis)](../../../ligand/217172/info) | Antagonist | <6J20> |
| X-ray diffraction | Class A | [5HT2A](http://www.uniprot.org/uniprot/P28223) | [imaradenant](../../../ligand/1969/info) | Agonist | <6A93> |
| X-ray diffraction | Class A | [5HT2A](http://www.uniprot.org/uniprot/P28223) | [CHEMBL1615159](../../../ligand/54654/info) | PAM | <6A94> |
| Cryo-EM | Class A | [CNR1](http://www.uniprot.org/uniprot/P21554) | [2-[[(3R)-4-(4-Tert-butylphenyl)-3-[[2-(4-methoxyphenyl)sulfanyl-5-[methyl(propan-2-yl)sulfamoyl]benzoyl]amino]butanoyl]amino]acetic acid](../../../ligand/217235/info) | Agonist | <6N4B> |
| X-ray diffraction | Class A | [CNR2](http://www.uniprot.org/uniprot/P34972) | [CHEMBL1615159](../../../ligand/54654/info) | Agonist | <5ZTY> |
| X-ray diffraction | Class A | [NK1R](http://www.uniprot.org/uniprot/P25103) | [iperoxo](../../../ligand/2020/info) | Ago-PAM | <6HLL> |
| X-ray diffraction | Class A | [NK1R](http://www.uniprot.org/uniprot/P25103) | [LY2119620](../../../ligand/2356/info) | Agonist | <6HLO> |
| X-ray diffraction | Class A | [NK1R](http://www.uniprot.org/uniprot/P25103) | [iperoxo](../../../ligand/2020/info) | Antagonist | <6HLP> |
| X-ray diffraction | Class A | [ADRB1](http://www.uniprot.org/uniprot/P07700) | [ZM-241385](../../../ligand/4069/info) | Agonist | <6IBL> |
| X-ray diffraction | Class A | [CCR2](http://www.uniprot.org/uniprot/P41597) | [ramelteon](../../../ligand/3224/info) | Agonist | <6GPX> |
| X-ray diffraction | Class A | [CCR2](http://www.uniprot.org/uniprot/P41597) | [CHEMBL15060](../../../ligand/42959/info) | Agonist | <6GPS> |
| X-ray diffraction | Class A | [TA2R](http://www.uniprot.org/uniprot/P21731) | [2-iodo-melatonin](../../../ligand/70/info) | Agonist | <6IIU> |
| X-ray diffraction | Class A | [TA2R](http://www.uniprot.org/uniprot/P21731) | [agomelatine](../../../ligand/310/info) | Agonist | <6IIV> |
| X-ray diffraction | Class A | [NK1R](http://www.uniprot.org/uniprot/P25103) | [CHEMBL15060](../../../ligand/42959/info) | Agonist | [6.00E+59](6E59) |
| X-ray diffraction | Class A | [PE2R3](http://www.uniprot.org/uniprot/P43115) | [CHEMBL15060](../../../ligand/42959/info) | Agonist | <6AK3> |
| X-ray diffraction | Class A | [PE2R3](http://www.uniprot.org/uniprot/P43115) | [CHEMBL15060](../../../ligand/42959/info) | Agonist | <6M9T> |
| X-ray diffraction | Class A | [PE2R4](http://www.uniprot.org/uniprot/P35408) | [ramelteon](../../../ligand/3224/info) | Antagonist | <5YHL> |
| X-ray diffraction | Class A | [PE2R4](http://www.uniprot.org/uniprot/P35408) | [aprepitant](../../../ligand/444/info) | Antagonist | <5YWY> |
| X-ray diffraction | Class A | [ACM3](http://www.uniprot.org/uniprot/P08483) | [aprepitant](../../../ligand/444/info) | Antagonist | <5ZHP> |
| X-ray diffraction | Class A | [ACM2](http://www.uniprot.org/uniprot/P08172) | [risperidone](../../../ligand/3267/info) | Antagonist | <5ZK3> |
| X-ray diffraction | Class A | [ACM2](http://www.uniprot.org/uniprot/P08172) | [zotepine](../../../ligand/4074/info) | Agonist | <5ZKB> |
| X-ray diffraction | Class A | [ACM2](http://www.uniprot.org/uniprot/P08172) | [MDMB-Fubinaca](../../../ligand/2432/info) | Antagonist | <5YC8> |
| X-ray diffraction | Class A | [ACM2](http://www.uniprot.org/uniprot/P08172) | [AM10257](../../../ligand/371/info) | Antagonist | <5ZK8> |
| X-ray diffraction | Class A | [ACM2](http://www.uniprot.org/uniprot/P08172) | [CP 99994](../../../ligand/1162/info) | Antagonist | <5ZKC> |
| X-ray diffraction | Class A | [ADRB2](http://www.uniprot.org/uniprot/P07550) | [aprepitant](../../../ligand/444/info) | Antagonist | <6MXT> |
| X-ray diffraction | Class A | [CCR5](http://www.uniprot.org/uniprot/P51681) | [netupitant](../../../ligand/2714/info) | Agonist | <6AKX> |
| X-ray diffraction | Class A | [CCR5](http://www.uniprot.org/uniprot/P51681) | [arformoterol](../../../ligand/462/info) | Antagonist | <6AKY> |
| X-ray diffraction | Class A | [ADRB1](http://www.uniprot.org/uniprot/P07700) | [MK-0812](../../../ligand/217229/info) | Antagonist | <6H7L> |
| X-ray diffraction | Class A | [ADRB1](http://www.uniprot.org/uniprot/P07700) | [MK-0812](../../../ligand/217229/info) | Antagonist | <6H7N> |
| X-ray diffraction | Class A | [ADRB1](http://www.uniprot.org/uniprot/P07700) | [ramatroban](../../../ligand/3223/info) | Antagonist | <6H7J> |
| X-ray diffraction | Class A | [ADRB1](http://www.uniprot.org/uniprot/P07700) | [daltroban](../../../ligand/1280/info) | Antagonist | <6H7M> |
| X-ray diffraction | Class A | [ADRB1](http://www.uniprot.org/uniprot/P07700) | [L760735](../../../ligand/2211/info) | Agonist | <6H7O> |
| X-ray diffraction | Class A | [OPSD](http://www.uniprot.org/uniprot/P02699) | [PGE2](../../../ligand/3021/info) | Agonist | <6FUF> |
| X-ray diffraction | Class A | [PD2R2](http://www.uniprot.org/uniprot/Q9Y5Y4) | [Misoprostol-FA](../../../ligand/217226/info) | Antagonist | <6D26> |
| X-ray diffraction | Class A | [PD2R2](http://www.uniprot.org/uniprot/Q9Y5Y4) | [ONO-AE3-208-Br](../../../ligand/217224/info) | Antagonist | <6D27> |
| X-ray diffraction | Class A | [5HT2B](http://www.uniprot.org/uniprot/P41595) | [ONO-AE3-208](../../../ligand/217225/info) | Antagonist | <6DRY> |
| X-ray diffraction | Class A | [5HT2B](http://www.uniprot.org/uniprot/P41595) | [(1R,2R,4S,5S,7s)-7-({[4-fluoro-2-(thiophen-2-yl)phenyl]carbamoyl}oxy)-9,9-dimethyl-3-oxa-9-azatricyclo[3.3.1.0~2,4~]nonan-9-ium](../../../ligand/217223/info) | Antagonist | <6DS0> |
| X-ray diffraction | Class A | [5HT2B](http://www.uniprot.org/uniprot/P41595) | [CHEMBL558910](../../../ligand/194200/info) | Antagonist | <6DRX> |
| X-ray diffraction | Class A | [5HT2B](http://www.uniprot.org/uniprot/P41595) | [AF-DX 384](../../../ligand/217221/info) | Antagonist | <6DRZ> |
| Cryo-EM | Class A | [5HT1B](http://www.uniprot.org/uniprot/P28222) | [N-methyl scopolamine](../../../ligand/2783/info) | Antagonist | <6G79> |
| Cryo-EM | Class A | [AA1R](http://www.uniprot.org/uniprot/P30542) | [N-methyl scopolamine](../../../ligand/2783/info) | Antagonist | <6D9H> |
| X-ray diffraction | Class A | [PTAFR](http://www.uniprot.org/uniprot/P25105) | [N-methyl scopolamine](../../../ligand/2783/info) | Agonist | <5ZKP> |
| X-ray diffraction | Class A | [PTAFR](http://www.uniprot.org/uniprot/P25105) | [salmeterol](../../../ligand/3394/info) | Antagonist | <5ZKQ> |
| Cryo-EM | Class A | [AA2AR](http://www.uniprot.org/uniprot/P29274) | [CHEMBL4238063](../../../ligand/164835/info) | Antagonist | <6GDG> |
| X-ray diffraction | Class A | [FFAR1](http://www.uniprot.org/uniprot/O14842) | [CCR5 antagonist 34](../../../ligand/858/info) | Agonist | <5KW2> |
| X-ray diffraction | Class A | [NPY1R](http://www.uniprot.org/uniprot/P25929) | [Dobutamine](../../../ligand/217179/info) | Agonist | <5ZBQ> |
| X-ray diffraction | Class A | [NPY1R](http://www.uniprot.org/uniprot/P25929) | [(S)-Xamoterol](../../../ligand/216629/info) | Agonist | <5ZBH> |
| X-ray diffraction | Class A | [OPSD](http://www.uniprot.org/uniprot/P02699) | [LEVISOPRENALINE](../../../ligand/10123/info) | Agonist | <6FK7> |
| X-ray diffraction | Class A | [OPSD](http://www.uniprot.org/uniprot/P02699) | [levosalbutamol](../../../ligand/2275/info) | Agonist | <6FK9> |
| X-ray diffraction | Class A | [OPSD](http://www.uniprot.org/uniprot/P02699) | [(S)-Cyanopindolol](../../../ligand/217177/info) | Agonist | <6FKB> |
| X-ray diffraction | Class A | [OPSD](http://www.uniprot.org/uniprot/P02699) | [Retinal (all-trans)](../../../ligand/217173/info) | Antagonist | <6FKD> |
| X-ray diffraction | Class A | [OPSD](http://www.uniprot.org/uniprot/P02699) | [fevipiprant](../../../ligand/1595/info) | Antagonist | <6FK6> |
| X-ray diffraction | Class A | [OPSD](http://www.uniprot.org/uniprot/P02699) | [CHEMBL179036](../../../ligand/62849/info) | Agonist | <6FK8> |
| X-ray diffraction | Class A | [OPSD](http://www.uniprot.org/uniprot/P02699) | [methylergonovine](../../../ligand/2463/info) | Antagonist | <6FKA> |
| X-ray diffraction | Class A | [OPSD](http://www.uniprot.org/uniprot/P02699) | [LY266097](../../../ligand/217219/info) | Antagonist | <6FKC> |
| X-ray diffraction | Class A | [DRD2](http://www.uniprot.org/uniprot/P14416) | [lisuride](../../../ligand/2284/info) | Antagonist | <6CM4> |
| X-ray diffraction | Class A | [AA2AR](http://www.uniprot.org/uniprot/P29274) | [methysergide](../../../ligand/2468/info) | Agonist | <5WF6> |
| X-ray diffraction | Class A | [AA2AR](http://www.uniprot.org/uniprot/P29274) | [donitriptan](../../../ligand/1431/info) | Agonist | <5WF5> |
| X-ray diffraction | Class A | [5HT2C](http://www.uniprot.org/uniprot/P28335) | [adenosine](../../../ligand/280/info) | Antagonist | <6BQH> |
| X-ray diffraction | Class A | [5HT2C](http://www.uniprot.org/uniprot/P28335) | [foropafant](../../../ligand/1640/info) | Inverse agonist | <6BQG> |
| X-ray diffraction | Class A | [5HT1B](http://www.uniprot.org/uniprot/P28222) | [ABT-491](../../../ligand/229/info) | Agonist | <5V54> |
| X-ray diffraction | Class A | [AA2AR](http://www.uniprot.org/uniprot/P29274) | [NECA](../../../ligand/2707/info) | Agonist | <5OLH> |
| X-ray diffraction | Class A | [AA2AR](http://www.uniprot.org/uniprot/P29274) | [(3~{S})-3-cyclopropyl-3-[2-[1-[2-[2,2-dimethylpropyl-(6-methylpyridin-2-yl)carbamoyl]-5-methoxy-phenyl]piperidin-4-yl]-1-benzofuran-6-yl]propanoic acid](../../../ligand/217217/info) | Antagonist | <5OLV> |
| X-ray diffraction | Class A | [AA2AR](http://www.uniprot.org/uniprot/P29274) | [CHEMBL3747822](../../../ligand/136596/info) | Antagonist | <5OM1> |
| X-ray diffraction | Class A | [AA2AR](http://www.uniprot.org/uniprot/P29274) | [BMS-193885](../../../ligand/217216/info) | Agonist | <5OLG> |
| X-ray diffraction | Class A | [AA2AR](http://www.uniprot.org/uniprot/P29274) | [(2~{S})-2-(4-chlorophenyl)-3-methyl-1-spiro[1,3-benzodioxole-2,4'-piperidine]-1'-yl-butan-1-one](../../../ligand/217209/info) | Agonist | <5OLO> |
| X-ray diffraction | Class A | [AA2AR](http://www.uniprot.org/uniprot/P29274) | [(2~{S})-3-methyl-2-phenyl-1-spiro[1,3-benzodioxole-2,4'-piperidine]-1'-yl-butan-1-one](../../../ligand/217211/info) | Agonist | <5OLZ> |
| X-ray diffraction | Class A | [AA2AR](http://www.uniprot.org/uniprot/P29274) | [2-(4-Chlorophenyl)-1-spiro[1,3-benzodioxole-2,4'-piperidine]-1'-yl-ethanone](../../../ligand/217213/info) | Agonist | <5OM4> |
| X-ray diffraction | Class A | [OPRK](http://www.uniprot.org/uniprot/P41145) | [5-chloro-2-(2-oxo-2-spiro[1,3-benzodioxole-2,4'-piperidine]-1'-ylethyl)-3H-pyridin-6-one](../../../ligand/217215/info) | Agonist | <6B73> |
| X-ray diffraction | Class A | [AA2AR](http://www.uniprot.org/uniprot/P29274) | [COMPOUND SUMMARY](../../../ligand/217208/info) | Agonist | <6AQF> |
| X-ray diffraction | Class A | [C5AR1](http://www.uniprot.org/uniprot/P21730) | [(2~{R},3~{S})-3-azanyl-2-(4-chlorophenyl)-1-spiro[1,3-benzodioxole-2,4'-piperidine]-1'-yl-butan-1-one](../../../ligand/217210/info) | Agonist | <5O9H> |
| X-ray diffraction | Class A | [Q9WTK1](http://www.uniprot.org/uniprot/Q9WTK1) | [(2~{S})-2-(3,4-dichlorophenyl)-3-methyl-1-spiro[1,3-benzodioxole-2,4'-piperidine]-1'-yl-butan-1-one](../../../ligand/217212/info) | Agonist | <5X33> |
| X-ray diffraction | Class A | [AA2AR](http://www.uniprot.org/uniprot/P29274) | [3-[1'-[(2~{S})-2-(4-chlorophenyl)-3-methyl-butanoyl]spiro[1,3-benzodioxole-2,4'-piperidine]-5-yl]propanoic acid](../../../ligand/217214/info) | Inverse agonist | <5VRA> |
| X-ray diffraction | Class A | [OPSD](http://www.uniprot.org/uniprot/P02699) | [risperidone](../../../ligand/3267/info) | Agonist | <5WKT> |
| X-ray diffraction | Class A | [OX2R](http://www.uniprot.org/uniprot/O43614) | [UK-432,097](../../../ligand/3861/info) | Agonist | <5WS3> |
| X-ray diffraction | Class A | [OX2R](http://www.uniprot.org/uniprot/O43614) | [UK-432,097](../../../ligand/3861/info) | Inverse agonist | <5WQC> |
| X-ray diffraction | Class A | [DRD4](http://www.uniprot.org/uniprot/P21917) | [ritanserin](../../../ligand/3268/info) | Agonist | <5WIU> |
| X-ray diffraction | Class A | [DRD4](http://www.uniprot.org/uniprot/P21917) | [ergotamine](../../../ligand/1545/info) | Antagonist | <5WIV> |
| X-ray diffraction | Class A | [AA2AR](http://www.uniprot.org/uniprot/P29274) | [CHEMBL428892](../../../ligand/166481/info) | Antagonist | <5NM2> |
| X-ray diffraction | Class A | [AA2AR](http://www.uniprot.org/uniprot/P29274) | [vipadenant](../../../ligand/3921/info) | Antagonist | <5NLX> |
| X-ray diffraction | Class A | [AA2AR](http://www.uniprot.org/uniprot/P29274) | [CHEMBL1671936](../../../ligand/57543/info) | Antagonist | <5NM4> |
| X-ray diffraction | Class A | [ADRB2](http://www.uniprot.org/uniprot/P07550) | [CHEMBL2024114](../../../ligand/73924/info) | Antagonist | <5X7D> |
| X-ray diffraction | Class A | [EDNRB](http://www.uniprot.org/uniprot/P24530) | [ZM-241385](../../../ligand/4069/info) | Antagonist | <5X93> |
| X-ray diffraction | Class A | [EDNRB](http://www.uniprot.org/uniprot/P24530) | [tozadenant](../../../ligand/3774/info) | Antagonist | <5XPR> |
| X-ray diffraction | Class A | [5HT2B](http://www.uniprot.org/uniprot/P41595) | [CHEMBL2024114](../../../ligand/73924/info) | Antagonist | <5TUD> |
| X-ray diffraction | Class A | [AA1R](http://www.uniprot.org/uniprot/P30542) | [CHEMBL2024114](../../../ligand/73924/info) | Agonist | <5N2S> |
| X-ray diffraction | Class A | [AA2AR](http://www.uniprot.org/uniprot/P29274) | [MP1104](../../../ligand/217206/info) | Antagonist | <5MZJ> |
| X-ray diffraction | Class A | [AA2AR](http://www.uniprot.org/uniprot/P29274) | [ZM-241385](../../../ligand/4069/info) | Antagonist | <5N2R> |
| X-ray diffraction | Class A | [AA2AR](http://www.uniprot.org/uniprot/P29274) | [NDT9513727](../../../ligand/2703/info) | Antagonist | <5MZP> |
| X-ray diffraction | Class A | [CNR1](http://www.uniprot.org/uniprot/P21554) | [BIIL 260](../../../ligand/627/info) | Antagonist | <5XR8> |
| X-ray diffraction | Class A | [CNR1](http://www.uniprot.org/uniprot/P21554) | [ZM-241385](../../../ligand/4069/info) | unknown | <5XRA> |
| X-ray diffraction | Class A | [FFAR1](http://www.uniprot.org/uniprot/O14842) | [B-Octylglucoside](../../../ligand/217185/info) | Antagonist | <5TZY> |
| X-ray diffraction | Class A | [FFAR1](http://www.uniprot.org/uniprot/O14842) | [EMPA](../../../ligand/1519/info) | Antagonist | <5TZR> |
| X-ray diffraction | Class A | [AA2AR](http://www.uniprot.org/uniprot/P29274) | [EMPA](../../../ligand/1519/info) | Antagonist | <5JTB> |
| X-ray diffraction | Class A | [AA2AR](http://www.uniprot.org/uniprot/P29274) | [[3H]NEMONAPRIDE](../../../ligand/84883/info) | Antagonist | <5UVI> |
| X-ray diffraction | Class A | [PAR2](http://www.uniprot.org/uniprot/P55085) | [[3H]NEMONAPRIDE](../../../ligand/84883/info) | Antagonist | <5NDD> |
| X-ray diffraction | Class A | [PAR2](http://www.uniprot.org/uniprot/P55085) | [ZM-241385](../../../ligand/4069/info) | Antagonist | <5NDZ> |
| X-ray diffraction | Class A | [AGTR2](http://www.uniprot.org/uniprot/P50052) | [ZM-241385](../../../ligand/4069/info) | Antagonist | <5UNF> |
| X-ray diffraction | Class A | [AGTR2](http://www.uniprot.org/uniprot/P50052) | [ZM-241385](../../../ligand/4069/info) | Antagonist | <5UNG> |
| X-ray diffraction | Class A | [AGTR2](http://www.uniprot.org/uniprot/P50052) | [4-carbamoyl-N-[(2R)-2-cyclohexyl-2-phenylacetyl]-L-phenylalanyl-3-bromo-N-methyl-L-phenylalaninamide](../../../ligand/217205/info) | Antagonist | <5UNH> |
| X-ray diffraction | Class A | [OPSD](http://www.uniprot.org/uniprot/P02699) | [Carazolol](../../../ligand/217176/info) | Antagonist | <5TE3> |
| X-ray diffraction | Class A | [OPSD](http://www.uniprot.org/uniprot/P02699) | [K-8794](../../../ligand/2134/info) | Antagonist | <5TE5> |
| X-ray diffraction | Class A | [AA1R](http://www.uniprot.org/uniprot/P30542) | [bosentan](../../../ligand/697/info) | Agonist | <5UEN> |
| X-ray diffraction | Class A | [AA2AR](http://www.uniprot.org/uniprot/P29274) | [ergotamine](../../../ligand/1545/info) | Agonist | <5UIG> |
| X-ray diffraction | Class A | [5HT2B](http://www.uniprot.org/uniprot/P41595) | [PSB36](../../../ligand/217204/info) | Antagonist | <5TVN> |
| X-ray diffraction | Class A | [CCR2](http://www.uniprot.org/uniprot/P41597) | [theophylline](../../../ligand/3728/info) | Antagonist | <5T1A> |
| X-ray diffraction | Class A | [CCR9](http://www.uniprot.org/uniprot/P51686) | [PSB36](../../../ligand/217204/info) | Antagonist | <5LWE> |
| X-ray diffraction | Class A | [CNR1](http://www.uniprot.org/uniprot/P21554) | [caffeine](../../../ligand/765/info) | Agonist | <5U09> |
| X-ray diffraction | Class A | [CNR1](http://www.uniprot.org/uniprot/P21554) | [AM841](../../../ligand/385/info) | Agonist | <5TGZ> |
| X-ray diffraction | Class A | [AA2AR](http://www.uniprot.org/uniprot/P29274) | [AM11542](../../../ligand/372/info) | Agonist | <5K2B> |
| X-ray diffraction | Class A | [AA2AR](http://www.uniprot.org/uniprot/P29274) | [MK-8666](../../../ligand/166871/info) | PAM | <5K2D> |
| X-ray diffraction | Class A | [AA2AR](http://www.uniprot.org/uniprot/P29274) | [(2S,3R)-3-Cyclopropyl-3-[(2R)-2-[1-[(1S)-1-[5-fluoro-2-(trifluoromethoxy)phenyl]ethyl]piperidin-4-yl]-3,4-dihydro-2H-chromen-7-yl]-2-methylpropanoic acid](../../../ligand/217201/info) | Agonist | <5K2A> |
| X-ray diffraction | Class A | [AA2AR](http://www.uniprot.org/uniprot/P29274) | [MK-8666](../../../ligand/166871/info) | Antagonist | <5K2C> |
| X-ray diffraction | Class A | [ADRB2](http://www.uniprot.org/uniprot/P07550) | [ZM-241385](../../../ligand/4069/info) | Antagonist | <5D6L> |
| X-ray diffraction | Class A | [OPSD](http://www.uniprot.org/uniprot/P02699) | [ZM-241385](../../../ligand/4069/info) | Antagonist | <5EN0> |
| X-ray diffraction | Class A | [OPSD](http://www.uniprot.org/uniprot/P02699) | [AZ8838](../../../ligand/535/info) | Antagonist | <5DYS> |
| X-ray diffraction | Class A | [AA2AR](http://www.uniprot.org/uniprot/P29274) | [AZ3451](../../../ligand/533/info) | Antagonist | <5G53> |
| X-ray diffraction | Class A | [ADRB2](http://www.uniprot.org/uniprot/P07550) | [compound 1 [PMID: 28379944]](../../../ligand/1002/info) | Antagonist | <5JQH> |
| X-ray diffraction | Class A | [AA2AR](http://www.uniprot.org/uniprot/P29274) | [compound 1 [PMID: 28379944]](../../../ligand/1002/info) | Antagonist | <5IU4> |
| X-ray diffraction | Class A | [AA2AR](http://www.uniprot.org/uniprot/P29274) | [compound 2 [PMID: 28379944]](../../../ligand/1045/info) | unknown | <5IU8> |
| X-ray diffraction | Class A | [AA2AR](http://www.uniprot.org/uniprot/P29274) | [B-Octylglucoside](../../../ligand/217185/info) | Agonist | <5IUB> |
| X-ray diffraction | Class A | [AA2AR](http://www.uniprot.org/uniprot/P29274) | [10,20-Methanoretinal](../../../ligand/217197/info) | Antagonist | <5IU7> |
| X-ray diffraction | Class A | [AA2AR](http://www.uniprot.org/uniprot/P29274) | [DU172](../../../ligand/1474/info) | Antagonist | <5IUA> |
| X-ray diffraction | Class A | [ACM4](http://www.uniprot.org/uniprot/P08173) | [5-Amino-N-[(2-Methoxyphenyl)methyl]-2-(3-Methylphenyl)-2h-1,2,3-Triazole-4-Carboximidamide](../../../ligand/217196/info) | Agonist | <5DSG> |
| X-ray diffraction | Class A | [ACM1](http://www.uniprot.org/uniprot/P11229) | [Lysergide](../../../ligand/2386/info) | Antagonist | <5CXV> |
| X-ray diffraction | Class A | [OX1R](http://www.uniprot.org/uniprot/O43613) | [CCR2-RA-[R]](../../../ligand/857/info) | Antagonist | <4ZJ8> |
| X-ray diffraction | Class A | [OX1R](http://www.uniprot.org/uniprot/O43613) | [BMS-681](../../../ligand/664/info) | Antagonist | <4ZJC> |
| X-ray diffraction | Class A | [ADRB2](http://www.uniprot.org/uniprot/P07550) | [vercirnon](../../../ligand/3908/info) | Inverse agonist | <5D5A> |
| X-ray diffraction | Class A | [ADRB2](http://www.uniprot.org/uniprot/P07550) | [taranabant](../../../ligand/3686/info) | Antagonist | <5D5B> |
| X-ray diffraction | Class A | [ADRB1](http://www.uniprot.org/uniprot/P07700) | [AM6538](../../../ligand/382/info) | Antagonist | <5F8U> |
| X-ray diffraction | Class A | [OPSD](http://www.uniprot.org/uniprot/P02699) | [ZM-241385](../../../ligand/4069/info) | Antagonist | <4X1H> |
| X-ray diffraction | Class A | [OPRX](http://www.uniprot.org/uniprot/P41146) | [ZM-241385](../../../ligand/4069/info) | Antagonist | <5DHG> |
| X-ray diffraction | Class A | [OPRX](http://www.uniprot.org/uniprot/P41146) | [ZM-241385](../../../ligand/4069/info) | Antagonist | <5DHH> |
| X-ray diffraction | Class A | [AGTR1](http://www.uniprot.org/uniprot/P30556) | [ZM-241385](../../../ligand/4069/info) | Inverse agonist | <4ZUD> |
| X-ray diffraction | Class A | [ADRB1](http://www.uniprot.org/uniprot/P07700) | [Carazolol](../../../ligand/217176/info) | Agonist | <5A8E> |
| X-ray diffraction | Class A | [OPRM](http://www.uniprot.org/uniprot/P42866) | [Retinal (all-trans)](../../../ligand/217173/info) | Agonist | <5C1M> |
| X-ray diffraction | Class A | [OPSD](http://www.uniprot.org/uniprot/P31356) | [Retinal (all-trans)](../../../ligand/217173/info) | Agonist | <4WW3> |
| X-ray diffraction | Class A | [LPAR1](http://www.uniprot.org/uniprot/Q92633) | [NECA](../../../ligand/2707/info) | Inverse agonist | <4Z34> |
| X-ray diffraction | Class A | [LPAR1](http://www.uniprot.org/uniprot/Q92633) | [Carazolol](../../../ligand/217176/info) | Antagonist | <4Z36> |
| X-ray diffraction | Class A | [LPAR1](http://www.uniprot.org/uniprot/Q92633) | [ZM-241385](../../../ligand/4069/info) | Antagonist | <4Z35> |
| X-ray diffraction | Class A | [AGTR1](http://www.uniprot.org/uniprot/P30556) | [CHEMBL3934661](../../../ligand/147500/info) | Antagonist | <4YAY> |
| X-ray diffraction | Class A | [AA2AR](http://www.uniprot.org/uniprot/P29274) | [CHEMBL184061](../../../ligand/65783/info) | Antagonist | <4UHR> |
| X-ray diffraction | Class A | [AA2AR](http://www.uniprot.org/uniprot/P29274) | [2-(Furan-2-yl)-5-N-[2-(4-phenylpiperidin-1-yl)ethyl]-1H-[1,2,4]triazolo[1,5-a][1,3,5]triazin-8-ium-5,7-diamine](../../../ligand/217194/info) | Antagonist | <4UG2> |
| X-ray diffraction | Class A | [P2RY1](http://www.uniprot.org/uniprot/P47900) | [2-(Furan-2-yl)-5-N-[3-(4-phenylpiperazin-1-yl)propyl]-1H-[1,2,4]triazolo[1,5-a][1,3,5]triazin-8-ium-5,7-diamine](../../../ligand/217195/info) | Antagonist | <4XNV> |
| X-ray diffraction | Class A | [P2RY1](http://www.uniprot.org/uniprot/P47900) | [tiotropium](../../../ligand/3752/info) | Antagonist | <4XNW> |
| X-ray diffraction | Class A | [OX2R](http://www.uniprot.org/uniprot/O43614) | [0HK](../../../ligand/217192/info) | Antagonist | <4S0V> |
| X-ray diffraction | Class A | [ACM3](http://www.uniprot.org/uniprot/P08483) | [suvorexant](../../../ligand/3644/info) | Antagonist | <4U14> |
| X-ray diffraction | Class A | [ACM3](http://www.uniprot.org/uniprot/P08483) | [SB-674042](../../../ligand/3464/info) | Inverse agonist | <4U16> |
| X-ray diffraction | Class A | [ACM3](http://www.uniprot.org/uniprot/P08483) | [Carazolol](../../../ligand/217176/info) | Inverse agonist | <4U15> |
| X-ray diffraction | Class A | [OPSD](http://www.uniprot.org/uniprot/P02699) | [Carazolol](../../../ligand/217176/info) | Antagonist | <4PXF> |
| X-ray diffraction | Class A | [ADRB2](http://www.uniprot.org/uniprot/P07550) | [(S)-Cyanopindolol](../../../ligand/217177/info) | unknown | <4QKX> |
| X-ray diffraction | Class A | [FFAR1](http://www.uniprot.org/uniprot/O14842) | [B-Nonylglucoside](../../../ligand/217191/info) | Antagonist | <4PHU> |
| X-ray diffraction | Class A | [P2Y12](http://www.uniprot.org/uniprot/Q9H244) | [CHEMBL1783826](../../../ligand/62307/info) | Antagonist | <4PY0> |
| X-ray diffraction | Class A | [P2Y12](http://www.uniprot.org/uniprot/Q9H244) | [SB 612111](../../../ligand/3460/info) | Inverse agonist | <4PXZ> |
| X-ray diffraction | Class A | [ADRB1](http://www.uniprot.org/uniprot/P07700) | [OLMESARTAN](../../../ligand/43900/info) | Inverse agonist | <4BVN> |
| X-ray diffraction | Class A | [P2Y12](http://www.uniprot.org/uniprot/Q9H244) | [7-methylcyanopindolol](../../../ligand/217190/info) | Agonist | <4NTJ> |
| X-ray diffraction | Class A | [OPRD](http://www.uniprot.org/uniprot/P41143) | [BU72](../../../ligand/724/info) | Agonist | <4N6H> |
| X-ray diffraction | Class A | [5HT2B](http://www.uniprot.org/uniprot/P41595) | [Retinal (all-trans)](../../../ligand/217173/info) | Antagonist | <4NC3> |
| X-ray diffraction | Class A | [ACM2](http://www.uniprot.org/uniprot/P08172) | [ONO-9780307](../../../ligand/2884/info) | Antagonist | <4MQS> |
| X-ray diffraction | Class A | [ACM2](http://www.uniprot.org/uniprot/P08172) | [ONO-3080573](../../../ligand/2876/info) | Antagonist | <4MQT> |
| X-ray diffraction | Class A | [OPSD](http://www.uniprot.org/uniprot/P02699) | [ONO-9910539](../../../ligand/2885/info) | Antagonist | <4J4Q> |
| X-ray diffraction | Class A | [ADRB2](http://www.uniprot.org/uniprot/P07550) | [ZD-7155](../../../ligand/4061/info) | Agonist | <4LDL> |
| X-ray diffraction | Class A | [ADRB2](http://www.uniprot.org/uniprot/P07550) | [CGS 21680](../../../ligand/889/info) | Agonist | <4LDE> |
| X-ray diffraction | Class A | [ADRB2](http://www.uniprot.org/uniprot/P07550) | [CGS 21680](../../../ligand/889/info) | NAM | <4LDO> |
| X-ray diffraction | Class A | [CCR5](http://www.uniprot.org/uniprot/P51681) | [BMS compound 16 [PMID:23368907]](../../../ligand/682/info) | Antagonist | <4MBS> |
| X-ray diffraction | Class A | [ADRB1](http://www.uniprot.org/uniprot/P07700) | [MRS2500](../../../ligand/2602/info) | Antagonist | <3ZPR> |
| X-ray diffraction | Class A | [ADRB1](http://www.uniprot.org/uniprot/P07700) | [suvorexant](../../../ligand/3644/info) | Antagonist | <3ZPQ> |
| X-ray diffraction | Class A | [5HT1B](http://www.uniprot.org/uniprot/P28222) | [tiotropium](../../../ligand/3752/info) | Antagonist | <4IAQ> |
| X-ray diffraction | Class A | [5HT1B](http://www.uniprot.org/uniprot/P28222) | [N-methyl scopolamine](../../../ligand/2783/info) | Antagonist | <4IAR> |
| X-ray diffraction | Class A | [5HT2B](http://www.uniprot.org/uniprot/P41595) | [tiotropium](../../../ligand/3752/info) | unknown | <4IB4> |
| X-ray diffraction | Class A | [PAR1](http://www.uniprot.org/uniprot/P25116) | [B-Octylglucoside](../../../ligand/217185/info) | Agonist | <3VW7> |
| X-ray diffraction | Class A | [ADRB2](http://www.uniprot.org/uniprot/P07550) | [Q27453560](../../../ligand/217187/info) | Agonist | <4GBR> |
| X-ray diffraction | Class A | [AA2AR](http://www.uniprot.org/uniprot/P29274) | [fasiglifam](../../../ligand/1587/info) | Partial agonist | <4EIY> |
| X-ray diffraction | Class A | [ADRB1](http://www.uniprot.org/uniprot/P07700) | [2MeSATP](../../../ligand/77/info) | Agonist | <4AMJ> |
| X-ray diffraction | Class A | [ADRB1](http://www.uniprot.org/uniprot/P07700) | [2MeSADP](../../../ligand/75/info) | Antagonist | <4AMI> |
| X-ray diffraction | Class A | [OPRD](http://www.uniprot.org/uniprot/P32300) | [(S)-Cyanopindolol](../../../ligand/217177/info) | Antagonist | <4EJ4> |
| X-ray diffraction | Class A | [OPRX](http://www.uniprot.org/uniprot/P41146) | [AZD1283](../../../ligand/536/info) | Antagonist | <4EA3> |
| X-ray diffraction | Class A | [AA2AR](http://www.uniprot.org/uniprot/P29274) | [naltrindole](../../../ligand/2678/info) | Agonist | <3UZA> |
| X-ray diffraction | Class A | [AA2AR](http://www.uniprot.org/uniprot/P29274) | [ergotamine](../../../ligand/1545/info) | Agonist | <3UZC> |
| X-ray diffraction | Class A | [OPRK](http://www.uniprot.org/uniprot/P41145) | [iperoxo](../../../ligand/2020/info) | Agonist | <4DJH> |
| X-ray diffraction | Class A | [OPRM](http://www.uniprot.org/uniprot/P42866) | [iperoxo](../../../ligand/2020/info) | PAM | <4DKL> |
| X-ray diffraction | Class A | [ACM3](http://www.uniprot.org/uniprot/P08483) | [LY2119620](../../../ligand/2356/info) | unknown | <4DAJ> |
| X-ray diffraction | Class A | [S1PR1](http://www.uniprot.org/uniprot/P21453) | [B-Octylglucoside](../../../ligand/217185/info) | Agonist | <3V2Y> |
| X-ray diffraction | Class A | [S1PR1](http://www.uniprot.org/uniprot/P21453) | [hydroxybenzylisoproterenol](../../../ligand/216417/info) | Agonist | <3V2W> |
| X-ray diffraction | Class A | [AA2AR](http://www.uniprot.org/uniprot/P29274) | [CHEMBL1615159](../../../ligand/54654/info) | Agonist | <3VGA> |
| X-ray diffraction | Class A | [AA2AR](http://www.uniprot.org/uniprot/P29274) | [(-)-adrenaline](../../../ligand/287/info) | Antagonist | <3VG9> |
| X-ray diffraction | Class A | [ACM2](http://www.uniprot.org/uniprot/P08172) | [maraviroc](../../../ligand/2416/info) | Antagonist | <3UON> |
| X-ray diffraction | Class A | [OPSD](http://www.uniprot.org/uniprot/P02699) | [CHEMBL1559535](../../../ligand/48601/info) | Antagonist | <4A4M> |
| X-ray diffraction | Class A | [AA2AR](http://www.uniprot.org/uniprot/P29274) | [CHEMBL200234](../../../ligand/72490/info) | Agonist | <3PWH> |
| X-ray diffraction | Class A | [AA2AR](http://www.uniprot.org/uniprot/P29274) | [dihydroergotamine](../../../ligand/1387/info) | Agonist | <3RFM> |
| X-ray diffraction | Class A | [AA2AR](http://www.uniprot.org/uniprot/P29274) | [ergotamine](../../../ligand/1545/info) | Agonist | <3REY> |
| X-ray diffraction | Class A | [OPSD](http://www.uniprot.org/uniprot/P31356) | [ergotamine](../../../ligand/1545/info) | Antagonist | <3AYM> |
| X-ray diffraction | Class A | [OPSD](http://www.uniprot.org/uniprot/P31356) | [vorapaxar](../../../ligand/3932/info) | Inverse agonist | <3AYN> |
| X-ray diffraction | Class A | [ADRB2](http://www.uniprot.org/uniprot/P07550) | [Carazolol](../../../ligand/217176/info) | Antagonist | <3SN6> |
| X-ray diffraction | Class A | [HRH1](http://www.uniprot.org/uniprot/P35367) | [ZM-241385](../../../ligand/4069/info) | Inverse agonist | <3RZE> |
| X-ray diffraction | Class A | [ADRB1](http://www.uniprot.org/uniprot/P07700) | [CHEMBL3799125](../../../ligand/139303/info) | Agonist | <2YCY> |
| X-ray diffraction | Class A | [ADRB1](http://www.uniprot.org/uniprot/P07700) | [Bucindolol](../../../ligand/217184/info) | Antagonist | <2YCX> |
| X-ray diffraction | Class A | [ADRB1](http://www.uniprot.org/uniprot/P07700) | [naltrindole](../../../ligand/2678/info) | Antagonist | <2YCW> |
| X-ray diffraction | Class A | [ADRB1](http://www.uniprot.org/uniprot/P07700) | [compound 24 [PMID: 16451050]](../../../ligand/1024/info) | Antagonist | <2YCZ> |
| X-ray diffraction | Class A | [AA2AR](http://www.uniprot.org/uniprot/P29274) | [compound 4g [PMID: 22220592]](../../../ligand/1086/info) | Antagonist | <2YDV> |
| X-ray diffraction | Class A | [AA2AR](http://www.uniprot.org/uniprot/P29274) | [CHEMBL2024114](../../../ligand/73924/info) | Antagonist | <2YDO> |
| X-ray diffraction | Class A | [ADRB1](http://www.uniprot.org/uniprot/P07700) | [JDTic](../../../ligand/2074/info) | Antagonist | <2Y01> |
| X-ray diffraction | Class A | [OPSD](http://www.uniprot.org/uniprot/P02699) | [Methyl 4-[[(4R,4aS,7R,7aR,12bS)-3-(cyclopropylmethyl)-4a,9-dihydroxy-1,2,4,5,6,7,7a,13-octahydro-4,12-methanobenzofuro[3,2-e]isoquinolin-7-yl]amino]-4-oxobutanoate](../../../ligand/217183/info) | Antagonist | <2X72> |
| X-ray diffraction | Class A | [AA2AR](http://www.uniprot.org/uniprot/P29274) | [tiotropium](../../../ligand/3752/info) | Antagonist | <3QAK> |
| X-ray diffraction | Class A | [OPSD](http://www.uniprot.org/uniprot/P02699) | [W146](../../../ligand/3992/info) | Antagonist | <3PQR> |
| X-ray diffraction | Class A | [OPSD](http://www.uniprot.org/uniprot/P02699) | [W146](../../../ligand/3992/info) | Antagonist | <3PXO> |
| X-ray diffraction | Class A | [ADRB2](http://www.uniprot.org/uniprot/P07550) | [ZM-241385](../../../ligand/4069/info) | Antagonist | <3P0G> |
| X-ray diffraction | Class A | [OPSD](http://www.uniprot.org/uniprot/P02699) | [ZM-241385](../../../ligand/4069/info) | Antagonist | <3OAX> |
| X-ray diffraction | Class A | [ADRB1](http://www.uniprot.org/uniprot/P07700) | [CHEMBL558910](../../../ligand/194200/info) | Agonist | <2Y00> |
| X-ray diffraction | Class A | [ADRB1](http://www.uniprot.org/uniprot/P07700) | [Retinal (all-trans)](../../../ligand/217173/info) | Antagonist | <2Y02> |
| X-ray diffraction | Class A | [ADRB1](http://www.uniprot.org/uniprot/P07700) | [ZM-241385](../../../ligand/4069/info) | Antagonist | <2Y04> |
| X-ray diffraction | Class A | [ADRB1](http://www.uniprot.org/uniprot/P07700) | [caffeine](../../../ligand/765/info) | Antagonist | <2Y03> |
| X-ray diffraction | Class A | [ADRB2](http://www.uniprot.org/uniprot/P07550) | [xanthine amine congener](../../../ligand/4023/info) | Agonist | <3PDS> |
| X-ray diffraction | Class A | [DRD3](http://www.uniprot.org/uniprot/P35462) | [Retinal (all-trans)](../../../ligand/217173/info) | Inverse agonist | <3PBL> |
| X-ray diffraction | Class A | [CXCR4](http://www.uniprot.org/uniprot/P61073) | [Retinal (9-cis)](../../../ligand/217175/info) | Agonist | <3OE8> |
| X-ray diffraction | Class A | [CXCR4](http://www.uniprot.org/uniprot/P61073) | [CHEMBL1615159](../../../ligand/54654/info) | Antagonist | <3ODU> |
| X-ray diffraction | Class A | [CXCR4](http://www.uniprot.org/uniprot/P61073) | [doxepin](../../../ligand/1438/info) | Antagonist | <3OE6> |
| X-ray diffraction | Class A | [CXCR4](http://www.uniprot.org/uniprot/P61073) | [(S)-Cyanopindolol](../../../ligand/217177/info) | Antagonist | <3OE9> |
| X-ray diffraction | Class A | [ADRB2](http://www.uniprot.org/uniprot/P07550) | [(S)-Cyanopindolol](../../../ligand/217177/info) | Antagonist | <3NY8> |
| X-ray diffraction | Class A | [ADRB2](http://www.uniprot.org/uniprot/P07550) | [Carazolol](../../../ligand/217176/info) | Antagonist | <3NYA> |
| X-ray diffraction | Class A | [ADRB2](http://www.uniprot.org/uniprot/P07550) | [Iodocyanopindolol](../../../ligand/217182/info) | Agonist | <3NY9> |
| X-ray diffraction | Class A | [AA2AR](http://www.uniprot.org/uniprot/P29274) | [NECA](../../../ligand/2707/info) | Agonist | <3EML> |
| X-ray diffraction | Class A | [OPSD](http://www.uniprot.org/uniprot/P02699) | [adenosine](../../../ligand/280/info) | Partial agonist | <3C9M> |
| X-ray diffraction | Class A | [OPSD](http://www.uniprot.org/uniprot/P02699) | [Dobutamine](../../../ligand/217181/info) | Agonist | <3C9L> |
| X-ray diffraction | Class A | [ADRB1](http://www.uniprot.org/uniprot/P07700) | [Retinal (all-trans)](../../../ligand/217173/info) | Agonist | <2VT4> |
| X-ray diffraction | Class A | [ADRB2](http://www.uniprot.org/uniprot/P07550) | [UK-432,097](../../../ligand/3861/info) | Agonist | <3D4S> |
| X-ray diffraction | Class A | [OPSD](http://www.uniprot.org/uniprot/P31356) | [Retinal (all-trans)](../../../ligand/217173/info) | Agonist | <2Z73> |
| X-ray diffraction | Class A | [OPSD](http://www.uniprot.org/uniprot/P31356) | [Retinal (all-trans)](../../../ligand/217173/info) | Agonist | <2ZIY> |
| X-ray diffraction | Class A | [ADRB2](http://www.uniprot.org/uniprot/P07550) | [Carazolol](../../../ligand/217176/info) | Inverse agonist | <2RH1> |
| X-ray diffraction | Class A | [OPSD](http://www.uniprot.org/uniprot/P02699) | [Retinal (9-cis)](../../../ligand/217175/info) | Inverse agonist | <2PED> |
| X-ray diffraction | Class A | [OPSD](http://www.uniprot.org/uniprot/P02699) | [Retinal (11-cis)](../../../ligand/217172/info) | Inverse agonist | <2J4Y> |
| X-ray diffraction | Class A | [OPSD](http://www.uniprot.org/uniprot/P02699) | [Retinal (11-cis)](../../../ligand/217172/info) | Inverse agonist | <2I35> |
| X-ray diffraction | Class A | [OPSD](http://www.uniprot.org/uniprot/P02699) | [Retinal (all-trans)](../../../ligand/217173/info) | Agonist | <2G87> |
| X-ray diffraction | Class A | [OPSD](http://www.uniprot.org/uniprot/P02699) | [Retinal (all-trans)](../../../ligand/217173/info) | Agonist | <2HPY> |
| X-ray diffraction | Class A | [OPSD](http://www.uniprot.org/uniprot/P02699) | [Retinal (11-cis)](../../../ligand/217172/info) | Inverse agonist | <1U19> |
| X-ray diffraction | Class A | [OPSD](http://www.uniprot.org/uniprot/P02699) | [Retinal (11-cis)](../../../ligand/217172/info) | Inverse agonist | <1GZM> |
| X-ray diffraction | Class A | [OPSD](http://www.uniprot.org/uniprot/P02699) | [Retinal (11-cis)](../../../ligand/217172/info) | Inverse agonist | <1L9H> |
| X-ray diffraction | Class A | [OPSD](http://www.uniprot.org/uniprot/P02699) | [Retinal (11-cis)](../../../ligand/217172/info) | Inverse agonist | <1HZX> |
| X-ray diffraction | Class A | [OPSD](http://www.uniprot.org/uniprot/P02699) | [Retinal (11-cis)](../../../ligand/217172/info) | Inverse agonist | <1F88> |

Supplementary Table 2. 22 Solved class B GPCR structures complexed with synthetic orthosteric modulators

| Structure Type | GPCR Type | GPCR | Modulator | Modulator Type | PDB code |
| --- | --- | --- | --- | --- | --- |
| Cryo-EM | Class B1 | [PTH1R](http://www.uniprot.org/uniprot/Q03431) | [abaloparatide](../../../ligand/216018/info) | Agonist | <8FLS> |
| Cryo-EM | Class B1 | [VIPR1](http://www.uniprot.org/uniprot/P32241) | [Vasoactive intestinal polypeptide](../../../ligand/214560/info) | Agonist | <8E3Z> |
| Cryo-EM | Class B1 | [GLP1R](http://www.uniprot.org/uniprot/P43220) | [CHEMBL2158411](../../../ligand/80779/info) | Agonist | <7X8R> |
| Cryo-EM | Class B1 | [GLP1R](http://www.uniprot.org/uniprot/P43220) | [CHEMBL2158488](../../../ligand/80791/info) | Agonist | <7X8S> |
| Cryo-EM | Class B1 | [GLP1R](http://www.uniprot.org/uniprot/P43220) | [CHEMBL5191519](../../../ligand/190723/info) | Agonist | <7S15> |
| Cryo-EM | Class B1 | [GIPR](http://www.uniprot.org/uniprot/P48546) | [GIP](../../../ligand/216836/info) | Agonist | <7DTY> |
| Cryo-EM | Class B1 | [GLP1R](http://www.uniprot.org/uniprot/P43220) | [danuglipron](../../../ligand/1285/info) | Agonist | <7LCI> |
| Cryo-EM | Class B1 | [GLP1R](http://www.uniprot.org/uniprot/P43220) | [danuglipron](../../../ligand/1285/info) | Agonist | <7LCJ> |
| Cryo-EM | Class B1 | [GLP1R](http://www.uniprot.org/uniprot/P43220) | [danuglipron](../../../ligand/1285/info) | Agonist | <7LCK> |
| Cryo-EM | Class B1 | [GLP1R](http://www.uniprot.org/uniprot/P43220) | [oforglipron](../../../ligand/2854/info) | Agonist | <6XOX> |
| Cryo-EM | Class B1 | [GLP1R](http://www.uniprot.org/uniprot/P43220) | [danuglipron](../../../ligand/1285/info) | Agonist | <6X1A> |
| Cryo-EM | Class B1 | [GLP1R](http://www.uniprot.org/uniprot/P43220) | [CHU-128](../../../ligand/217265/info) | Agonist | <6X19> |
| Cryo-EM | Class B1 | [GLP1R](http://www.uniprot.org/uniprot/P43220) | [2-[[4-[6-[(4-cyano-2-fluoranyl-phenyl)methoxy]pyridin-2-yl]-3,6-dihydro-2~{H}-pyridin-1-yl]methyl]-3-[[(2~{S})-oxetan-2-yl]methyl]imidazo[4,5-b]pyridine-5-carboxylic acid](../../../ligand/217262/info) | Agonist | <7C2E> |
| Cryo-EM | Class B1 | [GLP1R](http://www.uniprot.org/uniprot/P43220) | [CHEMBL3950796](../../../ligand/149532/info) | Agonist | <6ORV> |
| X-ray diffraction | Class B1 | [CRFR1](http://www.uniprot.org/uniprot/P34998-2) | [CP 376,395](../../../ligand/1153/info) | Antagonist | <4Z9G> |
| X-ray diffraction | Class B1 | [CRFR1](http://www.uniprot.org/uniprot/P34998-2) | [CP 376,395](../../../ligand/1153/info) | Antagonist | <4K5Y> |
| Cryo-EM | Class B2 | [AGRF1](http://www.uniprot.org/uniprot/Q5T601) | [[1-Myristoyl-glycerol-3-YL]phosphonylcholine](../../../ligand/217398/info) | Agonist | <8G2Y> |
| Cryo-EM | Class B2 | [AGRG2](http://www.uniprot.org/uniprot/Q8CJ12) | [PRASTERONE](../../../ligand/205113/info) | Agonist | <7XKD> |
| Cryo-EM | Class B2 | [AGRG2](http://www.uniprot.org/uniprot/Q8CJ12) | [PRASTERONE](../../../ligand/205113/info) | Agonist | <7XKF> |
| Cryo-EM | Class B2 | [AGRG2](http://www.uniprot.org/uniprot/Q8CJ12) | [PRASTERONE](../../../ligand/205113/info) | Agonist | <7XKE> |
| Cryo-EM | Class B2 | [AGRG3](http://www.uniprot.org/uniprot/Q86Y34) | [HYDROCORTISONE](../../../ligand/142647/info) | Agonist | <7D77> |
| Cryo-EM | Class B2 | [AGRG3](http://www.uniprot.org/uniprot/Q86Y34) | [Beclomethasone](../../../ligand/217272/info) | Agonist | <7D76> |

Supplementary Table 3. 19 Solved class C GPCR structures complexed with synthetic orthosteric modulators

| Structure Type | GPCR Type | GPCR | Modulator | Modulator Type | PDB code |
| --- | --- | --- | --- | --- | --- |
| Cryo-EM | Class C | [mGlu3](../../../protein/grm3_human)R | [LY341495](../../../ligand/2374/info) | Antagonist | <7WI8> |
| Cryo-EM | Class C | [mGlu3](../../../protein/grm3_human)R | [LY341495](../../../ligand/2374/info) | Antagonist | <7WI6> |
| Cryo-EM | Class C | [mGlu3](../../../protein/grm3_human)R | [CHEMBL4081453](../../../ligand/157101/info) | Agonist | <7WIH> |
| Cryo-EM | Class C | [CaS](../../../protein/casr_human)R | [NPS 2143](../../../ligand/2823/info) | Antagonist | <7SIN> |
| Cryo-EM | Class C | [mGlu5](../../../protein/grm5_human)R | [LY341495](../../../ligand/2374/info) | Antagonist | <7FD9> |
| Cryo-EM | Class C | [mGlu5](../../../protein/grm5_human)R | [quisqualate](../../../ligand/3200/info) | Agonist | 7FD8 |
| Cryo-EM | Class C | [mGlu2](../../../protein/grm2_human)R | [LY341495](../../../ligand/2374/info) | Antagonist | 7MTQ |
| Cryo-EM | Class C | [mGlu2](../../../protein/grm2_human)R | [L-glutamic acid](../../../ligand/2276/info) | Agonist | 7MTR |
| Cryo-EM | Class C | [mGlu2](../../../protein/grm2_human)R | [eglumegad](../../../ligand/1504/info) | Agonist | <7EPB> |
| Cryo-EM | Class C | [mGlu2](../../../protein/grm2_human)R | [eglumegad](../../../ligand/1504/info) | Agonist | <7E9G> |
| Cryo-EM | Class C | [mGlu4](../../../protein/grm4_human)R | [L-serine-O-phosphate](../../../ligand/2319/info) | Agonist | <7E9H> |
| Cryo-EM | Class C | [GABAB2](../../../protein/gabr2_human)R | [(-)-baclofen](../../../ligand/562/info) | Agonist | <7EB2> |
| Cryo-EM | Class C | [GABAB1](../../../protein/gabr1_human)R | [CGP55845](../../../ligand/217256/info) | Inverse agonist | <6W2Y> |
| Cryo-EM | Class C | [GABAB2](../../../protein/gabr2_human)R | [CGP55845](../../../ligand/217256/info) | Inverse agonist | <6W2X> |
| Cryo-EM | Class C | [GABAB2](../../../protein/gabr2_human)R | [CGP 54626A](../../../ligand/880/info) | Antagonist | <7C7S> |
| Cryo-EM | Class C | [GABAB2](../../../protein/gabr2_human)R | [(-)-baclofen](../../../ligand/562/info) | Agonist | <7C7Q> |
| Cryo-EM | Class C | [GABAB2](../../../protein/gabr2_human)R | [CHEMBL112710](../../../ligand/9533/info) | Agonist | <6UO8> |
| Cryo-EM | Class C | [GABAB2](../../../protein/gabr2_human)R | [CHEMBL112710](../../../ligand/9533/info) | Agonist | <6UO9> |
| Cryo-EM | Class C | [mGlu5](../../../protein/grm5_human)R | [quisqualate](../../../ligand/3200/info) | Agonist | <6N51> |

Supplementary Table 4. 8 Solved class F GPCR structures complexed with synthetic orthosteric modulators

| Structure Type | GPCR Type | GPCR | Modulator | Modulator Type | PDB code |
| --- | --- | --- | --- | --- | --- |
| Cryo-EM | Class F | SMO | [Cholesterol](../../../ligand/217218/info) | Agonist | <8CXO> |
| X-ray diffraction | Class F | SMO | [3-chloro-N-[trans-4-(methylamino)cyclohexyl]-N-{[3-(pyridin-4-yl)phenyl]methyl}-1-benzothiophene-2-carboxamide](../../../ligand/217338/info) | Antagonist | <7ZI0> |
| Cryo-EM | Class F | SMO | [Cholesterol](../../../ligand/217218/info) | Agonist | <6XBK> |
| Cryo-EM | Class F | SMO | [24,25-Epoxy-cholesterol](../../../ligand/217234/info) | Agonist | <6XBM> |
| Cryo-EM | Class F | SMO | [Cholesterol](../../../ligand/217218/info) | Agonist | <6XBJ> |
| Cryo-EM | Class F | SMO | [Cholesterol](../../../ligand/217218/info) | Agonist | <6XBL> |
| X-ray diffraction | Class F | SMO | [SAG21k](../../../ligand/217236/info) | Agonist | <6O3C> |
| Cryo-EM | Class F | SMO | 24,25-Epoxy-cholesterol | Agonist | <6OT0> |
| X-ray diffraction | Class F | SMO | [Cholesterol](../../../ligand/217218/info) | Agonist | <6D35> |
| X-ray diffraction | Class F | SMO | [cyclopamine](../../../ligand/1254/info) | Partial agonist | <6D32> |
| X-ray diffraction | Class F | SMO | [N-methyl-N-[1-[4-(2-methylpyrazol-3-yl)phthalazin-1-yl]piperidin-4-yl]-4-nitro-2-(trifluoromethyl)benzamide](../../../ligand/217198/info) | Antagonist | <5V56> |
| X-ray diffraction | Class F | SMO | [N-methyl-N-[1-[4-(2-methylpyrazol-3-yl)phthalazin-1-yl]piperidin-4-yl]-4-nitro-2-(trifluoromethyl)benzamide](../../../ligand/217198/info) | Antagonist | <5V57> |
| X-ray diffraction | Class F | SMO | [vismodegib](../../../ligand/3922/info) | Antagonist | <5L7I> |
| X-ray diffraction | Class F | SMO | [SAG1.5](../../../ligand/217186/info) | Agonist | <4QIN> |
| X-ray diffraction | Class F | SMO | [ANTA XV](../../../ligand/424/info) | Antagonist | <4QIM> |
| X-ray diffraction | Class F | SMO | [cyclopamine](../../../ligand/1254/info) | Antagonist | <4O9R> |
| X-ray diffraction | Class F | SMO | [SANT-1](../../../ligand/3397/info) | Antagonist | <4N4W> |
| X-ray diffraction | Class F | SMO | [taladegib](../../../ligand/3674/info) | Antagonist | <4JKV> |

Supplementary Table 5. 1 Solved class T GPCR structure complexed with a synthetic orthosteric modulator

| Structure Type | GPCR Type | GPCR | Modulator | Modulator Type | PDB code |
| --- | --- | --- | --- | --- | --- |
| Cryo-EM | Class T | [TAS2R46](../../../protein/t2r46_human) | [strychnine](../../../ligand/3624/info) | Agonist | 7XP6 |
